# Laccase‐Catalyzed Dimerization of Honokiol and Magnolol for Multitarget Metabolic Enzyme Inhibitors

**DOI:** 10.1002/cbic.70287

**Published:** 2026-04-04

**Authors:** Claudia Sciacca, Nunzio Cardullo, Luana Pulvirenti, Simona Varriale, Libera Vitiello, Cinzia Pezzella, Vera Muccilli

**Affiliations:** ^1^ Department of Chemical Sciences University of Catania Catania Italy; ^2^ Institute of Biomolecular Chemistry, National Research Council ICB‐CNR Catania Italy; ^3^ Department of Chemical Sciences University of Naples Federico II Napoli Italy; ^4^ Institute for Polymers, Composites and Biomaterials, National Research Council IPCB‐CNR Catania Italy

**Keywords:** α‐amylase inhibition, α‐glucosidase inhibition, biomimetic reaction, Box Behnken design, enzymes, lipase inhibition, natural products

## Abstract

Laccases are biocatalysts with a high potential for developing green procedures to achieve new compounds. This study employed laccases in buffer solution to synthesize dimeric neolignans from honokiol and magnolol. Four laccases (LTV, POXA1b, EV3, and EV4) were first screened for honokiol dimerization in the presence of the mediator, with the variant EV4 achieving with the highest yield and in a shorter timeframe the natural houpulin A and B. Reaction conditions were optimized using a mathematical model to maximize the recovery of the desired products, achieving up to 3.42% yield. Interestingly, the optimized conditions also afforded the synthesis of other dimeric compounds, a magnolol dimer and a mixed honokiol–magnolol dimer with comparable yields. In silico studies investigated the substrate's compatibility with the EV4 binding site, highlighting the noncovalent interactions that enhance the stability of the radical formation, thus supporting the production of the obtained products. The four dimeric compounds were studied as metabolic enzyme inhibitors (lipase, α‐amylase, and α‐glucosidase) by in vitro and in silico experiments. The dimers exhibited more potent inhibitory activity than honokiol and magnolol, with houpulin B showing the strongest inhibition toward all tested enzymes.

## Graphical abstract

Laccase‐Catalyzed Dimerization: An exploration of fungal laccases as sustainable biocatalysts for the synthesis of bioactive neolignan dimers of honokiol and magnolol. The optimization of the dimerization conditions reveals an efficient approach to obtain natural and new dimers, whose improved in vitro inhibitory activity against metabolic enzymes highlights their potential application for treating metabolic diseases.

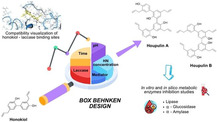

## 1 Introduction

Many natural dimeric and oligomeric compounds are biosynthesized through oxidative coupling by peroxidases, laccases, or cytochrome P450 [1, 2, 3, 4]. These enzymes facilitate the formation of phenoxy radicals, which undergo different mesomeric structures, evolving into other products [5]. Among these, laccases (EC 1.10.3.2; multicopper oxidoreductases) oxidize various phenolic compounds using oxygen and yielding a large variety of bioactive compounds [6, 7, 8, 9, 10, 11], with water as the only by‐product [12, 13, 14, 15]. Inspired by Nature, laccases have been exploited in the laboratory as green biocatalysts to synthesize new bioactive compounds, including lignans and neolignans, phenazines, naphthoquinones, benzimidazoles, and bi‐aryls [1]. Honokiol (HN, **1**) and magnolol (MG, **2**), the most prevalent neolignans in *Magnolia* species, are well known for their biological properties [16, 17, 18, 19, 20, 21, 22, 23, 24, 25, 26]. Oligomeric compounds such as houpulin A (**3**) e B (**4**), related to honokiol, have been isolated from the stem bark and roots of *Magnolia officinalis* with far less than 1% extraction yields [27, 28, 29]. These compounds have been studied for different biological activities, including their effects on superoxide anion generation and elastase release [28], α‐glucosidase inhibition [30], and antitumor properties [27]. To date, only one study has reported the synthesis of oligomers **3** and **4**, starting from honokiol and utilizing peroxidase [30]. Herein, a mathematical approach has been applied to laccase‐mediated reaction in eco‐friendly conditions to optimize the synthesis of **3** and **4**. Specifically, after a comparison of various laccases, response surface methodology (RSM) provided deeper insights into the relationships between multiple independent variables (e.g., honokiol concentration, reaction time, and pH) and the dependent response (%yield) while minimizing the number of experiments required. The optimized conditions were also used to synthesize other dimers. Finally, the synthesized dimers were evaluated as potential candidates for treating obesity and type II diabetes in comparison with their natural precursors honokiol and magnolol.

## 2 Results and Discussion

### 2.1 Preliminary Screening

**SCHEME 1:**
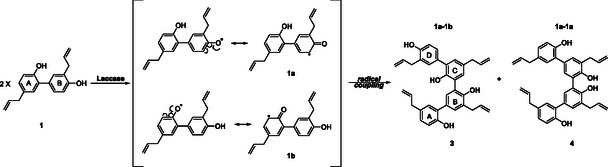
Mechanism of laccase‐mediated dimerization of **1**.

An initial screening of various laccases in synthesizing compounds **3** and **4**, starting from honokiol (Scheme 1) facilitated the identification of the most effective enzyme for developing the mathematical model. A commercially available fungal laccase from *Trametes versicolor* (LTV) and a recombinant one from *Pleurotus ostreatus* (POXA1b) together with its two evolved mutants (EV3 and EV4 [31]) were tested. POXA1b is a high‐redox potential laccase extensively used over various industries thanks to its characteristics such as stability and enzymatic activity over a broad pH range (3–9) and temperature range (25°C–65°C), as well as its high expression levels in heterologous hosts [31]. The redox potentials of POXA1b have been reported to be approximately +650 mV [32], while LTV shows a redox potential of +650 mV [33]. The employed evolved variants display improved phenotypes in terms of pH stability [32] and increased redox potential [34]. The evolved mutant EV3 exhibits a redox potential of +770 mV [34], whereas no redox potential value has been reported for the EV4 variant. All reactions were carried out employing 12 U of each enzyme in 0.1 M phosphate buffer, pH 6.0 [35, 36] at room temperature, with and without the addition of the mediator 2,2′‐azino‐bis(3‐ethylbenzothiazoline‐6‐sulfonic acid) (ABTS) [1, 37] following the yields of **3** and **4** by up to 120 h (**Table S2**). All reactions were monitored by high performance liquid chromatography‐ultraviolet (HPLC‐UV), and the desired dimers were isolated by semipreparative HPLC separation. The tested enzymes exhibited the following performance order: EV4 > EV3 > POXA1b > LTV, with EV4 achieving the highest yields of **3** and **4** (3.0%) within 2 h in the presence of ABTS. Compared to LTV, POXA1b and its variants, EV4 achieved higher yields in a shorter timeframe (2 h vs. 96 h), with a notable impact from the addition of the mediator. Doubling the LTV concentration did not result in any significant improvement in either yield or production time. A general trend was observed for reactions catalyzed by POXA1b, EV3, and EV4: the yields of products **3** and **4** did not increase with reaction time. Instead, lower amounts of the expected compounds were detected as the reaction progressed, despite complete substrate consumption being observed at 96 h (data not shown). Matrix‐assisted laser desorption‐ionization (MALDI) mass spectra obtained from reaction mixtures sampled at different time points (**Figures S1**–**S4**) revealed that extending the reaction time led to the formation of oligomers, consistent with the yield decrease indicated by the HPLC‐UV results. Additional experiments testing another mediator ((2,2,6,6‐tetramethylpiperidin‐1yl)oxyl (TEMPO), data not shown) and organic solvents (**Table S1**), to enhance substrate solubilization [23], did not improve the yields. Based on the above outcomes, EV4 was chosen as catalyst for the optimization study in organic solvent‐free conditions, thus ensuring an environmentally friendly and sustainable approach. The focus was on selecting the optimal substrate concentration, pH, and reaction time, as described below.

### 2.2 Single‐Factor Experiments

**FIGURE 1:**
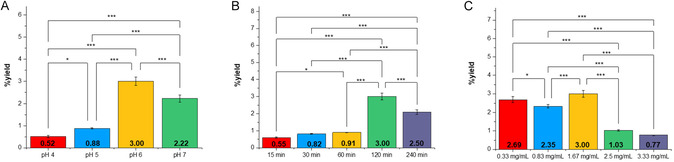
Results of single‐factor experiments on the dimerization reaction yield of HN with EV4. (A) Effect of pH, (B) reaction time, and (C) HN concentration on the reaction yield of houpulin A (**3**) and B (**4**). All dimerization reactions were conducted at 25°C using ABTS as a mediator (0.3 equiv). Data are reported as mean ± standard deviations SD (*n* = 3). Statistical significance was determined using a one‐way ANOVA. *p < 0.05; **p < 0.01; ***p < 0.001 (Turkey's test).

Single‐factor experiments were carried out to select the range of the chosen independent variables affecting the dimerization of HN, thus defining the experimental space. Reactions were carried out by varying one parameter at a time (buffer pH = *X*_1_, reaction time = *X*_2_, and HN concentration = *X*_3_; **Table S3**) while keeping the others fixed (pH 6, 120 min, and 1.67 mg/mL). Figure 1 reports the results as %yield of houpulin A (**3**) and B (**4**) of the single‐factor experiments. As observed, the %yield of both HN dimers improved from 0.52% to 3.0% with the increase of pH, prompting the exploration of an optimal pH between 5 and 7. The yields rose from 0.55% to 3% for **3** and 0.61% to 3% for **4** with reaction time up to 120 min, but then decreased, therefore, time in the range of 60–240 min was selected. Finally, the substrate concentration between 0.33 and 1.67 mg/mL was chosen as the reaction yield decreased at higher HN values.

### 2.3 Model Fitting Experimental Design

Parametric optimization of reaction yield (**Table S5**) was carried out using a Box–Benken design (BBD) approach. As shown in **Table S5**, the %yield obtained for **3** and **4** ranged from 0.88% to 3.1% for the two compounds. The regression analysis was performed using the least square method based on the experimental design (**Table S6**). All the investigated linear coefficients were significant, with a *p*‐value below 0.05. pH had the most critical effect on responses *Y*_1_ and *Y*_2_, as indicated by the high *F*‐values (405.17 for 3 and 430.96 for 4) and low *p*‐values (<0.0001); the quadratic terms *x*_2_ *x*_2_ were also significant. Among the interaction effects, only the combination *x*_2_ *x*_3_ was significant (*p*‐value of 0.0002). Consequently, the model was significant, as indicated by the *p*‐values (<0.0001) and the high *F*‐values of 126.98 and 133.13 for *Y*_1_ and *Y*_2_, respectively. Furthermore, the lack of fit was not significant (>0.05), confirming the model's suitability for optimizing the parameters. R^2^ values close to 1 highlighted a strong correlation between the measured and predicted data. The model's adequacy was further confirmed by the coefficient of variation (CV = 7.11%), which indicated a high reliability of the experimental data and no significant variability. The final predictive equations consider only the significant terms:

*Y*_1_ = −4.5401 + 1.5985*x*_1_ + 0.00070*x*_2_ − 0.4267*x*_3_ − 0.000051*x*_2_^2^ − 0.36759*x*_3_^2^ + 0.0019*x*_1_ *x*_2_ + 0.00570*x*_2_ *x*_3_

*Y*_2_ = −4.3781 + 1.5384*x*_1_ + 0.00018*x*_2_ − 0.3724*x*_3_ − 0.000051*x*_2_^2^ − 0.3816*x*_3_^2^ + 0.002*x*_1_ *x*_2_ + 0.0056*x*_2_ *x*_3_

**FIGURE 2:**
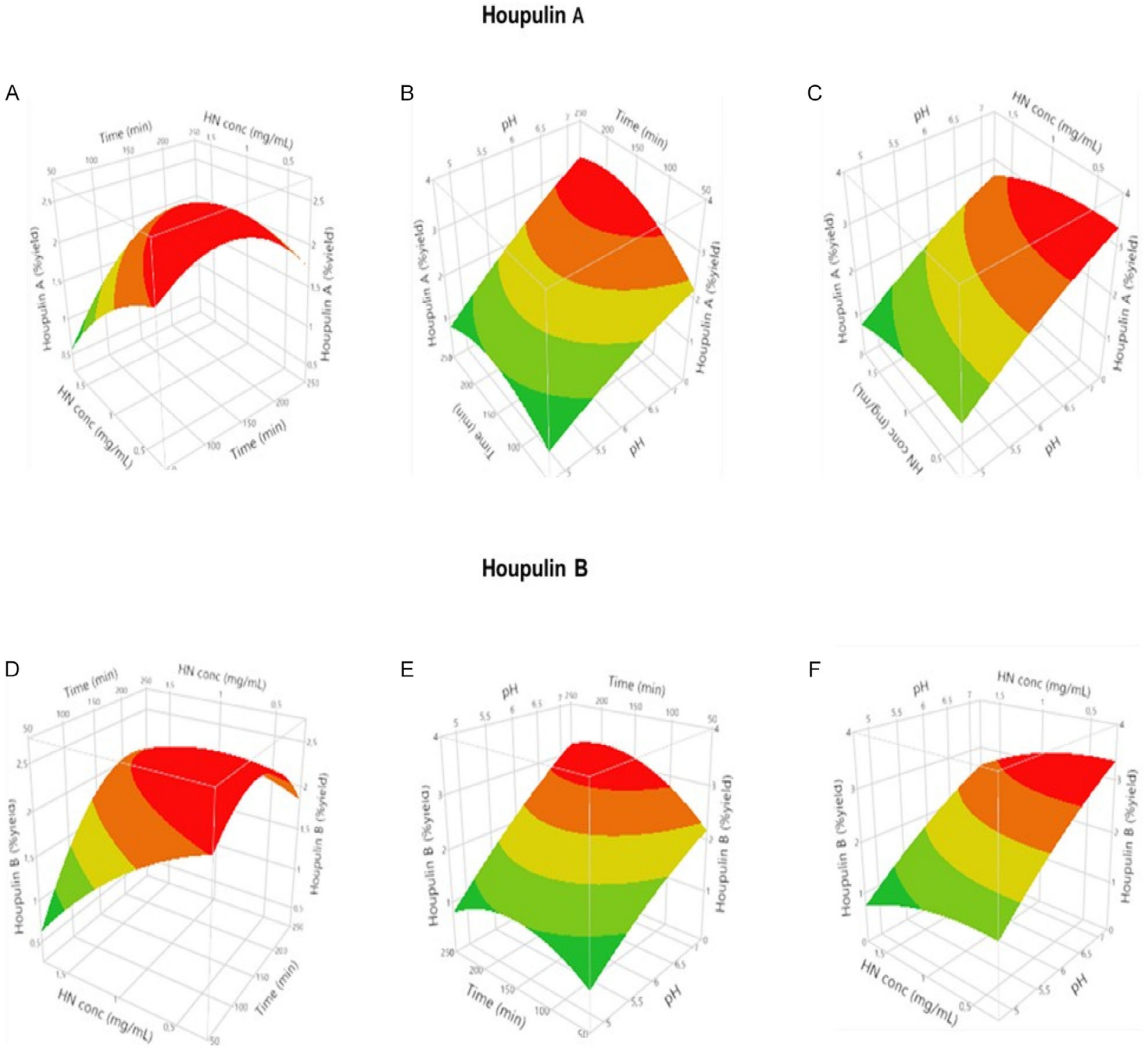
3‐D surface plots illustrate the combined effects of parameters on houpulin **3** and **4** %yield: A and D show the effect of time and HN concentration, B and E report the effect of pH and time, C and F illustrate the effect of pH and HN concentration.

The effects of two‐factor pairs on the studied responses can be effectively visualized using the RSM plots derived from the model's equations, which were generated using a quadratic model, keeping one variable constant at its central level for each plot (Figure 2A–F). The red zone of each surface indicated the optimal conditions for the maximum yield in the contour diagrams. The 3D plots reported in Figure 2A,D, suggested that the yields improved as the reaction time increased to 200 min and then decreased. A similar behavior has been reported by Schirmann et al. [38] for the selective laccase‐mediated dimerization of 2,6‐dimethoxyphenol and by Latif et al. [39] for the degradation of bisphenol A using immobilized LTV. The data presented here revealed an increase in the yields of both dimers as the concentration of honokiol decreased from 1 mg/mL to 0.5 mg/mL. This trend was similarly noted by Schirmann et al. [38]. The low *p*‐values of the *x*_2_ *x*_3_ interaction terms (Table S6) indicate a significant relationship between honokiol concentration and reaction time at a constant pH buffer. The surface responses reported in Figure 2B,E highlighted that the yield increased when the pH and reaction time increased. From the *p*‐value of 0.0809 and 0.0668 for *Y*_1_ and *Y*_2_, respectively, it was inferred that pH and reaction time had no significant relationship, while the honokiol concentration was constant. The surface plots of Figure 2C,F indicated that the reaction yield for **3** and **4** was maximized at lower honokiol concentrations and higher pH values. However, the *p*‐values of 0.6930 for *Y*_1_ and 0.668 for *Y*_2_ suggest that pH and honokiol concentration did not have a significant relationship when the reaction time was constant.

### 2.4 Optimal Reaction Condition and Model Validation

**SCHEME 2:**
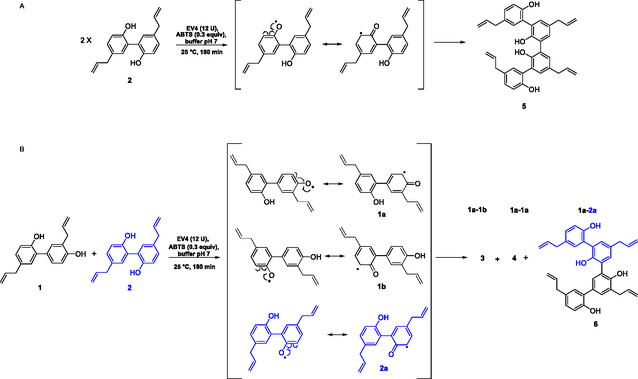
Synthesis of dimers (A) **5**, (B) 3, **4** and **6**.

According to the predictive equations and the regression analysis, the optimal conditions: pH 7, 180 min, and HN concentration of 0.61 mg/mL were employed for synthesizing **3** and **4** with EV4 (12U) and ABTS (0.3 equiv). These optimal factors' combination was experimentally validated by performing the dimerization in triplicate. The results indicated that the experimental data closely matched the predicted values with no significant differences observed (*p* < 0.05, **Table S7**). The two honokiol dimers were synthesized with 3.42% yield, demonstrating that the model improved the reaction yield by ≈14% compared to the yield obtained during the preliminary screening (entry 26, **Table S2**) and that previously reported by He et al. [30] on peroxidase‐mediated dimerization in aqueous acetone over 12 h (**3**: 2.6%; **4**: 3.0%). This underscores the enhanced environmental sustainability of the new reaction conditions established by our approach, which involves the exclusive use of phosphate buffer and reduced reaction time and substrate concentration. The EV4 catalytic ability was also tested for the synthesis of two further dimers (Scheme 2). The dimer **5** was obtained by MG homo‐coupling with 3.1% yield, and its structure was confirmed by a comparison with nuclear magnetic resonance (NMR) data with those previously reported by Sy et al. [40]. Like houpulin B (**4**), it exhibited a symmetrical structure, as the oxidative step produced only a single radical. Conversely, the hetero‐coupling between HN and MG afforded the new dimer **6** with 3.2% yield and houpulin A (**3**) and B (**4**) as homo‐coupling products with 2.0% and 2.8% yield, respectively.

### 2.5 Computational Analysis of Laccase–Neolignans Interactions

**FIGURE 3:**
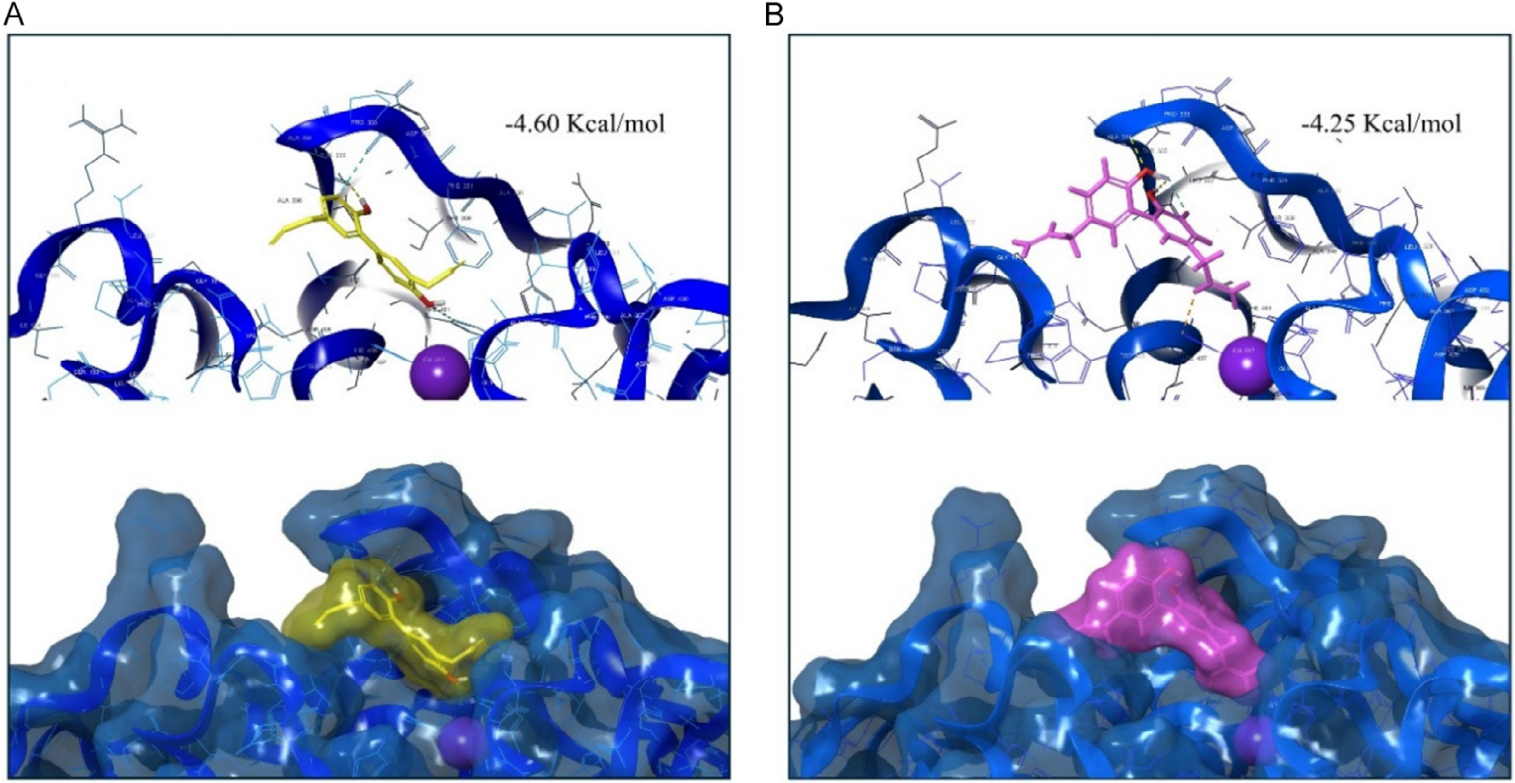
Interaction of EV4 laccase (blue) with: (A) compound **1** (yellow) and (B) compound **2** (magenta) into the enzyme catalytic site. T1 copper is highlighted as a violet sphere. The interactions are depicted in yellow (hydrogen bonds), cyan (aromatic H‐bond), faded azure (pi–pi stacking), and green (pi‐cation).

Molecular docking was performed on **1** and **2**, to investigate the interaction with EV4. The visualization of the substrates’ compatibility with the enzyme's binding sites allowed us to highlight the noncovalent interactions that could enhance binding affinity and stability. The binding energy (Kcal/mol) of the best conformation acquired by the laccase–neolignans complex was calculated, and their localization on the enzyme structure is displayed in Figure 3. In the case of the dimerization of HN, the reaction starts with its oxidation leading to the formation of two radicals (**1a** and **1b**, Scheme 1) arranging into two different positions in EV4 binding pocket, orienting the reactive hydroxyl group toward the Type 1 copper (**1a**) or the outer side of the active site (**1b**). The distance between the hydroxyl group of the substrate and the Type 1 copper in the laccase catalytic site (4.04 Å for **1a**, 10.11 Å for **1b**) is crucial as it directly affects the efficiency of electron transfer, which is the first step in this reaction. The results in atom distances of the two radicals could explain the difference in **3** and **4** synthesis yield, which is higher for **4** composed of two **1a** radicals regardless of the presence of the mediator. Despite a binding energy value between magnolol and EV4 binding site (−4.25 Kcal/mol) comparable with the **1a** HN radical (−4.60 Kcal/mol), the orientation of the MG monomer does not favor the reaction. The measured distance between the two possible reactive hydroxyl groups and the T1 copper is 11.50 and 12.58 Å. As a fact, the occurrence of MG homo‐dimer (**5**) under the applied reaction conditions is possible only in the presence of the mediator.

## 3 In Vitro Enzymes’ Inhibitory Activity and Kinetic Studies

**FIGURE 4:**
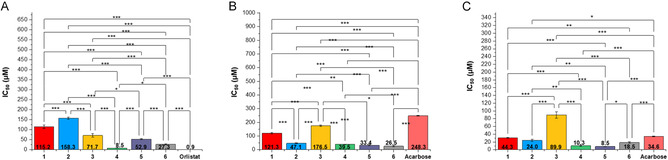
Results of the inhibitory activity of studied compounds toward metabolic enzymes. (A) Inhibitory activity toward PL; (B) Inhibitory activity toward α‐Glu; and (C) Inhibitory activity toward α‐Amy. All data are reported as mean ± standard deviations, SD (*n* = 3). Statistical significance was determined using a one‐way ANOVA. *p < 0.05; **p < 0.01; ***p < 0.001 (Turkey's test).

All compounds were tested for their inhibitory activity toward pancreatic lipase (PL), α‐glucosidase from *Saccharomyces cerevisiae* (α‐Glu), and α‐amylase from porcine pancreas (α‐Amy) [17, 41]. Orlistat (an anti‐obesity drug) and acarbose (an antidiabetic drug) were used as positive controls. Inhibitory activities are reported in Figure 4. The compounds **4**–**6** exhibit enhanced inhibitory effects toward hypoglycemic enzymes outperforming parent compounds **1** and **2** and acarbose, which shows IC_50_ value of 34.6 µM for α‐Amy and 248.3 µM for α‐Glu. In the light of search a compound with inhibitory capability toward all the three metabolic enzymes evaluated, houpulin B (**4**) was the most promising compound, with IC_50_ values of 8.5 µM (PL), 10.3 µM (α‐Amy), and 39.5 µM (α‐Glu). Compound **5**, obtained from magnolol, showed IC_50_ value of 8.5 µM (α‐Amy), 33.4 µM (α‐Glu), and 52.9 µM (PL), displaying the same inhibitory trend observed for the natural lead (**2**). The mixed dimer **6** showed comparable activity toward all enzymes with values between 18.5 and 27.3 µM. Finally, dimer **3** was the least active among all the dimers toward the three enzymes.

**FIGURE 5:**
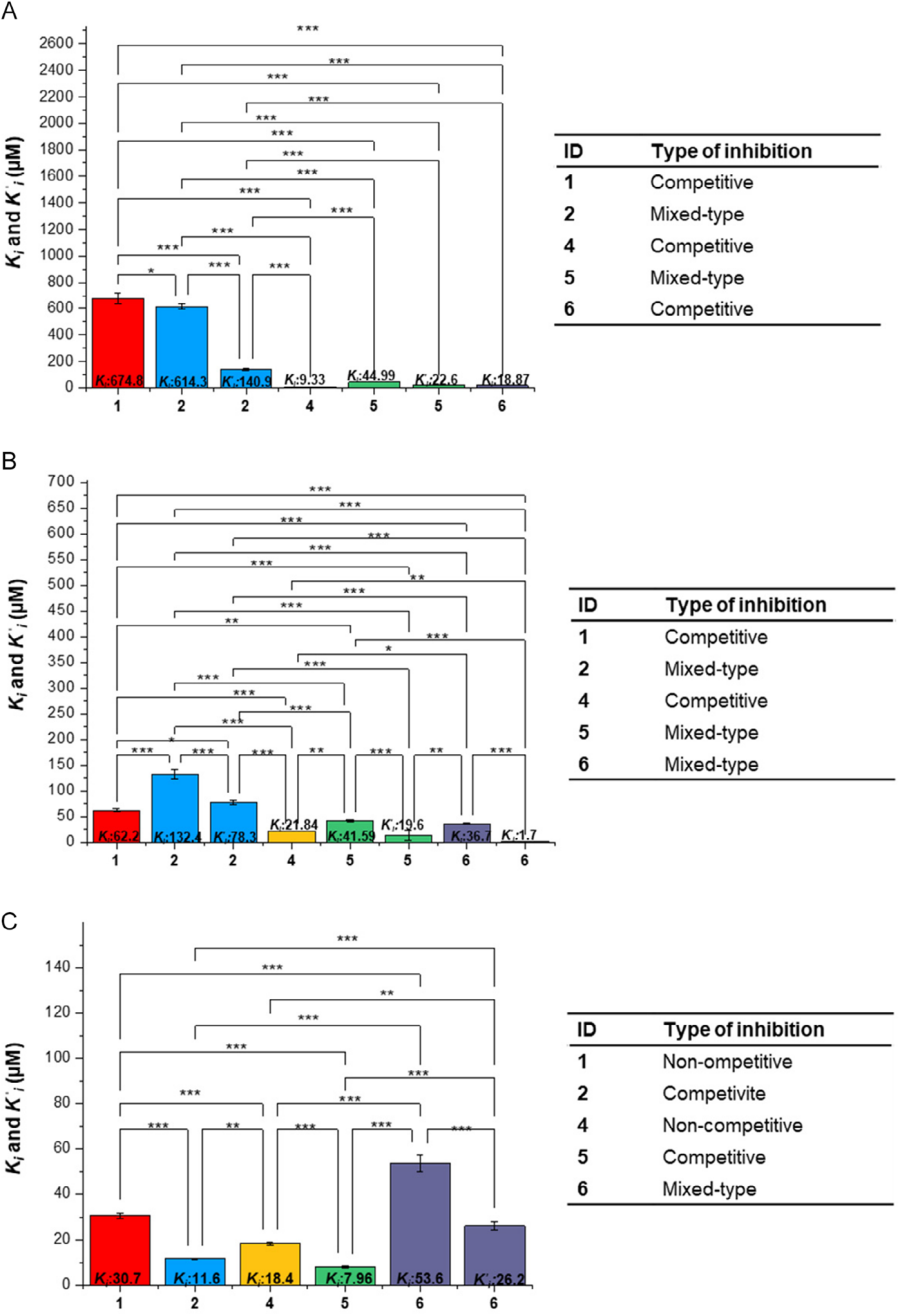
Kinetic inhibition toward metabolic enzymes. (A) Kinetic constants for PL inhibition. Compounds **2** and **5** exhibited mixed‐type inhibition mechanism, therefore, two kinetic constants are reported *K*_*i*_, refers to the formation of enzyme–inhibitor (EI) complex and *K'*_*i*_, corresponds to the formation of the enzyme–substrate–inhibitor (ESI) complex. (B) Kinetic constants for α‐Glu inhibition. Compounds **2**, **5** and **6** exhibited mixed‐type inhibition mechanism and two kinetic constants are reported (*K*_*i*_ and *K'*_*i*_)*.* (C) Kinetic constants for α‐Amy. Compound **6** exhibited mixed‐type inhibition mechanism, and thus two kinetic constants are reported (*K*_*i*_ and *K'*_*i*_)*.* All data are reported as mean ± standard deviations, SD (*n* = 3). Statistical significance was determined using a one‐way ANOVA. *p < 0.05; **p < 0.01; ***p < 0.001 (Turkey's test).

Lineweaver–Burk (L–B) plots (**Figures S34** and **S39**) of the most active compounds **4**–**6** assessed the inhibition modes toward the enzymes. The inhibitory mechanisms of MG and HN against PL were previously reported by our group [17], as well as MG's inhibition of α‐Glu [42]. To ensure thoroughness, the inhibition kinetics of **1** and **2** against α‐Amy and **1** against α‐Glu were examined. All the existing kinetic data, together with the newly acquired, are reported in Figure 5. The inhibition mode of dimers obtained by homo‐coupling matches the one observed for the corresponding monomer. Compound **4** was a competitive inhibitor of PL (*K*_*i*_ = 9.33 µM) and α‐Glu (*K*_*i*_ = 21.84 µM), whereas it inhibited α‐Amy with a noncompetitive mechanism (*K*_*i*_ values of 18.4 µM), such as HN. Similarly, **5** inhibited PL and α‐Glu with a mixed‐type mechanism (PL: *K*_*i*_ = 44.99 µM and *K'* *_i_* = 22.60 µM; α‐Glu: *K*_*i*_ = 41.59 µM and *K'*_*i*_ = 19.6 µM), and α‐Amy competitively (*K*_*i*_ = 7.96 µM), showing the same behavior of MG. Lastly, the dimer **6** obtained by hetero‐coupling inhibited PL competitively (*K*_*i*_ = 18.87 µM), and both α‐Glu (*K*_*i*_ = 36.7 µM and *K'*_*i*_ = 1.7 µM) and α‐Amy (*K*_*i*_ = 53.6 µM and *K'*_*i*_ = 36.7 µM) in a mixed‐type manner. The *K*_*i*_ values were consistent with the inhibitory assays, confirming that compound **4** is the most promising inhibitor among the dimers studied.

## 4 Computational Analysis of Metabolic Enzymes–Neolignans Interaction

**FIGURE 6:**
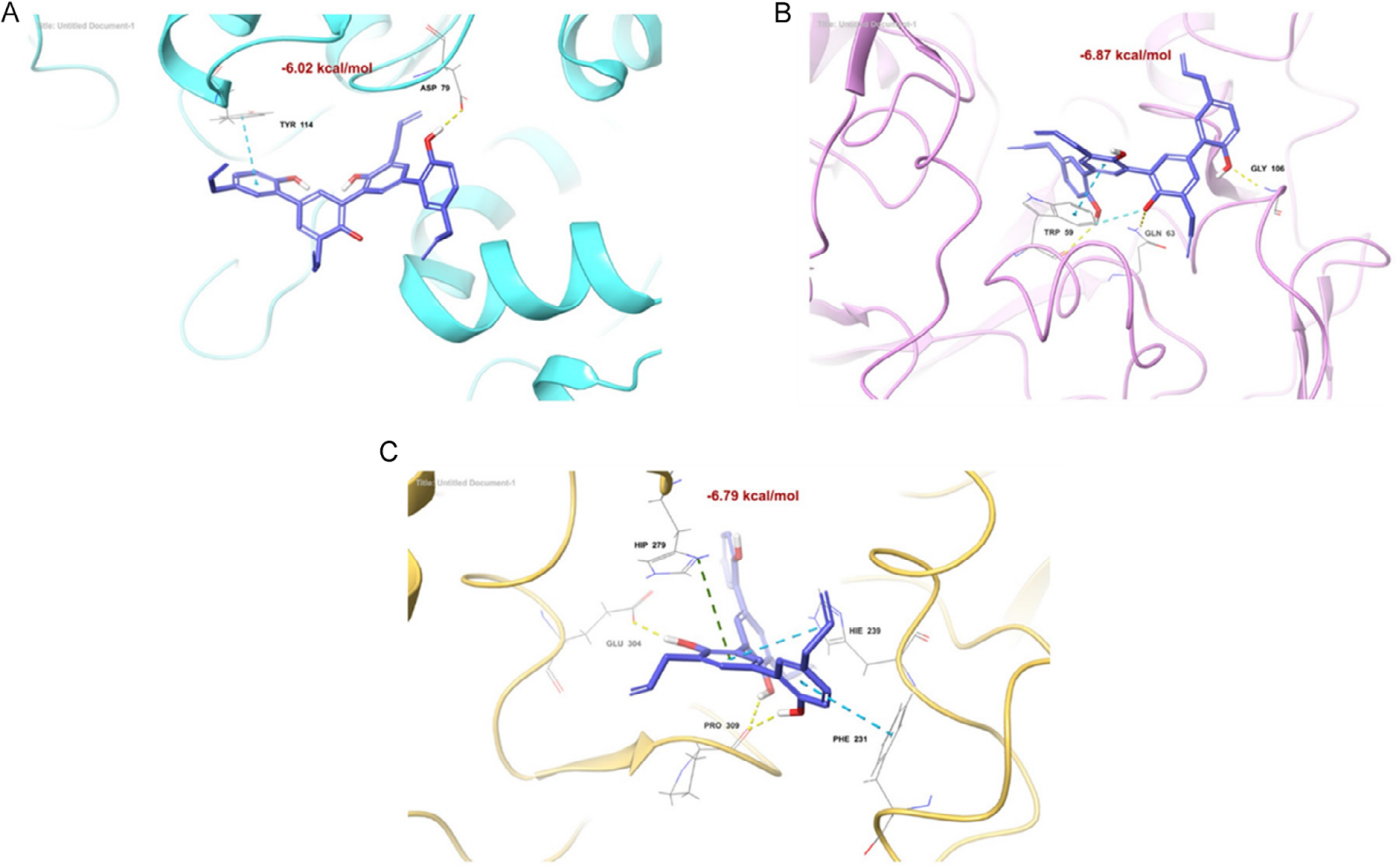
Compound **4** (faded blue) into (**A**) PL (cyan binding site); (**B**) α‐Amy (magenta binding site); (**C**) α‐Glu (faded‐orange binding site). The interactions are depicted in yellow (hydrogen bonds), cyan (aromatic H‐bond), faded azure (pi–pi stacking), and green (pi‐cation); only interactive residues are labeled. The color coding used for atoms is red (O), blue (N), and gray (C).

Molecular docking studies were performed on **1** and **2** and the synthesized dimers (**3**–**6**) to investigate their interactions with α‐Glu, α‐Amy, and PL (**Figures** S40–S57, **Tables** S8–S11). For PL, the synthesized dimers showed greater spatial compatibility in the binding pocket (**Figures** S40–S42) due to their larger size than the natural leads previously investigated [17]. Houpulin A (**3**) remained partially exposed outside the pocket, reducing its stability within the PL binding site and supporting the weak PL inhibition (IC_50_ 71.7 µM). In contrast, its isomer **4** emerged as a potent inhibitor with significant binding affinity (−0.02 Kcal/mol) also confirmed by the IC_50_ of 8.5 µM; it established hydrogen bonds with Asp79 and π‐π stacking with Tyr114, key residues that contribute to its effectiveness (Figure 6A). MG dimer (**5**; IC_50_ 52.9 µM) showed a similar orientation to **4** inside the catalytic site, establishing the same type of interactions. The HN–MG dimer (**6**; IC_50_ 27.3 µM) displayed similar interactions to **4** but lacked the π‐π stacking due to its orientation, thus supporting the lower inhibitory activity with respect to **4**. In α‐Amy binding studies (Figures S43–S45), neolignans **1** and **2** showed a good affinity for the enzyme's catalytic triad (Asp197, Asp300, and Glu233) [43], similar to the standard inhibitor acarbose. However, their dimeric counterparts (**3**–**6**) interacted differently, establishing noncovalent interactions with other residues and showing generally higher binding energies. Dimer **3** (IC_50_ 89.9 µM) showed stabilizing interactions with Trp 59, as isomer **4**, but employed a distinct orientation that may justify the lower inhibitory activity than **4**. Compound **4** (IC_50_ 10.3 µM) was notable for its binding affinity (−6.87 Kcal/mol) establishing hydrogen bonds with Gln63 and Trp59, and π‐π stacking with Trp59 (**Figure** 6B). Compound **5** (IC_50_ 8.5 µM) formed a stable complex with α‐Amy establishing the same interaction of **4** with Trp 59 and an additional H‐bonds with Val163 and Trp59, thus corroborating the results showed by in vitro inhibition. Dimer **6**, showing a similar orientation to **5**, had less interaction, thus justifying the weaker inhibitory activity (18.5 µM) respect to **5** (8.5 µM). For α‐Glu, docking studies revealed strong affinities of compounds **4**–**6**, with binding energies between −6.03 and −6.79 Kcal/mol, whereas **3** had a Δ*G*_bind_ of 5.43 Kcal/mol (**Figures** S46–S48), clearly supporting the highest IC_50_ value registered (176.5 µM) among the tested dimers. This compound is also differently located in the catalytic pocket with respect to the other dimers, forming three interactions with Phe157, Arg312, and Pro309. Compound **4**, which exhibited the highest affinity, established a network of interactions involving all four aromatic rings, primarily H bonds through OH groups, contributing significantly to complex stabilization (Figure 6C). **5** displayed hydrogen bonds with Asp349, a catalytic triad residue in α‐Glu, in addition to other interactions, fitting deeper into the binding pocket. Ligand **6**, the most active α‐Glu inhibitor (IC_50_ 26.5 µM), established seven noncovalent interactions, including hydrogen bonds and π‐cation interactions, providing significant stabilizing effects. Although molecular docking is not inherently designed to distinguish inhibition mechanisms, our results can offer some structural insights. Competitive inhibitors, such as compound **4**, were found to bind within the catalytic site of both α‐Amy and α‐Glu (Figure 6B, C). The docked pose of **4** showed strong interactions with key catalytic residues, such as Asp197, Glu233, and Asp300 in α‐Amy, and Asp349, Glu276, and Arg439 in α‐Glu. These interactions are consistent with its substrate‐mimicking behavior, indicating a direct competition for the active site.

## 5 Conclusion

As part of the exploration of new sustainable methods for synthesizing bioinspired compounds, organic solvent‐free conditions for the oxidative coupling reactions of HN and MG, mediated by laccase‐ABTS cooperation, have been reported. Preliminary reactions have highlighted that the recombinant laccase variant EV4 (from *Pleurotus ostreatus*) is more suitable for the synthesis of natural Houpulin A and B, respect to LTV, POXA1b, and EV3. The BBD was used to optimize three reaction conditions (HN concentration, pH, and reaction time), thus affording the natural compounds with higher reaction yield by ≈14% than that of preliminary reactions. Their formation was rationalized by molecular docking investigation, studying binding energies of **1** and **2** with EV4 catalytic site (T1 copper) and analyzing noncovalent interactions and related distances, as important factors for radical formation. Although these yields slightly exceed 3%, they are significantly higher than the extraction yields obtained through unsustainable processes used to isolate compounds **3** and **4**. The optimized procedure applied to homo‐ and hetero‐coupling reactions with **1** and **2** afforded two dimers (**5** and **6**) in addition to **3** and **4**. Compounds **4**, **5,** and **6**, assessed for the inhibition of PL, α‐Amy, and α‐Glu, revealed more effective metabolic enzyme inhibitors than natural HN and MG. This work paves the way to explore environmentally friendly conditions based on the use of laccase as an alternative way to conventional synthesis and extraction procedures to achieve bioactive compounds.

## 6 Experimental Section

### 6.1 Experimental Design for Optimization of Dimerization Reaction Conditions

RSM was employed using a three‐factorial BBD to assess the effects of operating variables (*X*_1_–*X*_3_) on the dimerization reaction conditions. The variables tested included buffer pH, reaction time, and HN concentration, which were modified in each experiment, as reported in **Table S2**. A total of 15 experimental runs were conducted following the design equation:

(1) $$N=2k\left(k-1\right)C_{0}$$

where *k* is the number of factors, and *C*_0_ is the number of the central points. Three levels were selected for each factor under investigation: low (**−**1), middle (0), and high (+1). The dimerization reactions were randomly performed to reduce the effect of unexplained variability in the measured responses caused by systematic errors. The dependent variables were *Y*_1_ and *Y*_2,_ respectively, % yield for the synthesis of **3** and **4** that were fitted using a second‐order quadratic equation:

(2) $$Y=B_{0+}\sum_{i=1}^{k}B_{i}X_{i}+\sum_{i=1}^{k}B_{ii}X^{2}+\sum_{i=1}^{k}B_{ij}X_{i}X_{ij}+E$$

where *Y* is the predicted response, *X* is the independent variable, *B*_0_ is the intercept; *B*_*i*_, *B*_*ii*_, and *B*_*ij*_ are respectively, the linear, quadratic, and interactive regression coefficients. Three‐dimensional response surface plots were used as visual tools to examine the interaction factors and responses, thus deducing the optimal combinations of factors to enhance reaction yield. Model adequacy was assessed through the coefficient of determination (R^2^), *F*‐test values, and the lack of fit test. The optimized reaction condition was subsequently applied in triplicate for the homo‐coupling of **1**, of **2**, and for hetero‐coupling.

### 6.2 Laccase‐Mediated Dimerization at Optimized Conditions

**1** or **2** (100 mg, 0.38 mmol) was solubilized in 160 mL of phosphate buffer (0.1 M, pH = 7) aided by sonication. Subsequently, EV4 (14.5 mg) and ABTS (58.6 mg, 0.3 equiv) were added to the starting mixture. The reaction was incubated at 25°C and 150 rpm for 180 min. The mixture was partitioned with ethyl acetate (3 × 60 mL). The organic layer was partitioned with H_2_O, dried using anhydrous Na_2_SO_4_, filtrated, and evaporated under reduced pressure. The expected compounds were recovered after HPLC‐UV purification (see Supporting Information).

### 6.3 3′′′, 5,5′, 5″‐Tetraallyl‐[1,1′:3′, 1″:3″, 1′′′‐Quaterphenyl]−2,2″, 4′, 4′′′‐Tetraol (3)

Compound **3** was achieved after HPLC‐UV purification (4 mg; 3.42%) with 98% purity as a brownish oil. The obtained spectroscopic data matched those reported by Shih et al. [28]. ^1^HNMR (500 MHz, (CD_3_)_2_CO, 25°C, TMS, ppm): *δ* = 7.42 (d, *J =* 2.3 Hz, 2H, Ar‐H, H2′, and H2″), 7.38 (dd, *J =* 2.3 Hz, 2H, Ar‐H, H6′, and H6″), 7.14 (dd, *J* = 2.2 Hz, 2H, Ar‐H, H6, and H2′′′), 6.98 ( dd, *J* = 8.2, 2.3 Hz, 2H Ar‐H, H4, and H6′″), 6.90 (d, *J* = 8.1 Hz, 2H, Ar‐H, H3, and H5′′′), 6.01 (m, 4H, CH, H8, H8′, H8″, and H8′′′), 5.17 (dd, *J* = 17.1, 2.0 Hz, 4H, CH, H_a_9, H_a_9′, H_a_9″, and H_a_9′′′), 4.99 (dd, *J* = 10.1, 2.0 Hz, 4H, CH, H_b_9, H_b_9′, H_b_9″, and H_b_9′′′), 3.55 (d, *J* = 6.8 Hz, 4H, CH_2_, H7′, and H7″), and 3.34 (d, *J* = 6.7 Hz, 4H, CH_2_, H7, and H7′′′). ^13^CNMR (125 MHz, (CD_3_)_2_CO, 25°C, TMS, ppm): *δ* = 155.3 (C4′′′),153.2 (C2), 152.0 (C4′), 149.7 (C2″), 139.3 (CH, C8), 139.0 (CH, C8″), 138.2 (C8′′′), 138.0 (C8′), 133.2 (C5″), 132.2 (C5), 131.7 (CH, C2′′′), 131.6 (C1′′′), 131.4 (CH, C6), 131.3 (CH, C2′), 131.3 (CH, C4″), 131.2 (CH, C6″), 131.2 (CH, C6′), 131.0 (C1′), 130.6 (C1′′′), 129.0 (CH, C2′′′), 128.9 (C1), 128.8 (CH, C4), 128.6 (C5′), 127.6 (C3′), 127.0 (C1″), 126.6 (C5″), 116.9 (CH, C3), 115.9 (CH_2_, C5′′′), 115.8 (CH_2_, C9′), 115.6 (CH_2_, C9″), 115.4 (CH_2_, C9), 115.6 (CH_2_, C9′′′), 40.1 (CH_2_, C7), 40.0 (CH_2_, C7), 35.5 (CH_2_, C7′), and 35.5 (CH_2_, C7′′′). HRMS m/z calcd for C_36_H_34_NaO_4_: 553.2355 [M+Na]^+^, found 553.2369.

### 6.4 5,5′, 5′′, 5′′′‐Tetraallyl‐[1,1′:3′, 1′′:3″, 1′′′‐Quaterphenyl]−2,2′′′, 4′, 6″‐Tetraol (4)

Compound **4** was achieved after HPLC‐UV purification (4 mg; 3.42%) with 98% purity as a brownish oil. The obtained spectroscopic data matched those reported by Shih et al. [28]. ^1^HNMR (500 MHz, (CD_3_)_2_CO, 25°C, TMS, ppm): *δ* = 7.40 (dd, *J =* 2.3 Hz, 2H, Ar‐H, H2′, and H2′′′), 7.38 (dd, *J =* 2.3 Hz, 2H, Ar‐H, H4′, and H6″), 7.14 (dd, *J* = 2.2 Hz, 2H, Ar‐H, H6, and H6′′′), 6.98 (dd, *J* = 8.2, 2.3 Hz, 2H, Ar‐H, H4, and H4′′′), 6.90 (d, *J* = 8.1 Hz, 2H, Ar‐H, H3, and H3′′′), 6.11 (m, 2H, CH, H8, and H8′′′), 5.98 (m, 2H, CH, H8′, and H8″), 5.17 (dd, *J* = 17.1, 2.0 Hz, 4H, CH, H_a_9, H_a_9′, H_a_9″, and H_a_9′′′), 4.99 (dd, *J* = 10.1, 2.0 Hz, 4H, CH, H_b_9, H_b_9′, H_b_9″, and H_b_9′′′), 3.55 (d, *J* = 6.8 Hz, 4H, CH_2_, H7′, and H7″), and 3.34 (d, *J* = 6.7 Hz, 4H, CH_2_, H7, and H7′′′). ^13^CNMR (125 MHz, (CD_3_)_2_CO, 25°C, TMS, ppm): δ = 152.4 (C2 and C2′′′), 151.0 (C4′ and C6″), 138.4 (CH, C8 and C8′′′), 137.1 (CH, C8′ and C8″), 131.2 (C5 and C5′′′), 130.9 (C1 and C1′′′), 130.47 (CH, C2′ and C2′′′), 130.45 (CH, C4′ and C6″), 130.4 (CH, C6 and C6′′′), 128.1 (C1′ and C3″), 128.0 (CH, C4 and C4′′′), 127.7 (C5′ and C5″), 125.5 (C3′ and C1″), 116.0 (CH, C3 and C3′′′), 114.9 (CH_2_, C9 and C9′′′), 114.5 (CH_2_, C9′ and C9″), 39.1 (CH_2_, C7 and C7′′′), and 34.6 (CH_2_, C7′ and C7″). HRMS m/z calcd for C_36_H_34_NaO_4_: 553.2355 [M+Na]^+^, found 553.2369.

### 6.5 5,5′, 5′′, 5′′′‐Tetraallyl‐[1,1′:3′, 1′′:3′′, 1′′′‐Quaterphenyl]−2,2′, 2′′, 2′′‐Tetraol (5)

Compound **5** was achieved after HPLC‐UV purification (3.1 mg; 3.1%) with 98% purity as brownish oil. Spectroscopic data were congruent with those reported by Sy et al. [40]. ^1^HNMR (500 MHz, (CD_3_)_2_CO, 25°C, TMS, ppm): *δ* = 7.27 (dd, *J =* 2.3 Hz, 2H, Ar‐H, H6′, and H6″), 7.20 (dd, *J =* 2.3 Hz, 2H, Ar‐H, H6, and H6″), 7.14 (dd, *J* = 2.2 Hz, 2H, Ar‐H, H6, and H6′′′), 7.13 ( d, *J* = 8.1 Hz, 2H, Ar‐H, H4′, and H4″), 6.97 (dd, *J* = 8.1, 2.2 Hz, 2H, Ar‐H, H4, and H4′′′), 6.77 (dd, *J* = 8.1 Hz, 2H, Ar‐H, H3, and H3′′′), 6.04 (m, 4H, CH, H8, H8′, H8″, and H8′′′), 5.11 (tq, *J* = 16.9, 1.8 Hz, 4H, CH, H_a_9, H_a_9′, H_a_9″, and H_a_9′′′), 4.99 (dddt, *J* = 9.6, 4.8, 2.5, 1.3 Hz, 4H, H_b_9, H_b_9′, H_b_9″, and H_b_9′′′), 3.42 (d, *J* = 6.8 Hz, 4H, CH_2_, H7′, and H7″), and 3.36 (d, *J* = 6.7 Hz, 4H, CH_2_, H7, and H7′′′). ^13^CNMR (125 MHz, (CD_3_)_2_CO, 25°C, TMS, ppm): δ = 152.9 (C2 and C2′′′), 152.1 (C2′ and C2″), 139.1 (CH, C8 and C8′′′), 138.8 (CH, C8′ and C8″), 130.9 (CH, C6 and C6′′′), 130.3 (CH, C6′ and C6″), 130.2 (C5′ and C5″), 130.1 (C5 and C5′′′), 129.3 (C1, C3′ and C3′′′), 128.8 (C1′, C1′′and C1′′′), 127.5 (CH, C4 and C4′′′), 118.2 (CH, C3 and C3′′′), 114.1 (CH_2_, C9′ and C9″), 113.9 (CH_2_, C9 and C9′′′), 39.6 (CH_2_, C7′ and C7″), and 39.4 (CH_2_, C7 and C7′′′). HRMS m/z calcd for C_36_H_34_NaO_4_: 553.2355 [M+Na]^+^, found 553.2370.

### 6.6 5,5′, 5′′, 5′′′‐Tetraallyl‐[1,1′:3′, 1′′:3′′, 1′′′‐Quaterphenyl]−2,4′, 2′′, 2′′′‐Tetraol (6)

Compound **6** was achieved after HPLC‐UV purification (3.1 mg; 3.2%) with 97% purity as a brownish oil. ^1^HNMR (500 MHz, (CD_3_)_2_CO, 25°C, TMS, ppm): *δ* = 7.25 (d, *J* = 2.3 Hz, 2H, Ar‐H, H6″, and H4″), 7.19 (d, *J* = 2.3 Hz, 2H, Ar‐H, H2′, and H6′), 7.13 (d, *J* = 2.3 Hz, 2H, Ar‐H, H6, and H6′′′), 6.99 (dd, *J* = 8.3, 2.3 Hz, 2H, Ar‐H, H4, and H4′′′), 6.80 (d, J = 8.2 Hz, 2H, Ar‐H, H3, and H3′′′), 6.04 (m, 4H, CH, H8, H8′, H8″, and H8′′′), 5.11 (ddq, *J* = 18.6, 16.9, 1.7 Hz, 4H, CH, H_b_9, H_b_9′, H_b_9″, and H_b_ 9′′′), 5.11 (ddq, *J* = 9.9, 6.4, 1.5 Hz, 4H, H_a_9, H_a_9′, H_a_,9″, and H_a_9′′′), 3.42 (d, *J* = 6.7 Hz, 4H, CH_2_, H7′, and H7″), and 3.36 (d, *J* = 6.7 Hz, 4H, CH_2_, H‐7, and H‐7′′′). ^13^CNMR (125 MHz, (CD_3_)_2_CO, 25°C, TMS, ppm): δ = 152.3 (C‐2 and C‐2′′′), 150.6 (C‐4′), 148.9 (C‐2″), 138.4 (CH, C‐8 and C‐8′′′), 138.1 (CH, C‐8′, and C‐8″), 132.2 (C‐5 and C‐5′′′), 131.2 (C‐6 and C‐6′′′), 130.5 (CH, C‐4″, and C‐6″), 130.4 (CH, C‐2′, and C‐6′), 129.7 (CH, C‐4, and C‐4′′′), 128.1 (C‐1′′ and C‐3′′), 128.0 (C‐1′ and C‐1′′′), 127.9 (C‐1′), 127.6 (C‐3′), 116.0 (CH, C‐3, and C‐3′′′), 115.0 (CH_2_, C‐9, and C‐9′′′), 114.8 (CH_2_, C‐9^′,^ and C‐9″), 39.1 (CH_2_, C‐7′, and C‐7″), and 34.6. (CH_2_, C‐7, and C‐7′′′). HRMS m/z calcd for C_36_H_34_NaO_4_: 553.2355 [M+Na]^+^, found 553.2370.

### 6.7 Assay and Kinetic of Metabolic Enzyme Inhibition

The in vitro PL [17], α‐Glu [41], and α‐Amy [41] inhibitory activities of synthesized compounds were evaluated following the methodology previously reported. The details for assay conditions are reported in the Supporting Information. Also, the kinetic experiments are detailed in the Supporting Information.

### 6.8 Molecular Docking Studies

Molecular docking analysis of the monomers on EV4 laccase and the selected compounds on PL, α‐Glu, and α‐Amy was evaluated in silico, following the methodology outlined in our previous studies [17, 41, 44]. Docking studied were performed using the Glide Ligand Docking interfaced with the Maestro (Version 13.7). The details of in silico analysis conditions are reported in the Supporting information. The images were generated using Maestro visualizer, with rendering quality set to custom at 600 DPI and an output of 5976 × 3119 pixels. All images were exported in PNG format, and the relevant visual details including interacting residues and interaction types are described in the corresponding figure captions.

### 6.9 Statistical Analysis

All experimental data were presented as mean ± SD. For each analysis, the sample size was *n* = 3 independent experiments. Regression coefficients were determined using analysis of variance (ANOVA), and statistical significance was determined using a one‐way ANOVA as specified in figure legends. *p* values: *p < 0.05; **p < 0.01; ***p < 0.001 (Turkey's test). Data analysis and graphical presentation were performed using OriginPro 2018 (OriginLab Corporation, Northampton, MA, USA) and JMP Statistical Software (SAS Institute S.r.l., Milan, Italy) was used to the design matrix creation and statistical calculations.

## Supporting Information

Additional supporting information can be found online in the Supporting Information section. The authors have cited additional references within the Supporting Information.[17, 31, 40, 43, 44, 45, 46] **Supporting Fig. S1.** MALDI mass spectrum of the HN dimer (Houpulin B, **4**). **Supporting Fig. S2**. MALDI mass spectrum of the HN dimerization reaction mixture at 2 h. **Supporting Fig. S3**. MALDI mass spectrum of the HN dimerization reaction mixture at 24 h. The insets highlight specific region of the spectrum: HN dimer ( red), trimer (blue) and oligomer (green). **Supporting Fig. S4** *.* Comparison of MALDI mass spectra: blue for Houpulin B, grey for the 2‐hour reaction mixture, and yellow for the 24‐hour reaction mixture. **Supporting Fig. S5** *.* Results of the %yield of honokiol dimers: (A) houpulin A and (B) houpulin B. **Supporting Fig. S6**. Interaction details of monomers **1a** (A), and **2** (B) with EV4 laccase catalytic site. **Supporting Fig. S7**. HRMS [M+Na]^+^ spectrum of **3**. **Supporting Fig. S8**. ^1^HNMR spectrum (500 MHz, (CD_3_)_2_CO) of **3**. **Supporting Fig. S9**.^13^CNMR spectrum (125 MHz, (CD_3_)_2_CO) of **3**. **Supporting Fig. S10**. gCOSY spectrum of **3**. **Supporting Fig. S11**. gHMBC spectrum of **3**. **Supporting Fig. S12**. gHSQC spectrum of **3**. **Supporting Fig. S13**. HRMS [M+Na]^+^ spectrum of **4**. **Supporting Fig. S14**. ^1^HNMR spectrum (500 MHz, (CD_3_)_2_CO) of **4**. **Supporting Fig. S15**. ^13^CNMR spectrum (125 MHz, (CD_3_)_2_CO) of **4**. **Supporting Fig. S16**. gCOSY spectrum of **4**. **Supporting Fig. S17**. gHMBC spectrum of **4**. **Supporting Fig. S18**. gHSQC spectrum of **4**. **Supporting Fig. S19**. HRMS [M+Na]^+^ spectrum of **5**. **Supporting Fig. S20.** ^1^HNMR spectrum (500 MHz, (CD_3_)_2_CO) of **5**. **Supporting Fig. S21**. ^13^CNMR spectrum (125 MHz, (CD_3_)_2_CO) of **5**. **Supporting Fig. S22**. gCOSY of **5**. **Supporting Fig. S23**. gHMBC of **5**. **Supporting Fig. S24**. gHSQC spectrum of **5**. **Supporting Fig. S25**. HRMS [M+Na]^+^ spectrum of **6**. **Supporting Fig. S26**. ^1^HNMR spectrum (500 MHz, (CD_3_)_2_CO) of **6**. **Supporting Fig. S27**. ^13^CNMR spectrum (125 MHz, (CD_3_)_2_CO) of **6**. **Supporting Fig. S28**. gCOSY of **6**. **Supporting Fig. S29**. gHMBC of **6**. **Supporting Fig. S30**. gHSQC of **6**. **Supporting Fig. S31**. PL inhibitory activity of orlistat, compounds **1**, **2** and synthesized dimers **3**–**6**. Data are presented as means ± standard deviation (SD) of *n* = 3 independent experiments. **Supporting Fig. S32**. α‐Glu inhibitory activity of acarbose, compounds **1**, **2** and synthesized dimers **3**–**6**. Data are presented as means ± SD of *n* = 3 independent experiments. **Supporting Fig. S33**. α‐Amy inhibitory activity of acarbose, compounds **1**, **2** and synthesized dimers **3**–**6**. Data are presented as means ± SD of *n* = 3 independent experiments. **Supporting Fig. S34.** L‐B plots showing enzyme inhibition: (A) α‐Glu inhibition in presence of **1**, (B) α‐Amy inhibition in presence of **1** and (C) α‐Amy inhibition in presence of **2.** Data are presented as means ± SD of *n* = 3 independent experiments. **Supporting Fig. S35.** L‐B plots showing enzyme inhibition: (A) PL inhibition in presence of **4, 5** and **6**, (B) α‐Glu inhibition in presence of **4, 5** and **6** and (C) α‐Amy inhibition in presence of **2.** Data are presented as means ± SD of *n* = 3 independent experiments. **Supporting Fig. S36**. The secondary plots obtained from slope (for *K*_i_ determination) of Lineweaver‐Burk plots vs inhibitors concentration: **1** and **2**. Data obtained from (A) α‐Glu and (B) α‐Amy inhibition of HN and (B) α‐Amy inhibition of MG. Data are presented as means ± SD of *n* = 3 independent experiments **Supporting Fig. S37**.The secondary plots obtained from slope (for *K*_i_ determination) of L‐B plots vs inhibitor concentration: **4**. Data obtained from (A) PL, (B) α‐Glu, (C) α‐Amy inhibition. Data are presented as means ± SD of *n* = 3 independent experiments **Supporting Fig. S38.** The secondary plots obtained from slope (for Ki determination) of Lineweaver‐Burk plots vs inhibitor concentration: **5**. (A) PL, (B) α‐Glu, (C) α‐Amy inhibition. Data are presented as means ± SD of *n* = 3 independent experiments. **Supporting Fig. S39.** The secondary plots obtained from slope (for *K*_i_ determination) of Lineweaver‐Burk plots vs inhibitor concentration: **6**. Data obtained from (A) PL, (B) α‐Glu, (C) α‐Amy inhibition. Data are presented as means ± SD of *n* = 3 independent experiments **Supporting Fig. S40**. Compound **5** (plum) into PL (cyan binding site); the interactions are depicted in yellow (hydrogen bonds), cyan (aromatic H‐bond) faded azure (pi‐pi stacking) and green (pi‐cation); only interactive residues are labelled. The color coding used for atoms is red (O), blue (N) and grey (C). **Supporting Fig. S41**. Compound **6** (cyan) into PL (cyan binding site); the interactions are depicted in yellow (hydrogen bonds), cyan (aromatic H‐bond) faded azure (pi‐pi stacking) and green (π‐cation); only interactive residues are labelled. The color coding used for atoms is red (O), blue (N) and grey (C). **Supporting Fig. S42**. Compound **3** (yellow‐green) into PL (cyan binding site); the interactions are depicted in yellow (hydrogen bonds), cyan (aromatic H‐bond) faded azure (pi‐pi stacking) and green (π‐cation); only interactive residues are labelled. The color coding used for atoms is red (O), blue (N) and grey (C). **Supporting Fig. S43**. Compound **5** (plum) into α‐Amy (magenta binding site); the interactions are depicted in yellow (hydrogen bonds), cyan (aromatic H‐bond) faded azure (pi‐pi stacking) and green (π‐cation); only interactive residues are labelled. The color coding used for atoms is red (O), blue (N) and grey (C). **Supporting Fig. S44**. Compound **6** (cyan) into α‐Amy (magenta binding site); the interactions are depicted in yellow (hydrogen bonds), cyan (aromatic H‐bond) faded azure (π‐π stacking) and green (π‐cation); only interactive residues are labelled. The color coding used for atoms is red (O), blue (N) and grey (C). **Supporting Fig. S45**. Compound **3** (yellow‐green) into α‐Amy (magenta binding site); the interactions are depicted in yellow (hydrogen bonds), cyan (aromatic H‐bond) faded azure (π‐π stacking) and green (π‐cation); only interactive residues are labelled. The color coding used for atoms is red (O), blue (N) and grey (C). **Supporting Fig. S46**. Compound **5** (faded blue) into α‐Glu (faded‐orange binding site); the interactions are depicted in yellow (hydrogen bonds), cyan (aromatic H‐bond), faded azure (π‐π stacking) and green (π‐cation); only interactive residues are labelled. The color coding used for atoms is red (O), blue (N) and grey (C). **Supporting Fig. S47**. Compound **6** (cyan) into α‐Glu (faded‐orange binding site); the interactions are depicted in yellow (hydrogen bonds), cyan (aromatic H‐bond) faded azure (π‐π stacking) and green (π‐cation); only interactive residues are labelled. The color coding used for atoms is red (O), blue (N) and grey (C). **Supporting Fig. S48**. Compound **3** (yellow‐green) into α‐Glu (faded‐orange binding site); the interactions are depicted in yellow (hydrogen bonds), cyan (aromatic H‐bond) faded azure (π‐π stacking) and green (π‐cation); only interactive residues are labelled. The color coding used for atoms is red (O), blue (N) and grey (C). **Supporting Fig. S49**. Molecular surface of PL (cyan), α‐Amy (faded orange), α‐Glu (magenta) with **3–6** into the BS. **Supporting Fig. S50**. PL binding site and overlap of: (A) **3**–**6**; (B) **3** and **4**; (C) **4** and **6**. **Supporting Fig. S51.** 1OSE detail on the surface of (A) **4**; (B) **1**; (C) **1** and **4**. **Supporting Fig. S52.** 1OSE detail of α‐amylase catalytic triad and (A) **4** and (B) hypoglycaemic drug acarbose. **Supporting Fig. S53.** Interaction details of dimer (A) **3**, (B) **4**, (C) **5** and (D) **6** and α‐Amy catalytic site. **Supporting Fig. S54**. (A) α‐Amy–overlap 1OSE dimer **4** and **5**; B) 1OSE detail interaction. **Supporting Fig. S55.** Interaction details of dimer A) **3**, B) **4**, C) **5** and D) **6** and α‐Glu catalytic site. **Supporting Fig. S56.** Detail of Asp 349 interaction in the deepest portion of the hydrophobic pocket and comparison with dimer **4**. **Supporting Fig. S57**. α‐Glu ‐overlap of **5** and **6**. **Supporting Table S1**: Preliminary screening conditions evaluated for LTV mediated synthesis of honokiol dimers in organic solvent. **Supporting Table S2**: Reaction conditions evaluated for laccase mediated synthesis of honokiol dimers. **Supporting Table S3**. Conditions applied in the single factor experiments for the optimization of dimerization reaction conditions of honokiol mediated by EV4. **Supporting Table S4.** Preliminary ranges and coded levels of the selected independent variables. **Supporting Table S5**. Experimental design and result of the %yield of honokiol dimers. **Supporting Table S6**. Results of ANOVA from the BBD model for %yield of **3** (*Y*_1_) and **4** (*Y*_2_). **Supporting Table S7.** Comparison between actual‐by‐predicted values of dimerization reaction obtained at optimal conditions. **Supporting Table S8**. Binding energies (*ΔG*_bind_) and interacting residues of ligands (**1–6**) with catalytic site.^a^ **Supporting Table S9**. List of molecular interactions of **1** – **6** with PL Catalytic Site. **Supporting Table S10**. List of molecular interactions of **1** – **6** with α‐Amy Catalytic Site. **Supporting Table S11**. List of molecular interactions of **1**–**6** with α‐Glu Catalytic Site.

## Conflicts of Interest

The authors declare no conflicts of interest.

## Supporting information

Supplementary Material
