# Supplementary material for: Laccase‐Catalyzed Dimerization of Honokiol and Magnolol for Multitarget Metabolic Enzyme Inhibitors
Source: Chembiochem. 2026 Apr 4;27(7):e70287. doi: 10.1002/cbic.70287 (PMC13050280; doi:10.1002/cbic.70287)
Supplement: Supplementary file 1 — Supplementary Material [file CBIC-27-e70287-s001.pdf]

Supporting information

Laccase-Catalyzed Dimerization of Honokiol and Magnolol for Multitarget Metabolic Enzyme Inhibitors

Claudia Sciacca,[a] Nunzio Cardullo,[a] Luana Pulvirenti,[b] Simona Varriale,[c] Libera Vitiello,[d] Cinzia Pezzella[c] and Vera Muccilli*[a,d]

[a]Department of Chemical Sciences, University of Catania, V.le A. Doria 6, 95125 Catania (Italy)
[b]Institute of Biomolecular Chemistry, National Research Council ICB-CNR, Via Paolo Gaifami 18, 95126 Catania (Italy)
[c]Department of Chemical Sciences, University of Naples Federico II, Via Cinthia 4, 80126 Napoli (Italy)
[d]Institute for Polymers, Composites and Biomaterials, National Research Council IPCB-CNR, Via Paolo Gaifami 18, 95126 Catania (Italy)

Corresponding author: vera.muccilli@unict.it

Materials and methods

Material

All chemicals were of reagent grade, purchased from Sigma-Aldrich (Milan, Italy) and TCI Europe N.V. (Zwijndrecht, Belgium) and employed without any further purification. Laccase from *Trametes versicolor* (light brown powder, 1.4 units/mg protein), α-glucosidase (α-Glu) from *Saccharomyces cerevisiae* (type I, lyophilized powder, 10 units/mg protein), α-amylase (α-Amy) from porcine pancreas (type VI, lyophilized powder, 10 units/mg protein), lipase (PL) from porcine pancreas (type II, lyophilized powder, 125 units/mg protein), acarbose, orlistat, 4-nitrophenyl-α-D-glucopyranoside,4-nitrophenylbutyrate, 2-chloro-4-nitrophenyl-α-D-maltotrioside were purchased from Sigma-Aldrich (Milan, Italy). Magnolol and honokiol were purchased from TCI Europe N.V. (Zwijndrecht, Belgium). Laccases from *Pleurotus ostreatus* POXA1b and its two evolved variants (EV4 and EV3) were recombinantly expressed in *Pichia pastoris* as previously described in [44]. All reactions were monitored by HPLC-UV analysis, using an Agilent 1100 Series (Milan, Italy). The reaction yield was quantified on an analytical reversed-phase column (RP-18; 4.6 × 250 mm, 5 μm) with the following gradient of CH_3CN/H^+ (99:1 v/v; B) in H_2O/H^+ (99:1 v/v; A) at 1 mL/min: t0 min B = 40%, t15 min B = 100%, t25 min B = 40% measured at 280 nm. Dimer's purification was conducted using a semi-preparative reversed-phase column (RP-18; 10 × 250 mm, 10 μm) with isocratic elution CH_3CN/H_2O (84:16 v/c) at 5 mL/min. NMR spectra were recorded on a Varian Unity Inova spectrometer at 500 MHz (^1H) and 125 MHz (^{13}C) in 5mm NMR tubes, using software supplied by the manufacturer and acquired at 300 K. Samples were dissolved in $(CD_3)_2CO$, with chemical shifts (δ) referenced to TMS using solvent residual signals δ 2.05. High-resolution mass spectra were acquired with a Q Exactive Orbitrap mass spectrometer (Thermo Fisher Scientific, Bremen, Germany) equipped with an E.S.I. ion source operating in positive or in negative mode. Samples were dissolved at 1E- 5 M concentration in 50:50 ($MeOH/H_2O$ +1% formic acid) and directly infused in the mass spectrometer. MALDI-TOF mass spectra were obtained in linear mode using a Bruker Ultraflex Extreme MALDI TOF/TOF instrument (Bruker Daltonics, Billerica, MA, USA). Analyses were carried out at 20 kV acceleration voltage and a 250 ns delay time, collecting 1000 laser shots for each sample. To minimize interference from matrix-related signals, ion deflection was applied up to an m/z value of 2000. Prior to analysis, samples were dissolved in chloroform ($CHCl_3$) at a concentration of 10 mg/mL and subsequently mixed with a dithranol (DT) matrix solution prepared in chloroform (CHCl3, 0.1 M), using a matrix-to-analyte weight ratio of 1:1. The metabolic enzyme inhibition assays were carried out using a Synergy H1 microplate reader.

Preliminary dimerization reaction of honokiol

Preliminary experiments for laccase-mediated dimerization reaction were performed employing honokiol (HN, **1**) (5 mg/3mL) as substrate. The reaction was catalysed using different laccases enzymes, including those from *Trametes versicolor* (LTV), a recombinant laccase from *Pleurotus ostreatus* POXA1b and its two evolved variants (EV4 and EV3). Reactions were carried out in 0.1 phosphate, pH 6.0 alone or in combination with organic solvent (organic solvent: aqueous 1:5, **Table S1**). The addition of the following chemical mediators*.* 2,2'-azino-bis (3-ethylbenzothiazoline-6-sulfonic acid (ABTS, 2.93 mg, 0.3 equiv) was explored. All reactions were carried out at room temperature and monitored over

time by HPLC-UV analysis. Enzyme amount was set to 12 U, taking into consideration the specific activities of each enzyme preparation: LTV (1.4 U/mg); POXA1b (17 U/mg; EV3 4 U/mg; EV4 17 U/mg). The specific reaction conditions are detailed in **Table S2**. The reaction yields were quantified by HPLC-UV analysis using an analytical reverse phase column (RP-18; 4.6 × 250 mm, 5 μm) under the following CH_3CN/H^+ (99:1 v/v; B) in H_2O/H^+ (99:1 v/v; A) gradient: t0 min B = 40%, t15 min B = 100%, t25 min B = 40%, with a flow rate of 1 mL/min. Compounds were purified by HPLC-UV using a semi-preparative column under the following CH_3CN/ H_2O (86:14 v/v) in isocratic with a flow rate of 5 mL/min.

Table S1: Preliminary screening conditions evaluated for LTV mediated synthesis of honokiol dimers in organic solvent.

Entry	Enzyme	Time (h)	Solvent	Mediator	3 %yield	4 %yield
1	LTV	96	dioxane	-	0.7	0.8
2	LTV	96	ethyl acetate	-	0	0
3	LTV	96	dioxane	ABTS	0.07	0.07
4	LTV	96	ethyl acetate	ABTS	0.04	0.06

Table S2*:* Reaction conditions evaluated for laccase mediated synthesis of honokiol dimers.

Entry	Enzyme	Time (h)	Mediator	3 %yield	4 %yield
1	LTV	5	-	0	0
2	LTV	96	-	1.2	2.3
3	LTV	120	-	0	0
4	LTV	2	ABTS	0	0
5	LTV	4	ABTS	0	0
6	LTV	8	ABTS	0	0
7	LTV	24	ABTS	0	0
8	LTV	48	ABTS	0	0
9	LTV	96	ABTS	0.7	0.9

10	LTV	120	ABTS	0	0
11	LTV*	48	-	0.6	0.65
12	LTV*	96	-	0.6	0.72
13	LTV*	120	-	0	0
14	LTV*	48	ABTS	0.8	1.0
15	LTV*	96	ABTS	1.1	1.5
16	LTV*	120	ABTS	0	0
17	POXA1B	5	-	0.9	1.9
18	POXA1B	96	-	0.8	1.6
19	POXA1B	2	ABTS	2.2	2.4
20	POXA1B	4	ABTS	1.5	2.1
21	POXA1B	8	ABTS	0.8	1.6
22	POXA1B	24	ABTS	0	0.4
23	POXA1B	96	ABTS	0	0
24	EV4	5	-	1.0	2.6
25	EV4	96	-	0.5	1.1
26	EV4	2	ABTS	3.0	3.0
27	EV4	4	ABTS	0.8	2.2
28	EV4	8	ABTS	0	2.1

29	EV4	24	ABTS	0	1
30	EV4	96	ABTS	0	0
31	EV4**	3	-	2.6	2.6
32	EV3	5	-	1.1	2.3
33	EV3	96	-	0.4	1.0
34	EV3	2	ABTS	2.3	2.7
35	EV3	4	ABTS	0.5	2.1
36	EV3	8	ABTS	0	1.6
37	EV3	24	ABTS	0	0.5
38	EV3	96	ABTS	0	0

*Double enzyme amount

**Sequential addition of the enzyme over the time.

MALDI mass spectrometry analyses

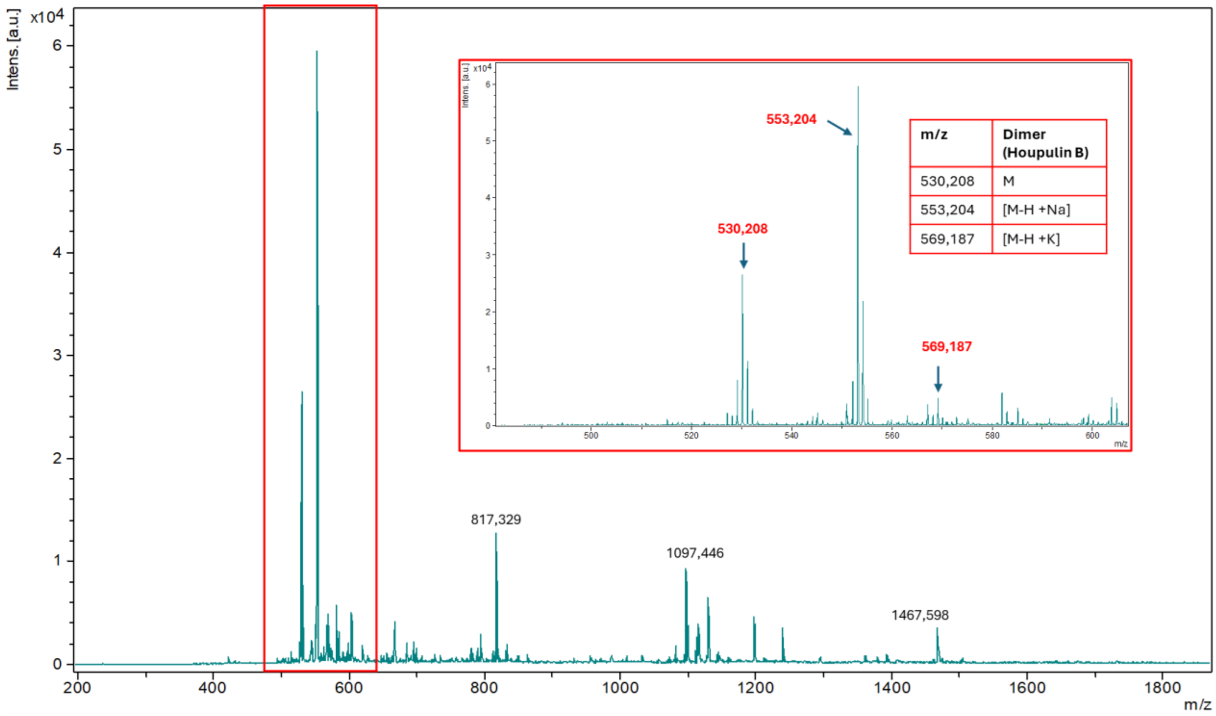

Figure S1. MALDI mass spectrum of the HN dimer (Houpulin B, **4**).

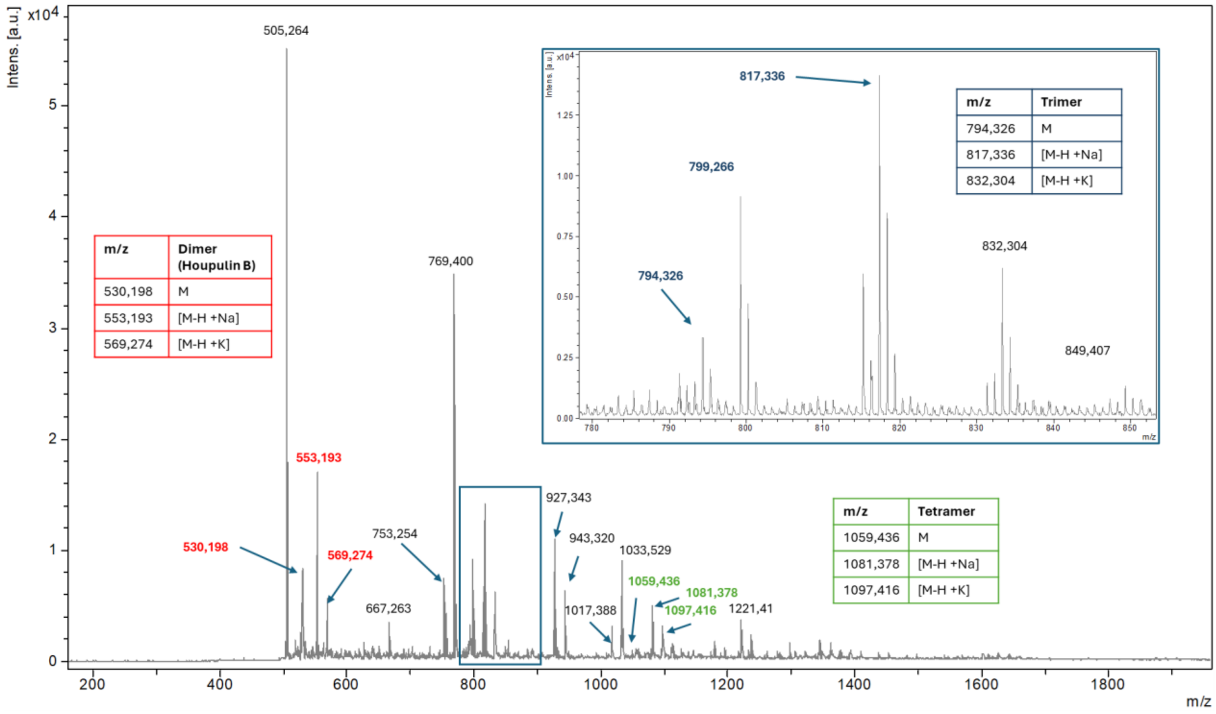

Figure S2. MALDI mass spectrum of the HN dimerization reaction mixture at 2 hours.

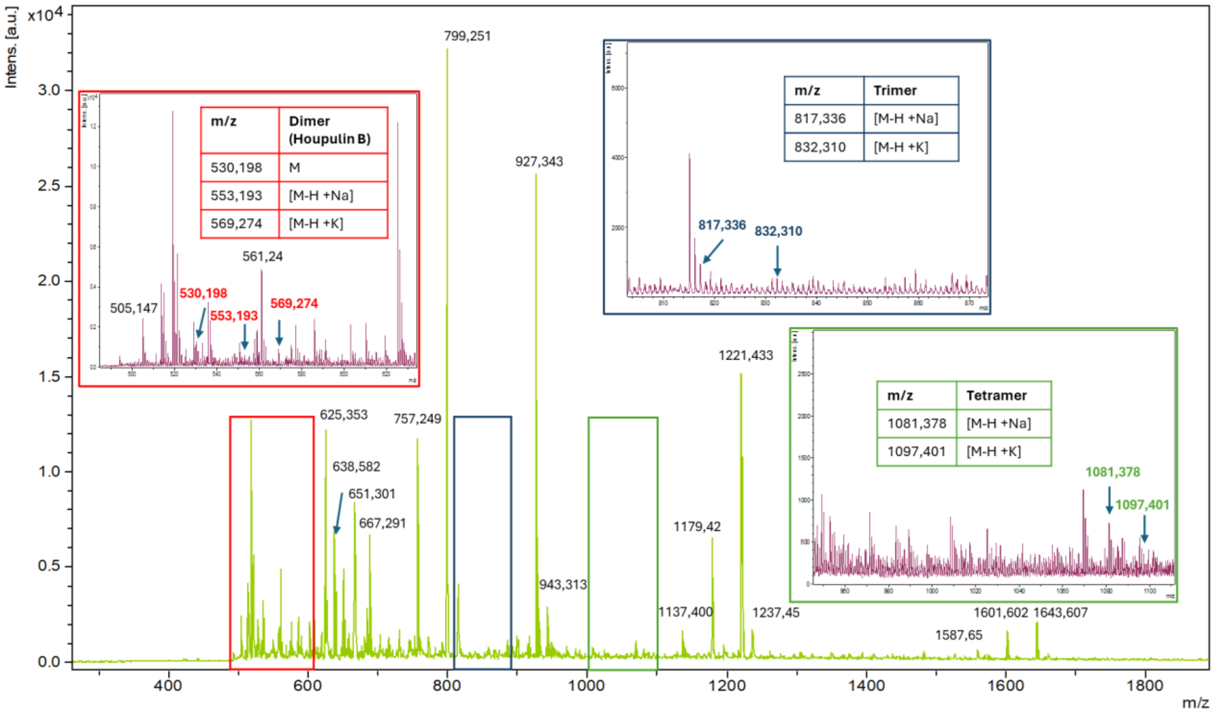

Figure S3. MALDI mass spectrum of the HN dimerization reaction mixture at 24 hours. The insets highlight specific region of the spectrum: HN dimer (red), trimer (blue) and oligomer (green).

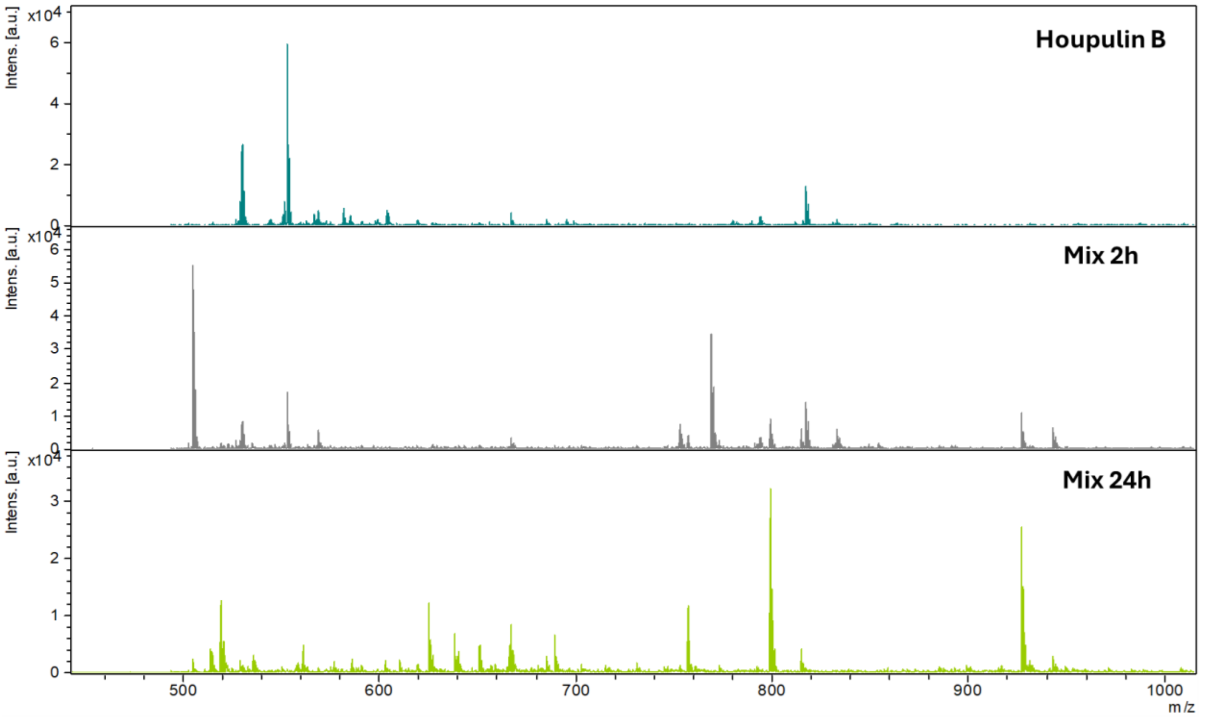

Figure S4. Comparison of MALDI mass spectra: blue for Houpulin B, grey for the 2-hour reaction mixture, and yellow for the 24-hour reaction mixture.

Box-Behnken experimental design (BBD)

Table S3. Conditions applied in the single factor experiments for the optimization of dimerization reaction conditions of honokiol mediated by EV4.

Variables	Factors	Levels
X_1	Buffer pH	4, 5, 6*, 7
X_2	Reaction time (min)	15, 30, 60, 120*, 240, 480
X_3	Honokiol concentration (mg/mL)	0.33, 0.83, 1.67*, 2.5, 3.33

*Fixed values.

Table S4. Preliminary ranges and coded levels of the selected independent variables.

Independent variables	Code	Coded levels		
		**-1**	**0**	**+1**
pH	X_1	5	6	7
Reaction Time (min)	X_2	60	120	240
Substrate concentration (mg/mL)	X_3	0.33	0.83	1.67

Table S5. Experimental design and result of the %yield of honokiol dimers.

Run	Independent variables			Dependent variables	
	Reaction conditions			Experimental values	
	X_1 pH	X_2 (Time, min)	X_3 (Concentration, mg/mL)	Y_1 (%yield)	Y_2 (%yield)
1	6	120	0.83	2.35 ± 0.1	2.35 ± 0.1
2	5	60	0.33	1.45 ± 0.04	1.46 ± 0.04
3	7	120	0.83	2.95 ± 0.18	2.95 ± 0.18
4	7	120	0.33	3.1 ± 0.19	3.1 ± 0.19
5	6	240	0.33	2.01 ± 0.07	2.02 ± 0.06
6	6	120	0.83	2.34 ± 0.1	2.34 ± 0.1
7	7	120	1.67	2.23 ± 0.09	2.22 ± 0.09
8	5	60	0.33	1.75 ± 0.04	1.77 ± 0.03
9	5	120	1.67	0.88 ± 0.02	0.88 ± 0.02
10	7	60	0.83	2.46 ± 0.11	2.46 ± 0.11
11	5	240	0.83	1.07 ± 0.06	1.08 ± 0.06
12	6	120	0.83	2.31 ± 0.09	2.31 ± 0.09
13	6	60	1.67	0.91 ± 0.02	0.91 ± 0.02
14	6	60	0.33	2.13 ± 0.05	2.15 ± 0.05
15	6	240	1.67	2.1 ± 0.07	2.1 ± 0.07

Data are reported as means ± SD (n = 3).

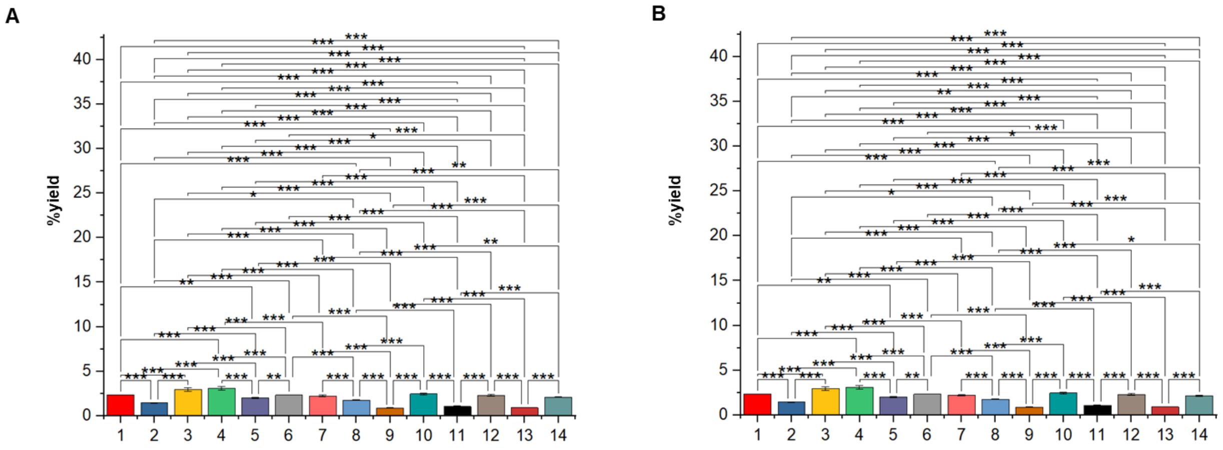

Figure S5. Results of the %yield of honokiol dimers: (A) houpulin A and (A) houpulin .

Table S6. Results of ANOVA from the BBD model for %yield of **3** (Y_1) and **4** (Y_2).

Variable	Sum of squares		*F*-value		*p*-value	
	Y_1	Y_2	Y_1	Y_2	Y_1	Y_2
Model	6.35	6.38	126.98	133.13	<0.0001*	<0.0001*
X_1	2.26	2.29	405.17	430.96	<0.0001*	<0.0001*
X_2	0.28	0.28	50.31	53.34	0.0009*	0.0008*
X_3	0.72	0.68	128.93	128.51	<0.0001*	<0.0001*
$X_1^* X_2$	0.03	0.03	4.76	5.45	0.0809	0.0668
$X_1^* X_3$	0.001	0.0011	0.18	0.21	0.6930	0.6680
$X_2^* X_3$	0.49	0.48	87.90	90.63	0.0002*	0.0002*
$X_1^* X_1$	0.02	0.02	3.68	3.45	0.1133	0.1222
$X_2^* X_2$	0.31	0.29	53.15	55.25	0.0008*	0.0007*
$X_3^* X_3$	0.04	0.04	7.40	8.13	0.0418*	0.0344*
Lack of fit	0.03	0.03	15.24	18.05	0.0625	0.0632
R^2	0.995	0.995				

R^2(Adj.)	0.989	0.989

[*]Values statistically significant at $p<0.05$.

Table S7. Comparison between actual-by-predicted values of dimerization reaction obtained at optimal conditions.

Responses	3<br>%yield	4<br>%yield
**Measured**	3.42 ± 0.039[a]	3.42 ± 0.032[a]
**Predicted**	3.20[a]<br>(2.95-3.46)	3.20[a]<br>(2.95-3.46)

Data are reported as means ± standard deviations and different letters in the same column indicate significant differences (Turkey's test).

Computational analysis of laccase – neolignans interactions

The model of POXA1b was used to build the three-dimensional structures of laccase variants by swapping the residues involved in the mutations as previously described [35]. Receptor was prepared with Protein wizard and the molecular docking studies were performed using Glide Ligand Docking interfaced with Maestro and Autodock Vina software 1.5.6. The grid box was centered in the binding site of protein, with grid centre set to –3.67 (x), 0.9 (y) and 12.75 (z). During the docking process, a maximum of 5 conformers was considered for each ligand. In Docking calculation, ligands were treated as flexible while protein was treated as rigid. The analysis of docking outcomes was carried out by Maestro (Version 13.7).

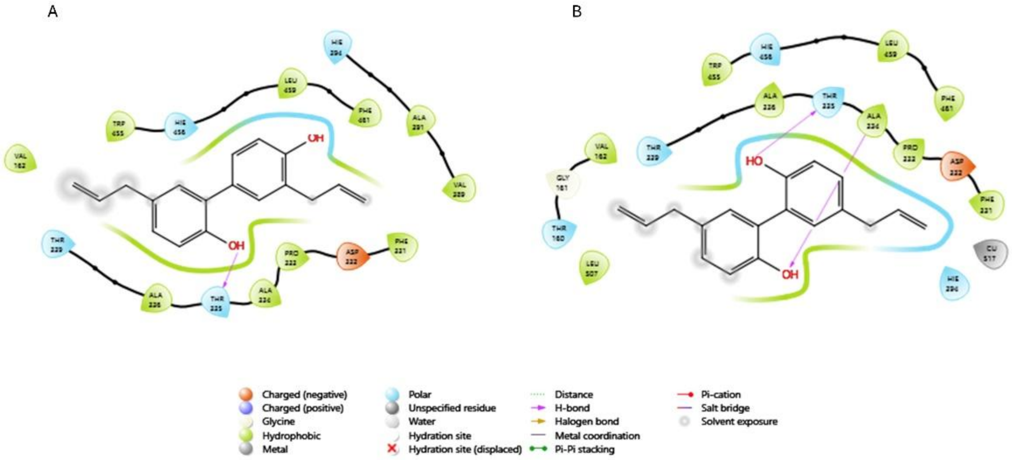

Figure S6. Interaction details of monomers 1a (A), and 2 (B) with EV4 laccase catalytic site.

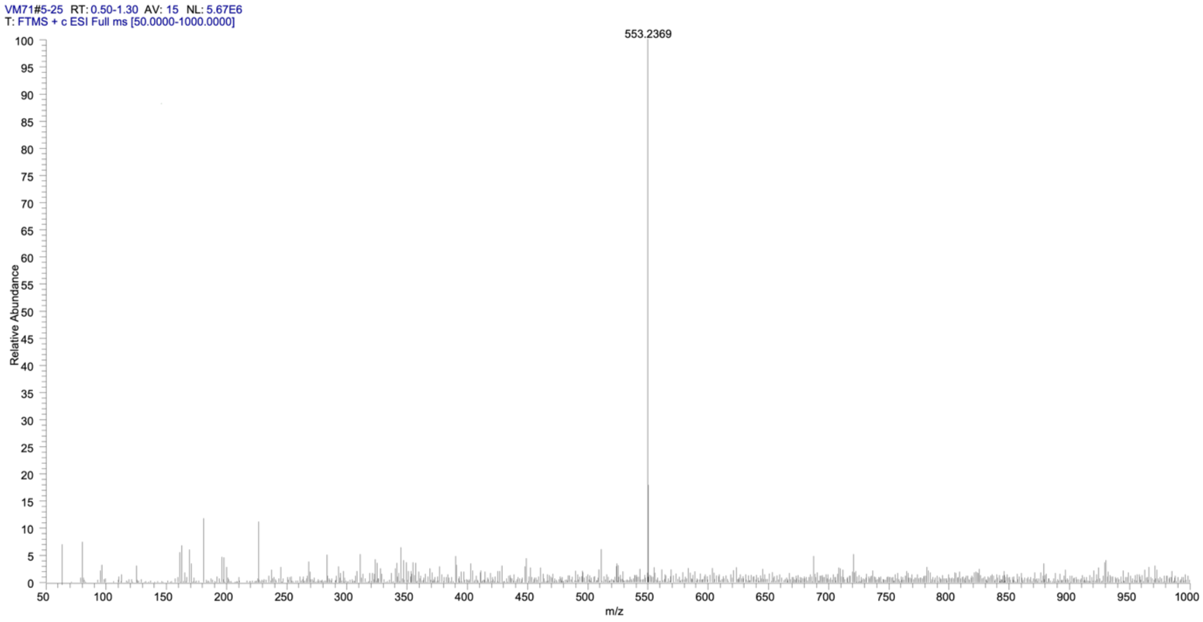

Figure S7. HRMS $[M+Na]^+$ spectrum of **3**.

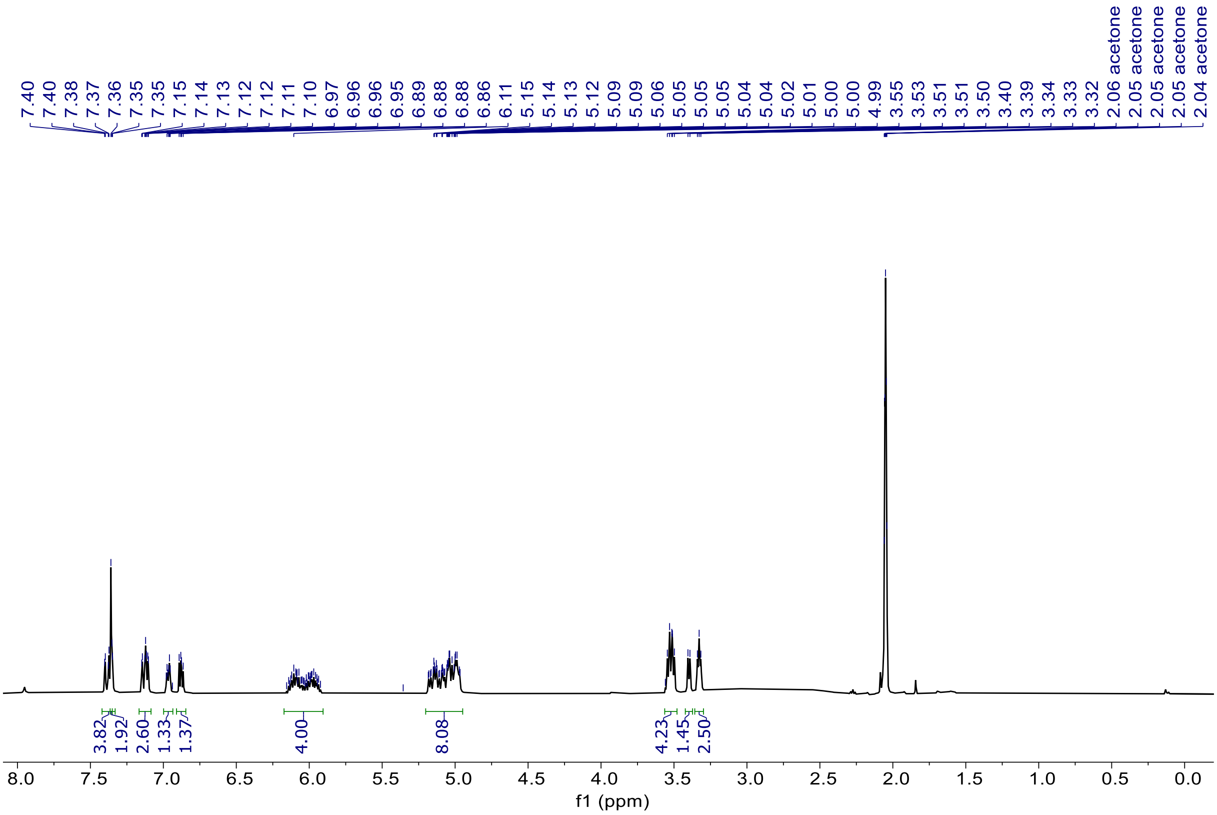

Figure S8. ^{1}HNMR spectrum (500 MHz, $(CD_3)_2CO$) of **3**.

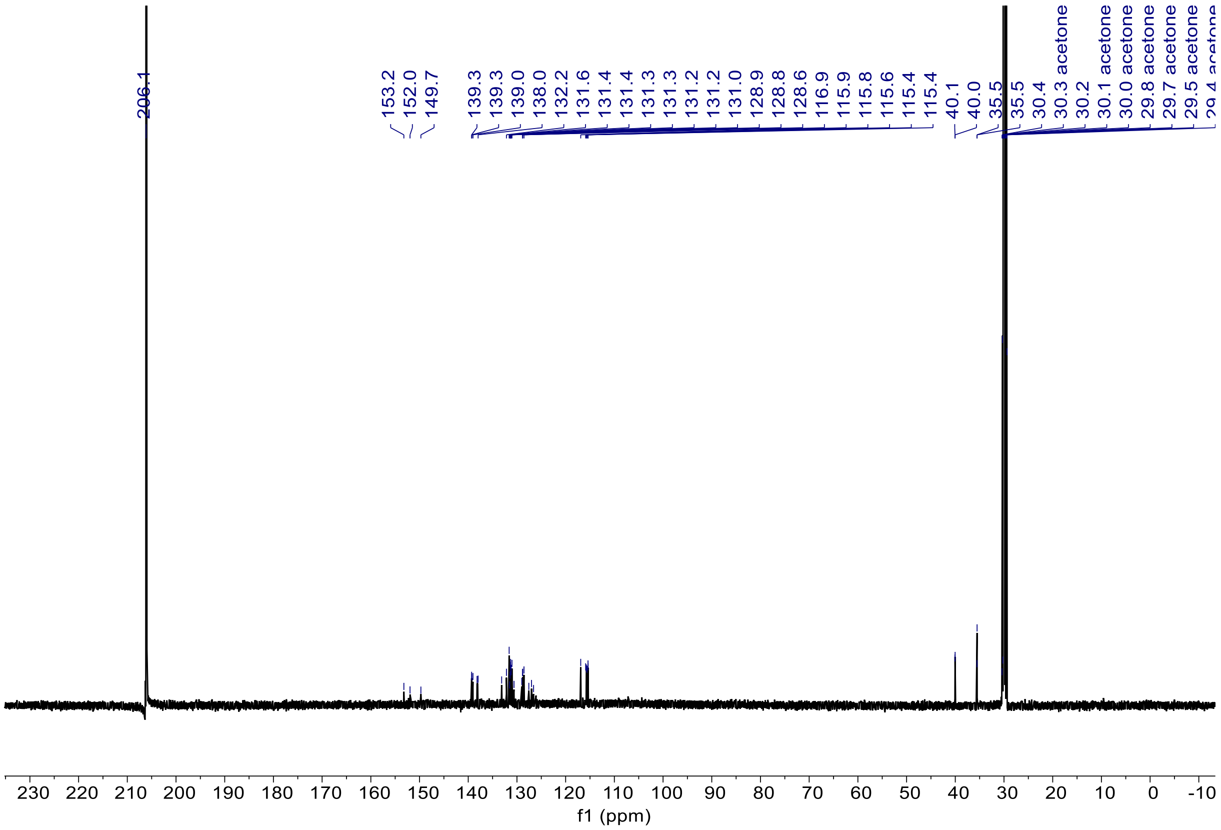

Figure S9.^{13}CNMR spectrum (125 MHz, $(CD_3)_2CO$) of **3**.

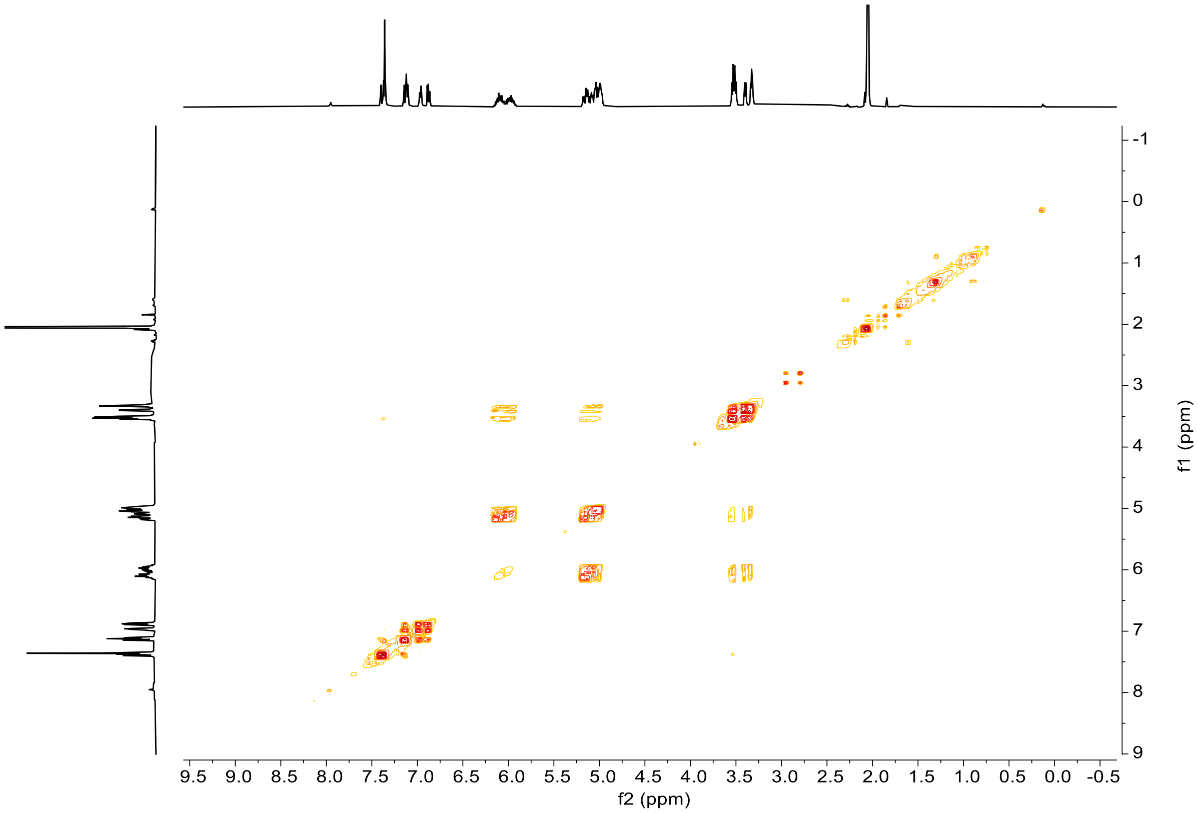

Figure S10. gCOSY spectrum of **3**.

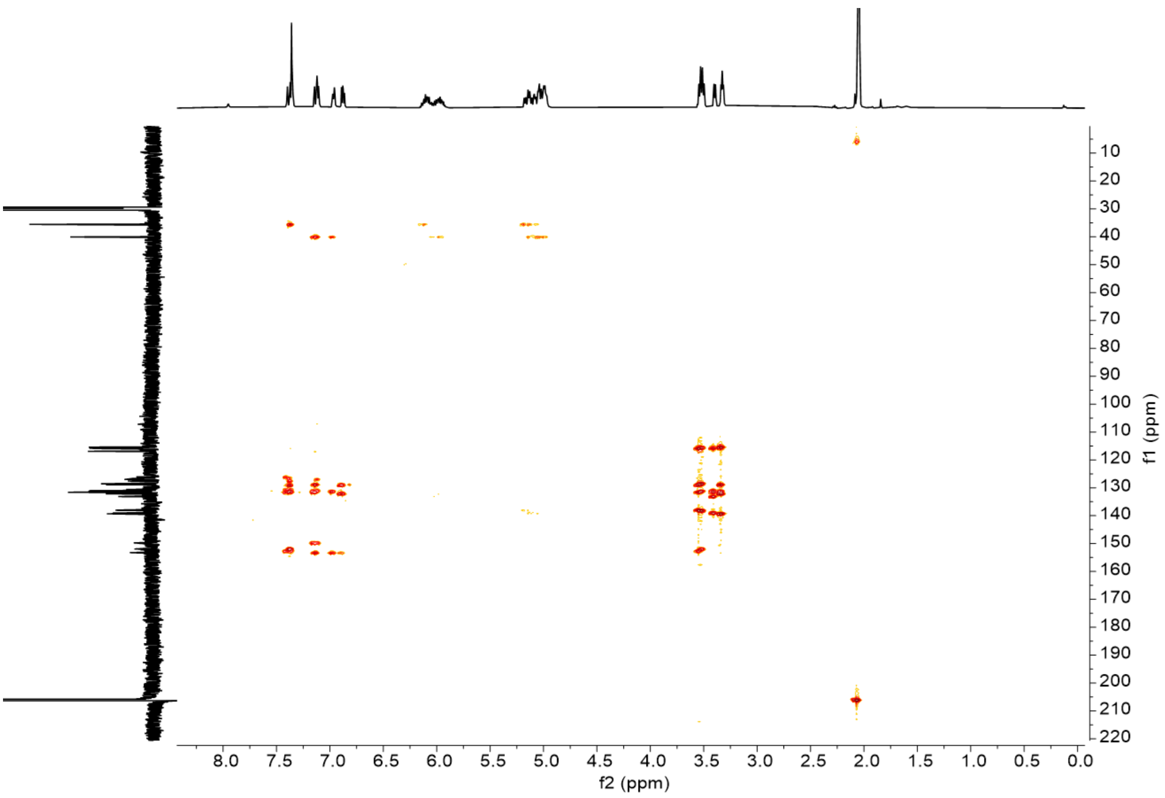

Figure S11. gHMBC spectrum of **3**.

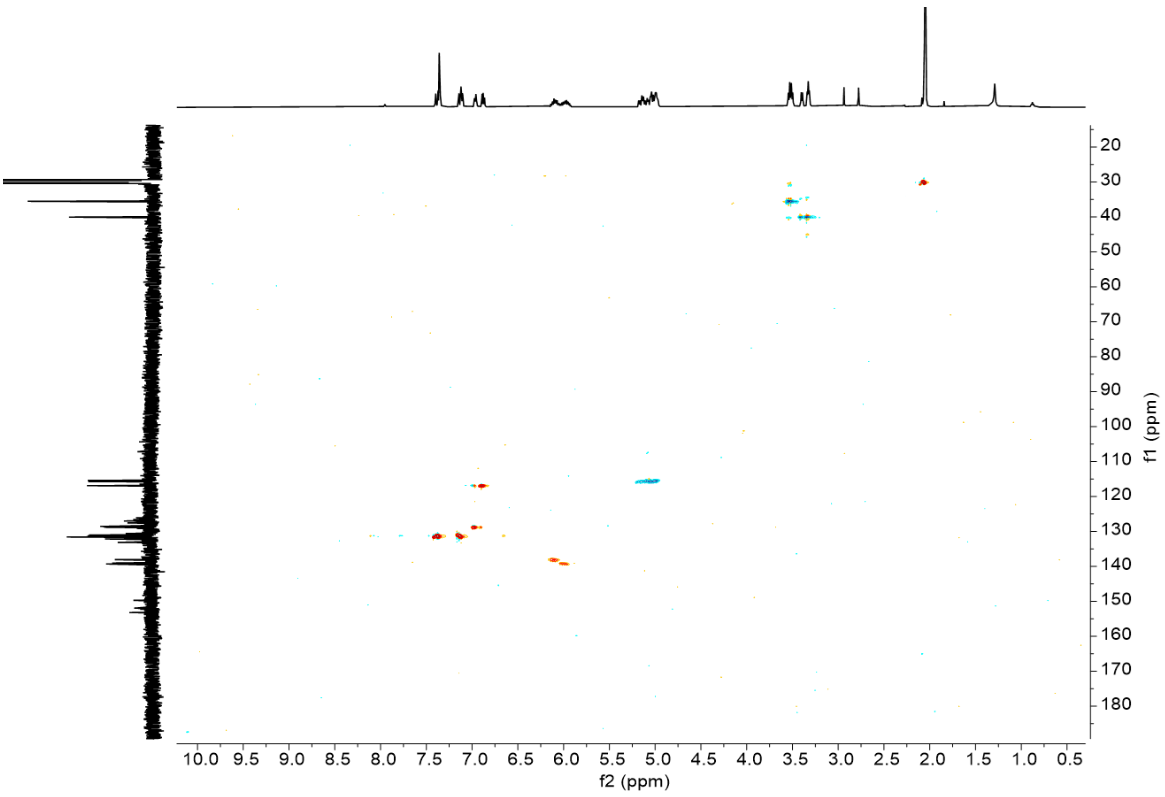

Figure S12. gHSQC spectrum of **3**.

.

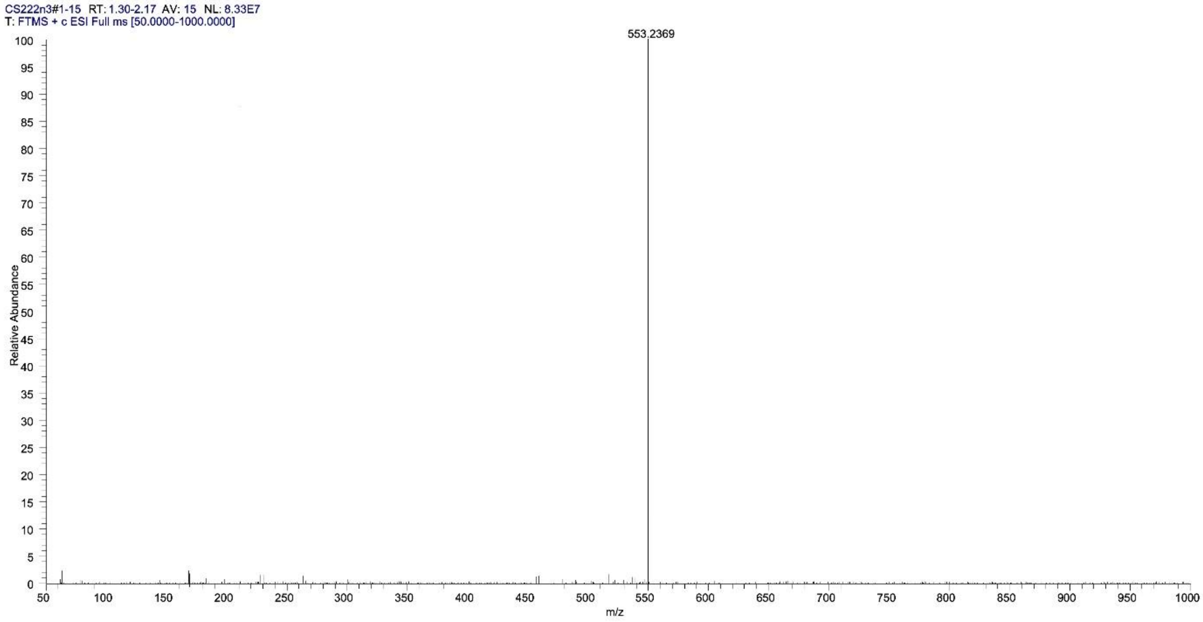

Figure S13. HRMS $[M+Na]^+$ spectrum of **4**.

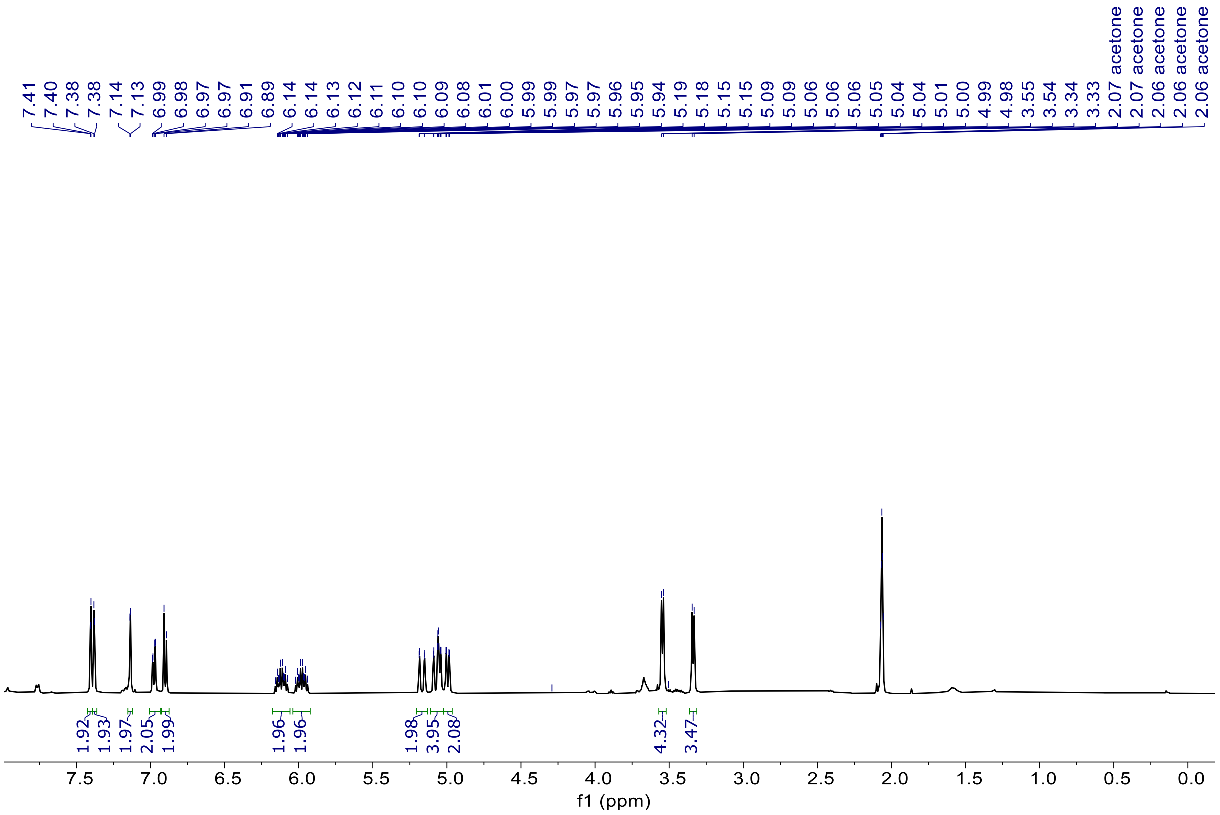

Figure S14. ^{1}HNMR spectrum (500 MHz, $(CD_3)_2CO$) of **4**.

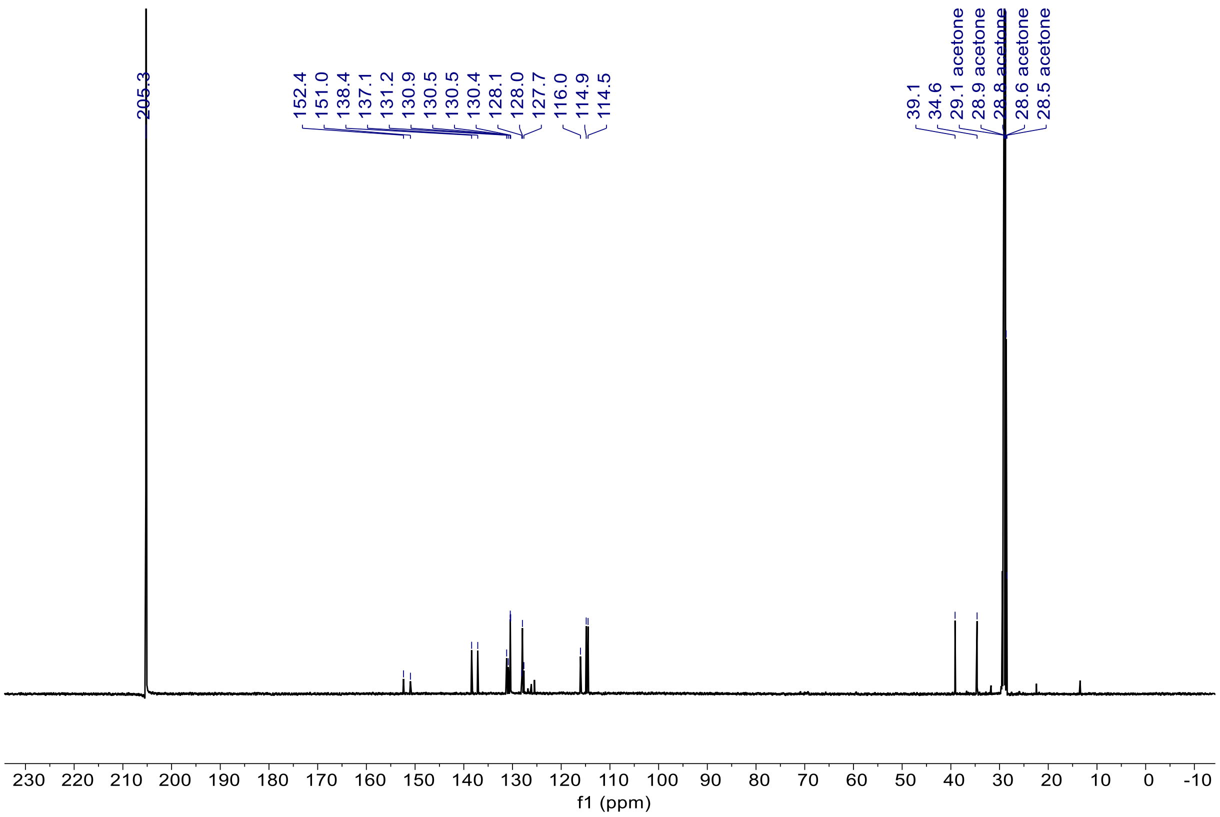

Figure S15. ^{13}CNMR spectrum (125 MHz, $(CD_3)_2CO$) of **4**.

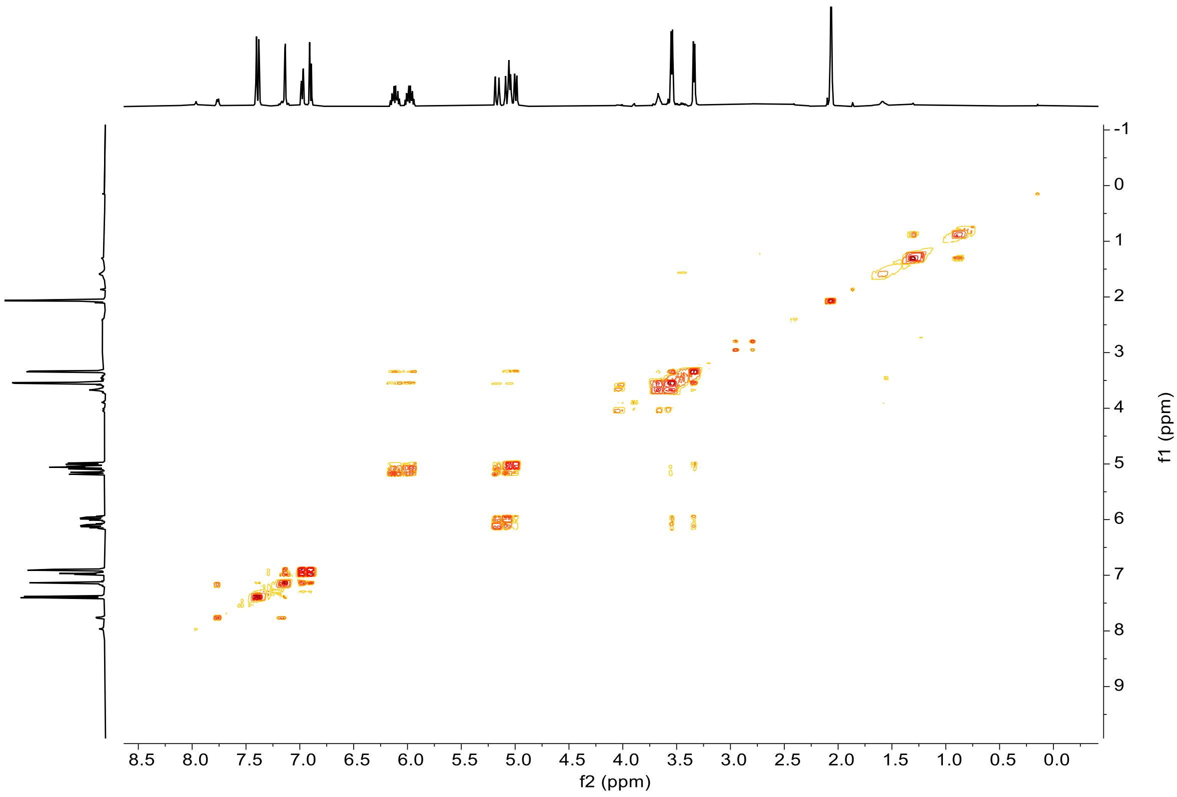

Figure S16. gCOSY spectrum of **4**.

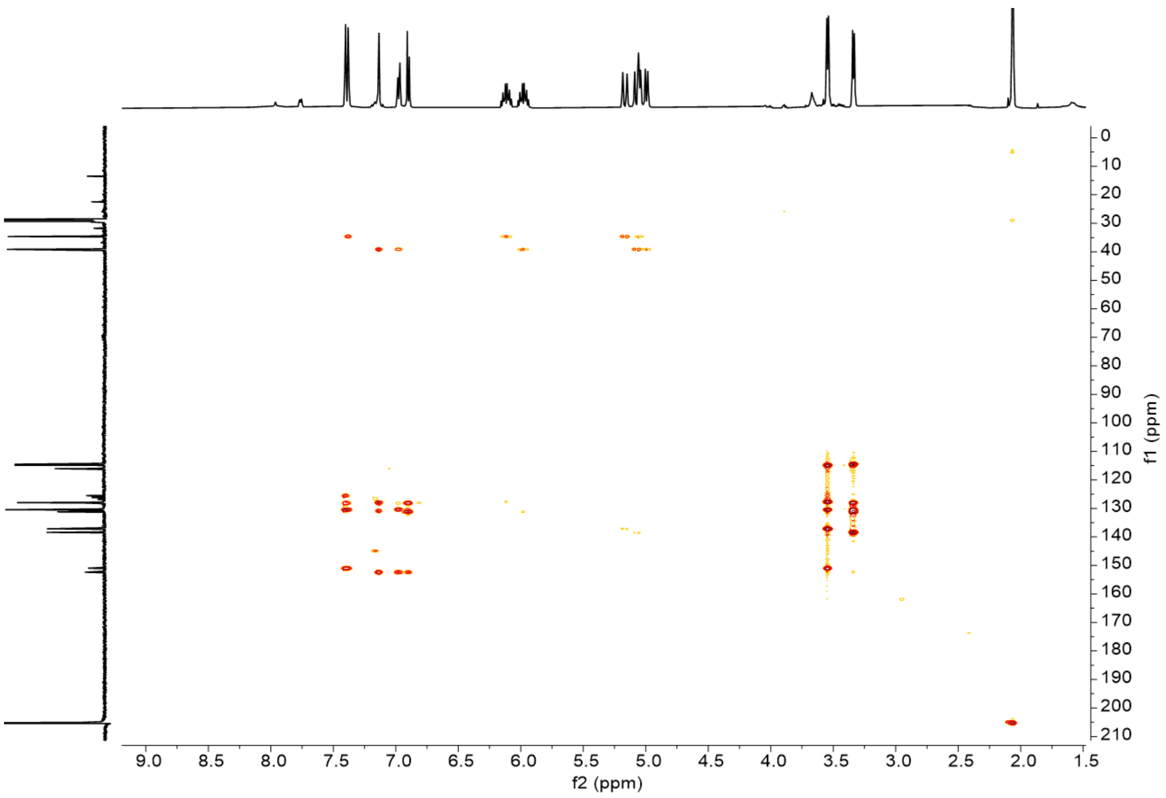

Figure S17. gHMBC spectrum of **4**.

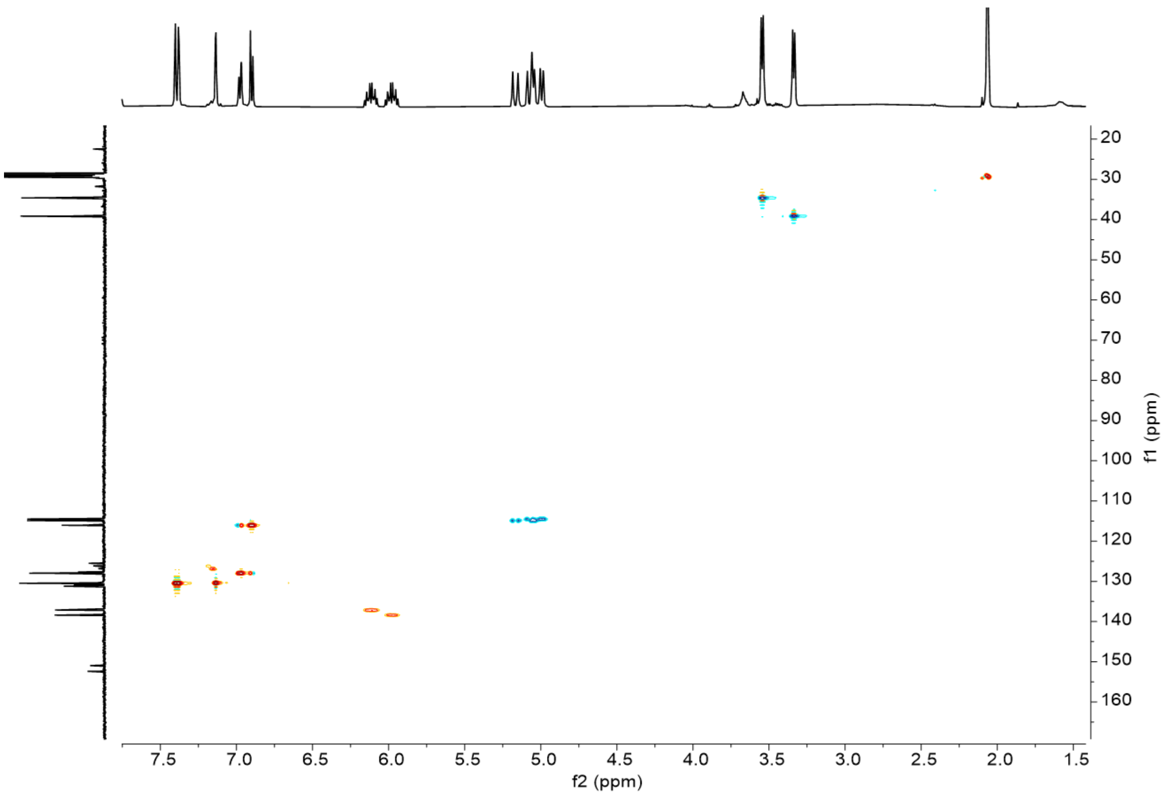

Figure S18. gHSQC spectrum of **4**.

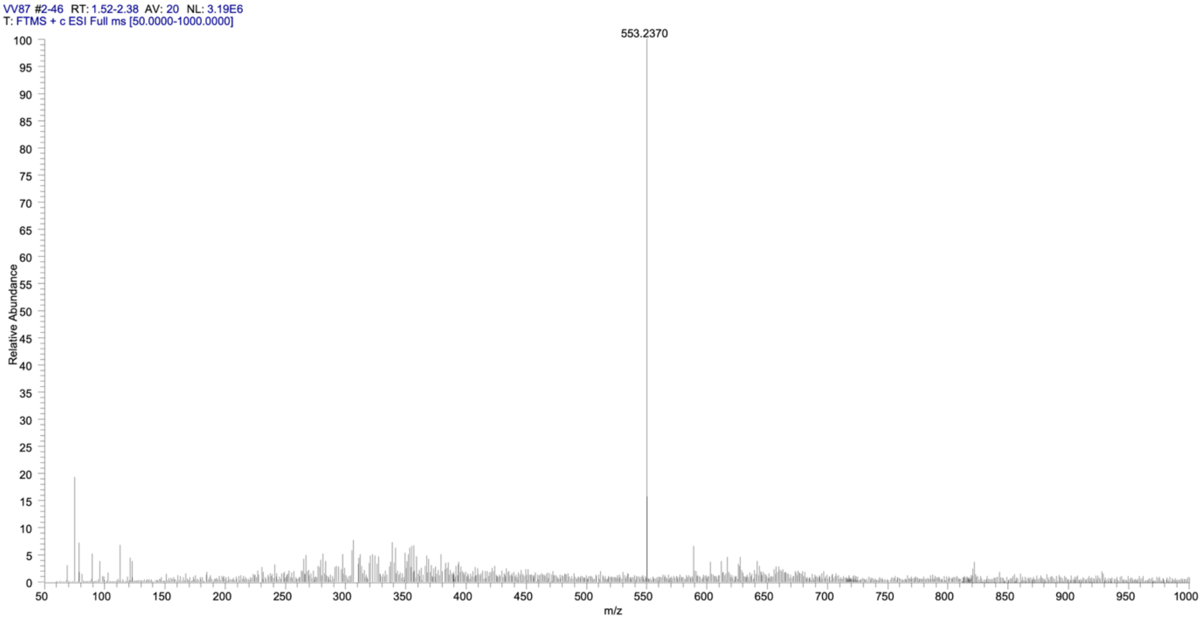

Figure S19. HRMS [M+Na]+ spectrum of **5**.

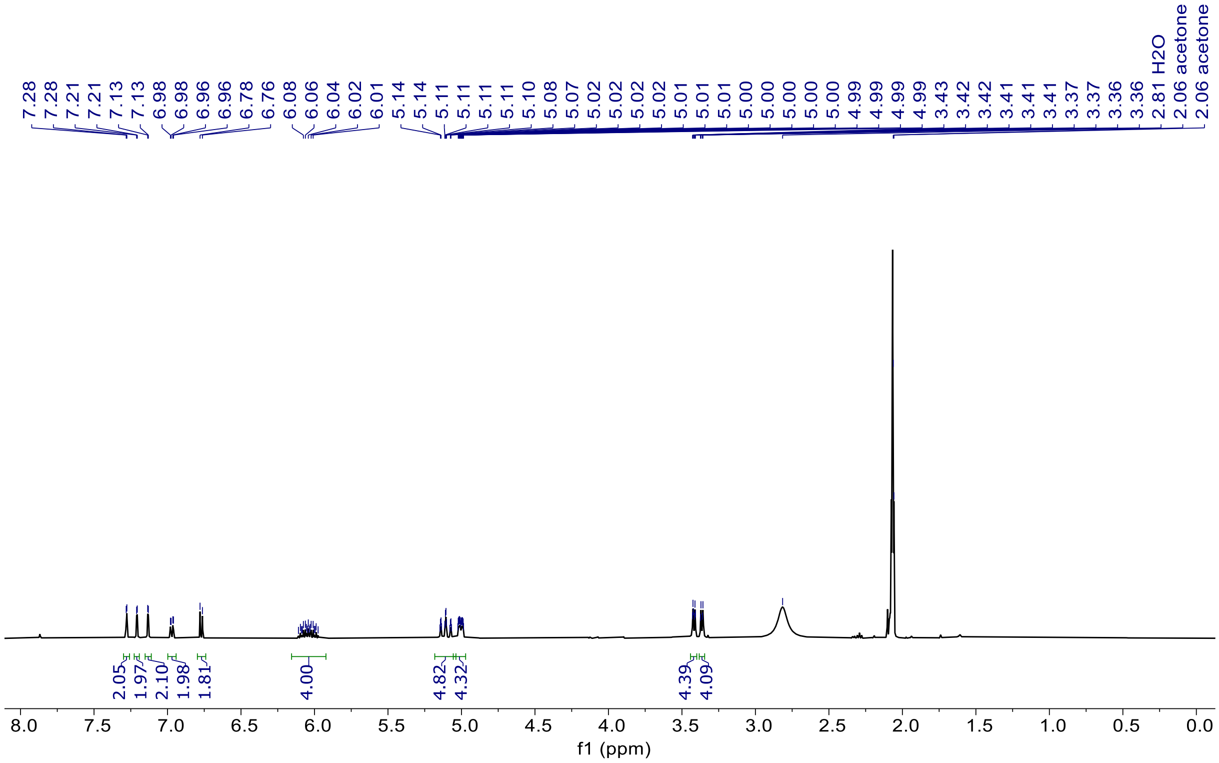

Figure S20. ^{1}HNMR spectrum (500 MHz, $(CD_3)_2CO$) of **5**.

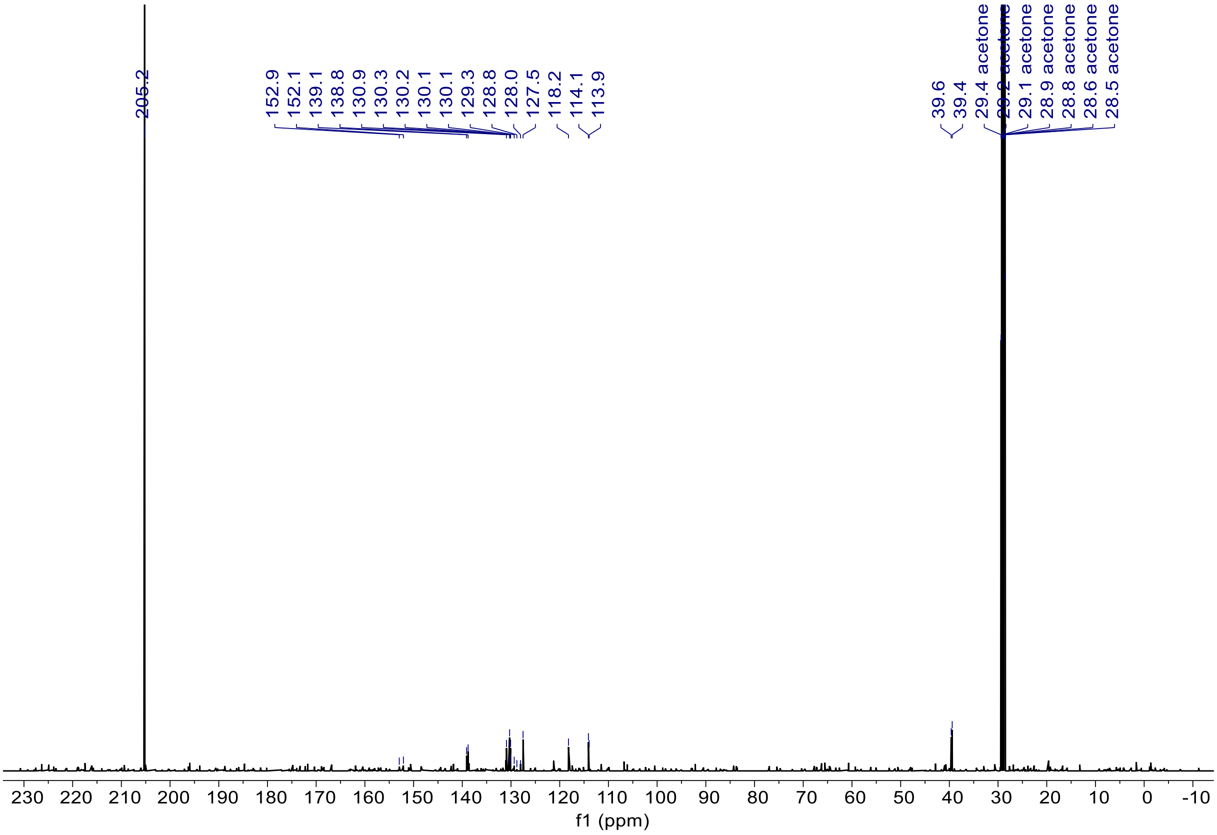

Figure S21. ^{13}CNMR spectrum (125 MHz, $(CD_3)_2CO$) of **5**.

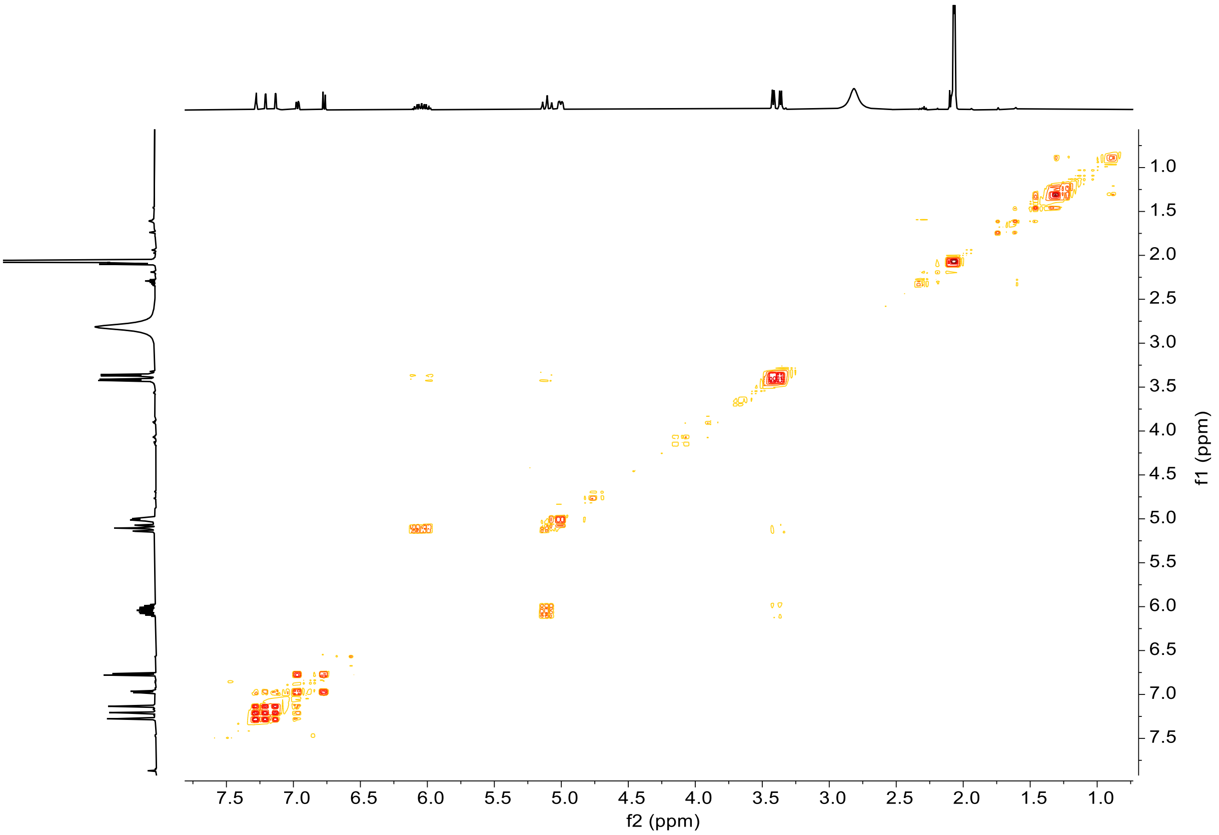

Figure S22. gCOSY of **5**.

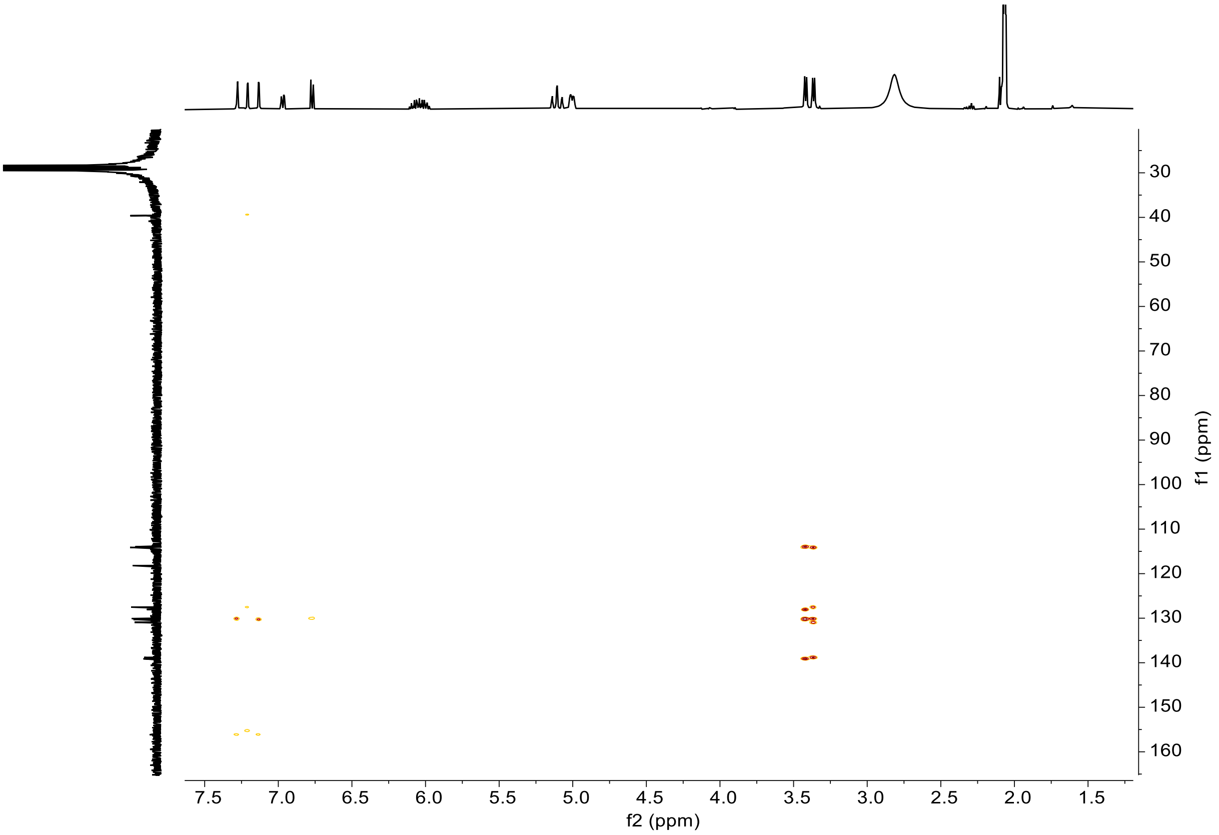

Figure S23. gHMBC of **5**.

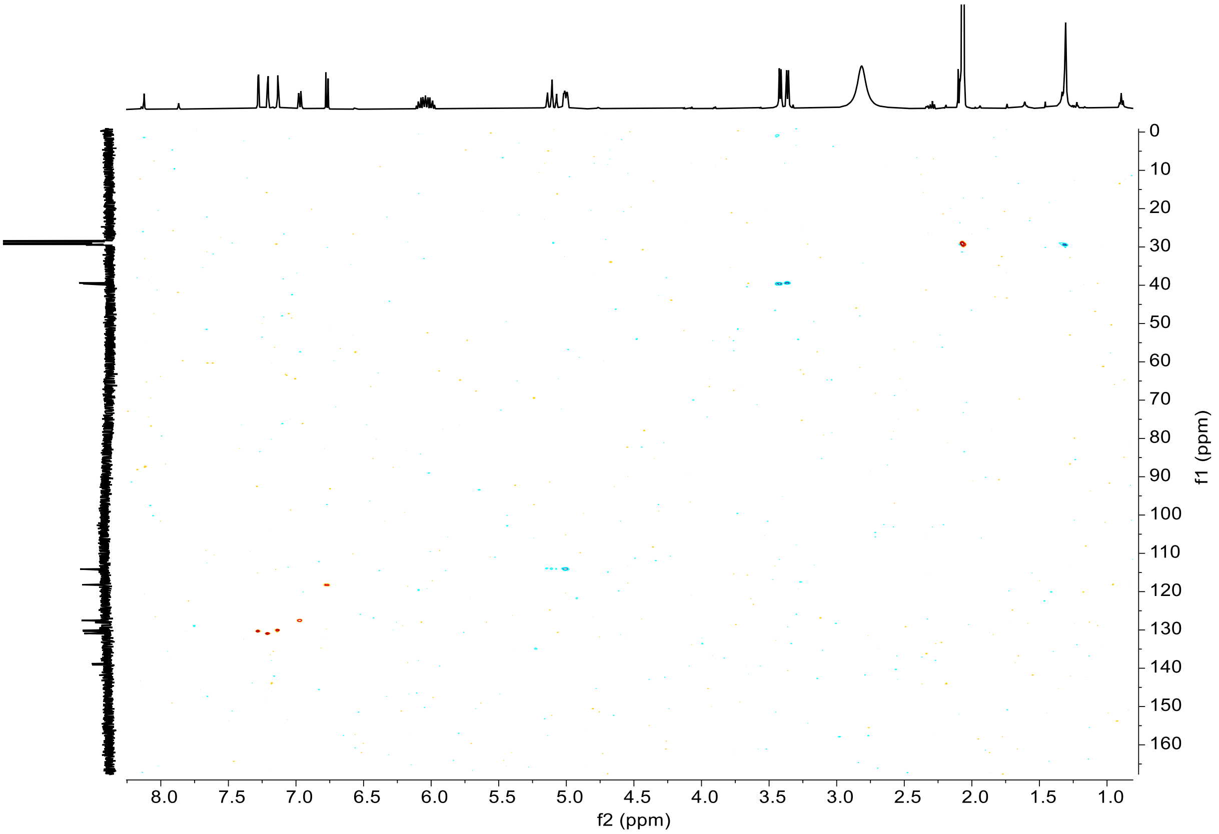

Figure S24. gHSQC spectrum of **5**.

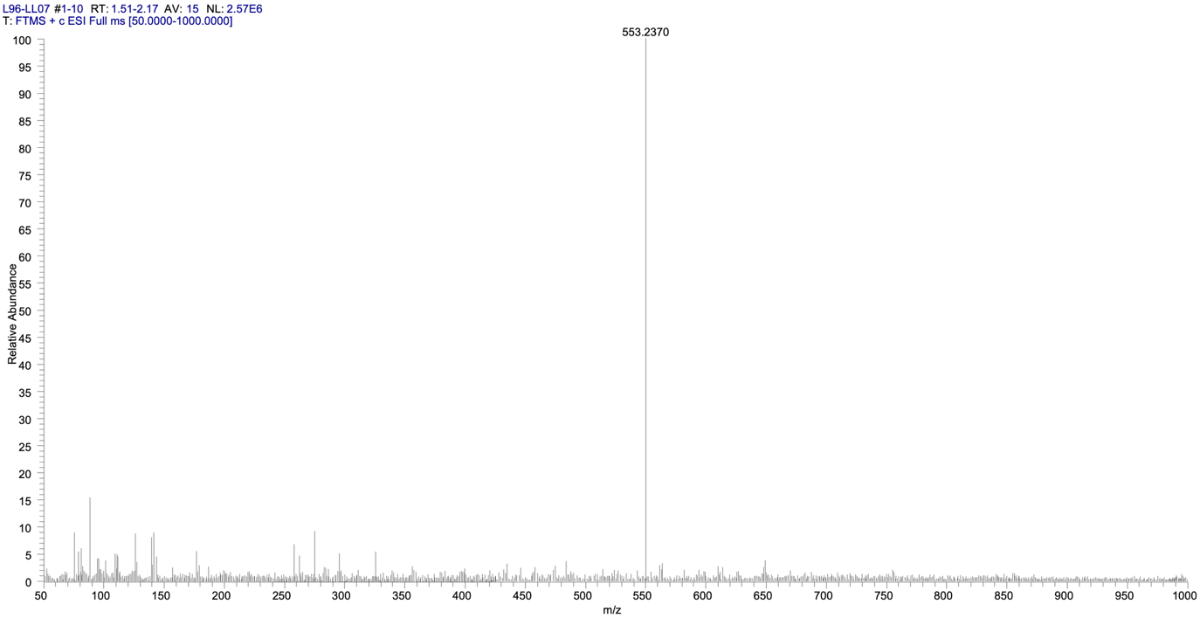

Figure S25. HRMS $[M+Na]^+$ spectrum of **6**.

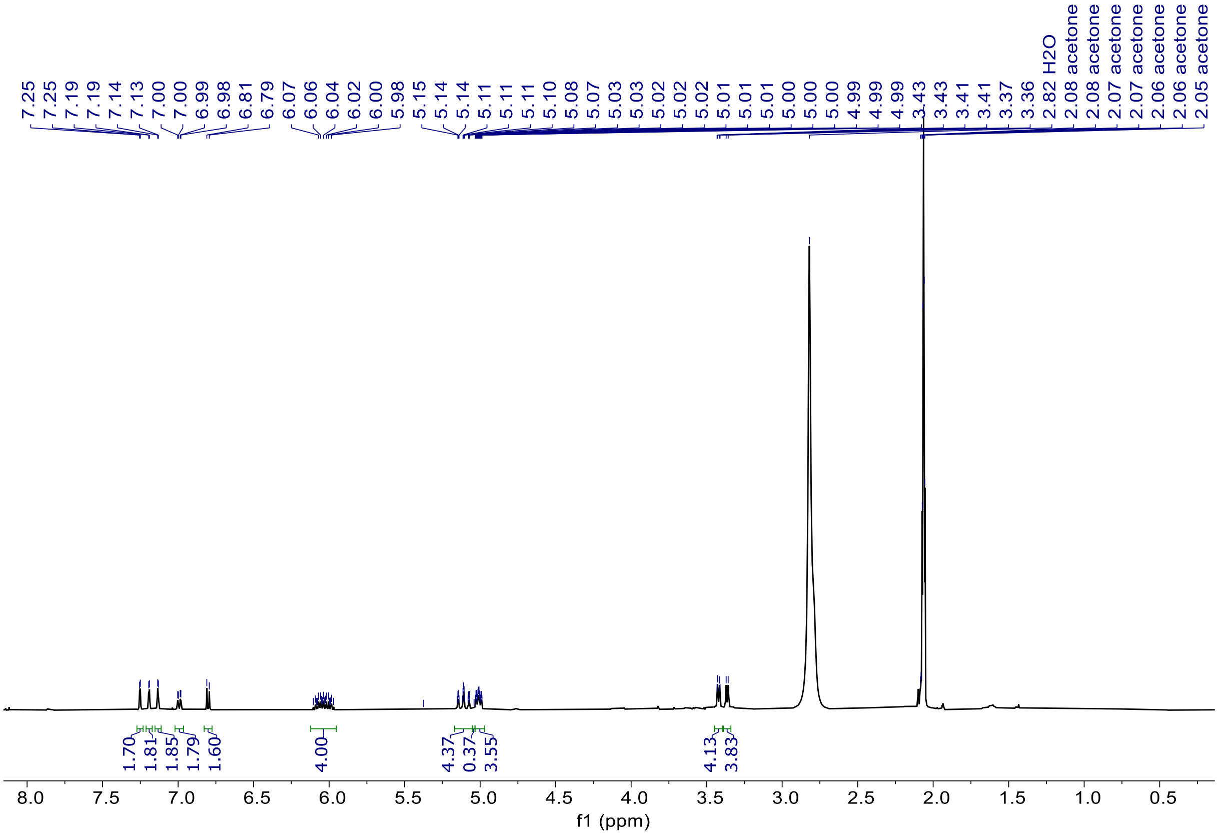

Figure S26. ^{1}HNMR spectrum (500 MHz, $(CD_3)_2CO$) of **6**.

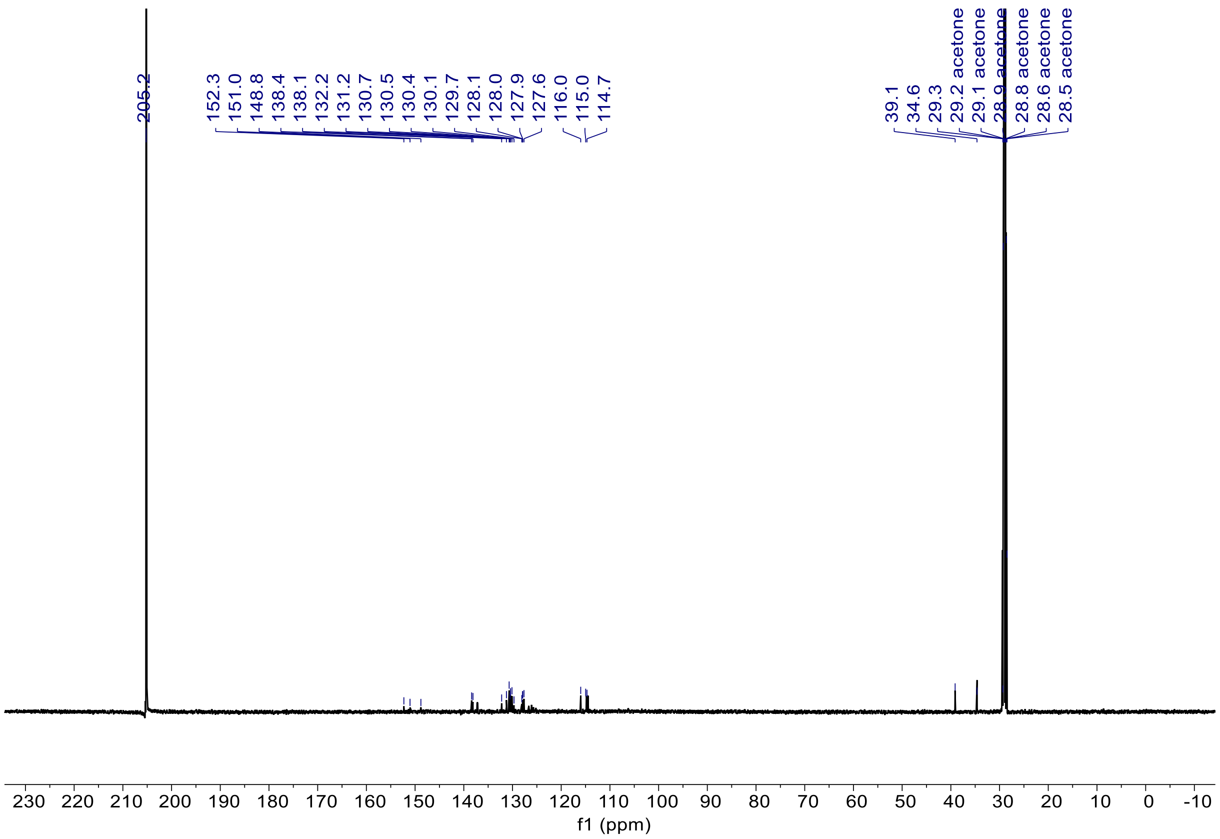

Figure S27. ^{13}CNMR spectrum (125 MHz, $(CD_3)_2CO$) of **6**.

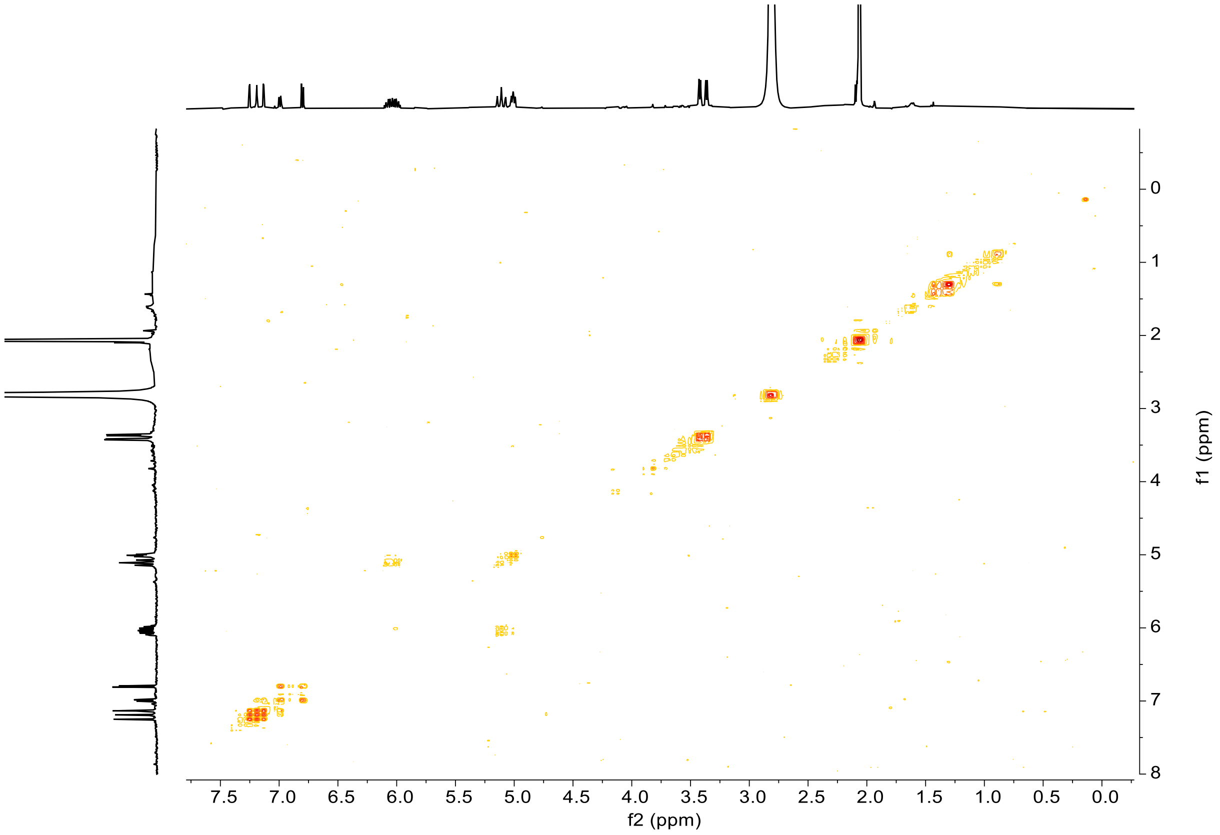

Figure S28. gCOSY of **6**.

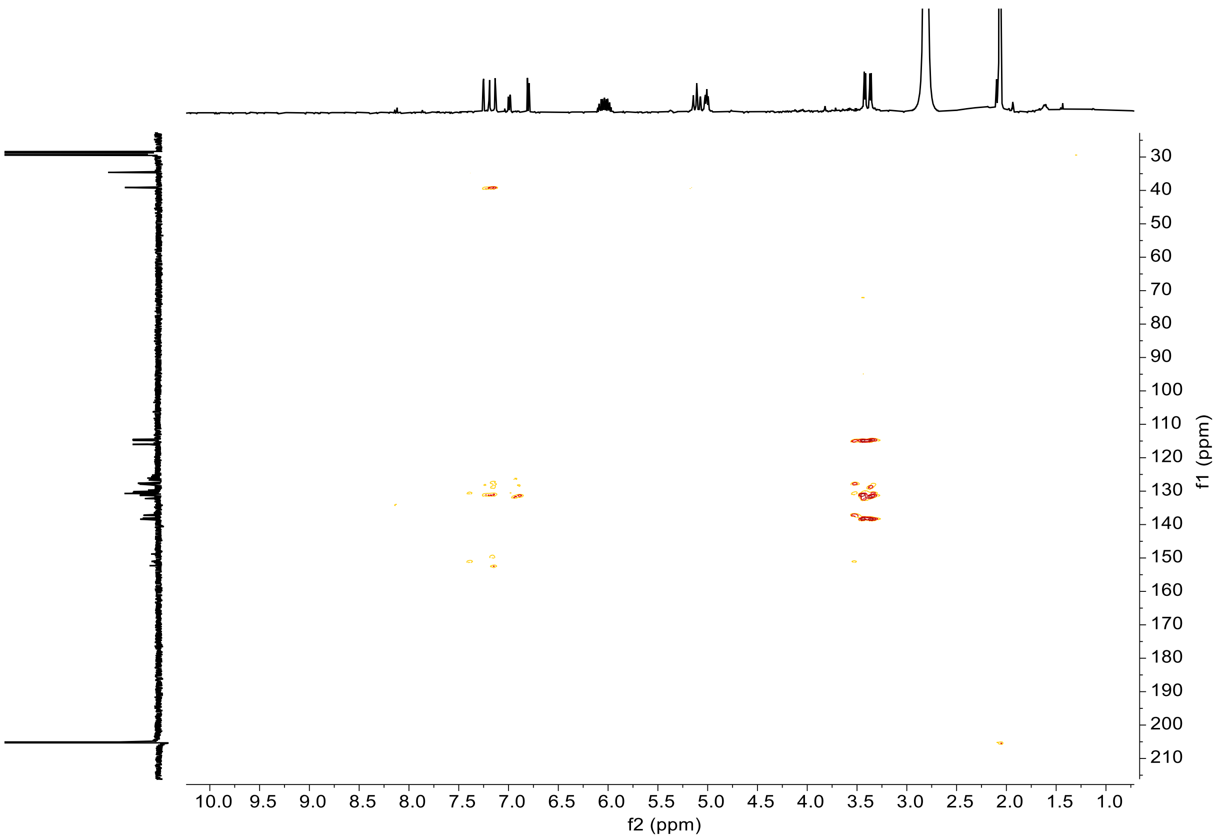

Figure S29. gHMBC of **6**.

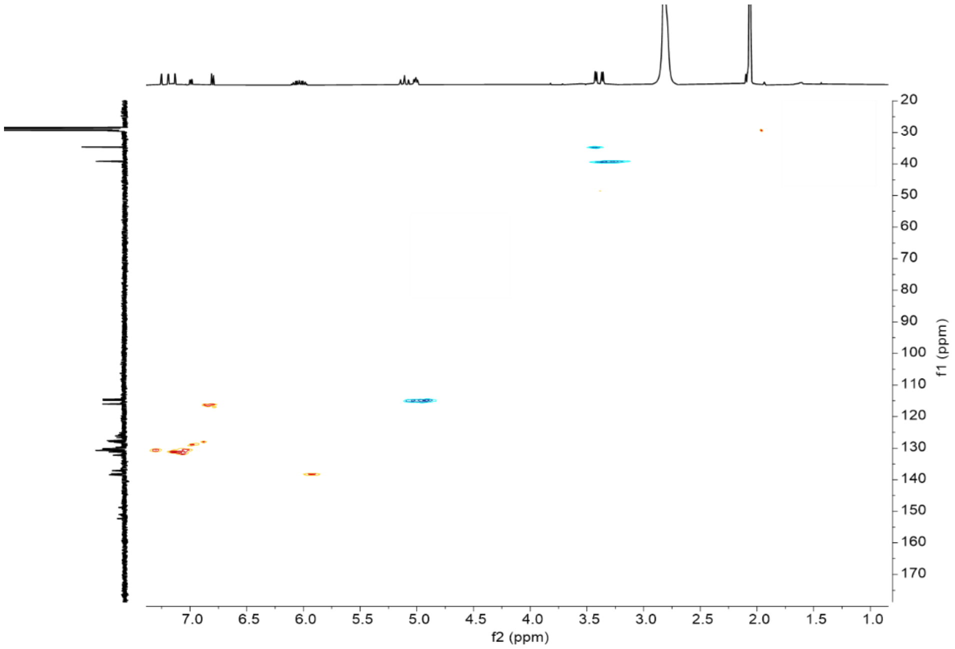

Figure S30. gHSQC of **6**.

.

Assay and kinetic of metabolic enzyme inhibition

Pancreatic lipase (PL) inhibition

The inhibition of porcine PL was performed employing 4-nitrophenyl butyrate as a substrate [17]. Phosphate buffer (50 mM, pH = 7.2; 150 μL), the PL solution (5 mg/mL in phosphate buffer; 15 μL), and different aliquots (4, 6, 8, 10, and 15 μL) of tested compounds (MeOH solutions ranging from 1.88 mM to 0.94 mM) or of orlistat (6.7 μM in buffer) were mixed. The reactions were incubated at 37 °C for 10 min. Then, the substrate (3.2 mM in H_2O:DMF 70:30, 10 μL) was added, and the microplate was incubated at 37 °C for 30 min. Optical density (OD) was acquired at 405 nm using the Synergy H1 microplate reader (BioTek, Bad Friedrichshall, Germany). The experiments were performed in triplicate with different concentration for each compound and the %inhibition was determined by the following equation:

$$\%inhibition = \frac{OD_{control} - OD_{sample}}{OD_{control}} * 100\% \quad (1)$$

The mode of inhibition and the inhibitory constants for the most promising analogues were determined similarly. The experiments were performed in 96-well plates employing the enzyme (120 U/mL in phosphate buffer; 10 μL), the selected molecules, and the increasing concentrations of *p*-nitrophenyl butyrate (from 0.3 to 1.9 mM) in a final volume of 200 μL. The OD was read at 405 nm every 1 min for 45 min at 37 °C. The initial velocity was determined as the slope of the OD changes at 405 nm during the linear phase of the reaction and the inhibitory constants were calculated using the corresponding equations:

$$v_0 = \frac{v_{max} S}{k_m\left(1+\frac{1}{k_i}\right)+S} \quad (2)$$

$$v_0 = \frac{v_{max} S}{k_m\left(1+\frac{1}{k_i}\right)+S(1+\frac{1}{k_{i'}})} \quad (3)$$

α-Glucosidase inhibition (α-Glu)

Briefly, in a 96-well microplate, the α-Glu solution (0.25 U/ml in 50 mM phosphate buffer, pH 6.8; 100 μL) was mixed with tested compounds (2, 4, 6, 8 μL of MeOH solutions ranging from 3.75 mM to 0.65 mM). Then, the substrate *p*-nitrophenyl-α-glucoside (NPG, 78 μM, 100 μL) was added and the microplate was incubated at 37 °C for 30 min under shaking [41]. The reaction was stopped by adding 1 M aqueous Na_2CO_3 (10 μL) and OD was recorded at 405 nm. Acarbose was used as a reference standard. The %inhibition was determined by the following **equation 1**. The mode of inhibition and the inhibitory constants for the most promising analogues were determined similarly. Precisely, mixtures containing α-Glu (5 μL of a 31 μM solution), the inhibitors (**4**: 0, 11.5, 22.1, 33.0, 42.9 μM; **5**: 0, 10.0, 20.0, 30.0 μM; **6**: 0, 8.0, 16.0, 25.0 μM), and NPG (2.00, 1.50, 1.25, 0.83, 0.50, 0.33 and 0.15 mM) were incubated at 37 °C and the optical density was read at 405 nm every 1 min for 30 min with the Synergy H1. The inhibitory constants were calculated using the corresponding **equations 2** and **3**.

α-Amylase inhibition (α-Amy)

The inhibition of the porcine pancreatic α-Amy was performed as previously reported [41]. The reactions were carried out in test tubes by mixing 50 μL of the enzyme solution (6 U/ml in 20 mM phosphate buffer containing 6.7 mM NaCl) with tested compounds (2, 4, 6, 8 of 3.75–0.94 mM solutions). The reactions were incubated at 37 °C for 10 min, then, a starch solution (0.5 % in phosphate buffer; 50 μL), previously stirred at 90 °C for 20 min, was added in the test tubes, and the mixtures were incubated again at 37 °C for 15 min. Lastly, 100 μL of a 96 mM 3,5-dinitrosalicylic acid solution (containing 30 % sodium potassium tartrate in 2 N NaOH) were added, and the test tubes were heated at 80 °C for 10 min. Each mixture was diluted with water (final volume 540 μL) and the solutions were moved into a 96-well microplate, and the OD at 540 nm was acquired. Acarbose was used as a positive reference. The %inhibition was determined by the following **equation 1**. The mode of inhibition and the kinetic parameters for the most promising compounds were determined according to a procedure previously described with some modifications. The assay was performed in 96-well microplates (final volume of 200 μL). In a typical set of experiments were added 10 μL of α-Amy solution (4.0 U/mL in 0.1 mM phosphate buffer containing 0.02 % NaN_3; pH 6.8) and the inhibitor at different concentrations (**4**: 0, 8.5, 17.0, 22.5, 34.0 μM; **5**: 0, 3.0, 8.5, 12.0 μM; **6**: 0, 6.25, 12.5, 25.0 μM). The plate was incubated at 37 °C for 10 min, and the reaction was started by addition of different aliquots of 10 mM 2-chloro-4-nitrophenyl-α-maltotrioside (CNPG3; 1.25, 1.00, 0.75, 0.50, 0.25 mM), the OD was measured at 405 nm every minute for 30 min, maintaining the plate at 37 °C. The inhibitory constants were calculated using the corresponding **equations 2** and **3**.

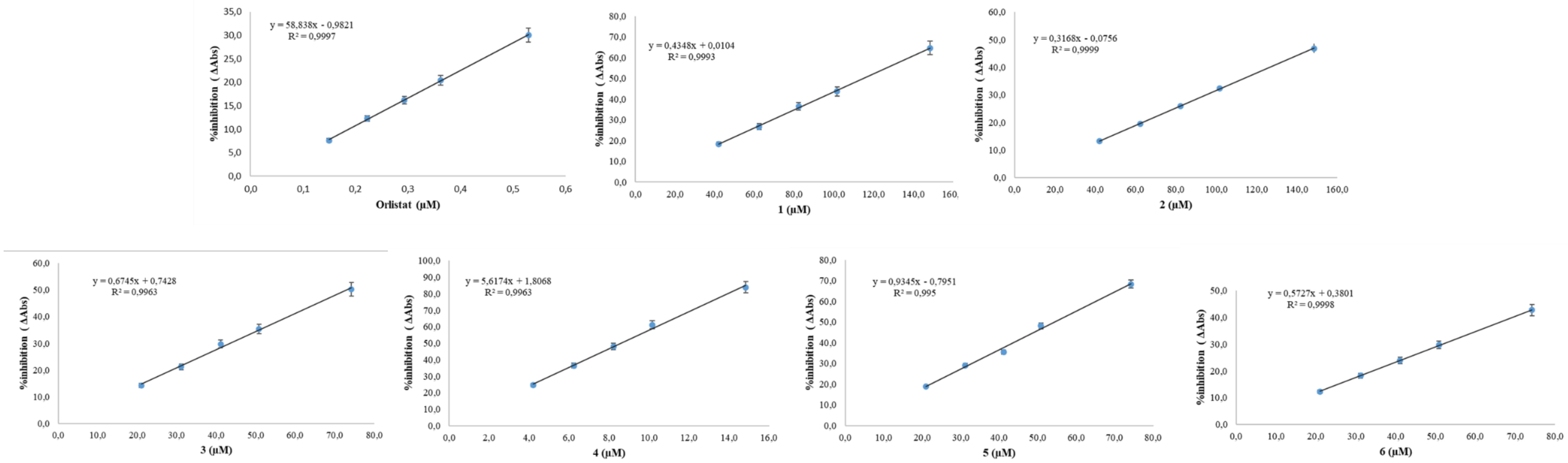

Figure S31. PL inhibitory activity of orlistat, compounds **1**, **2** and synthesized dimers **3** - **6**. Data are presented as means ± standard deviation (SD) of n = 3 independent experiments.

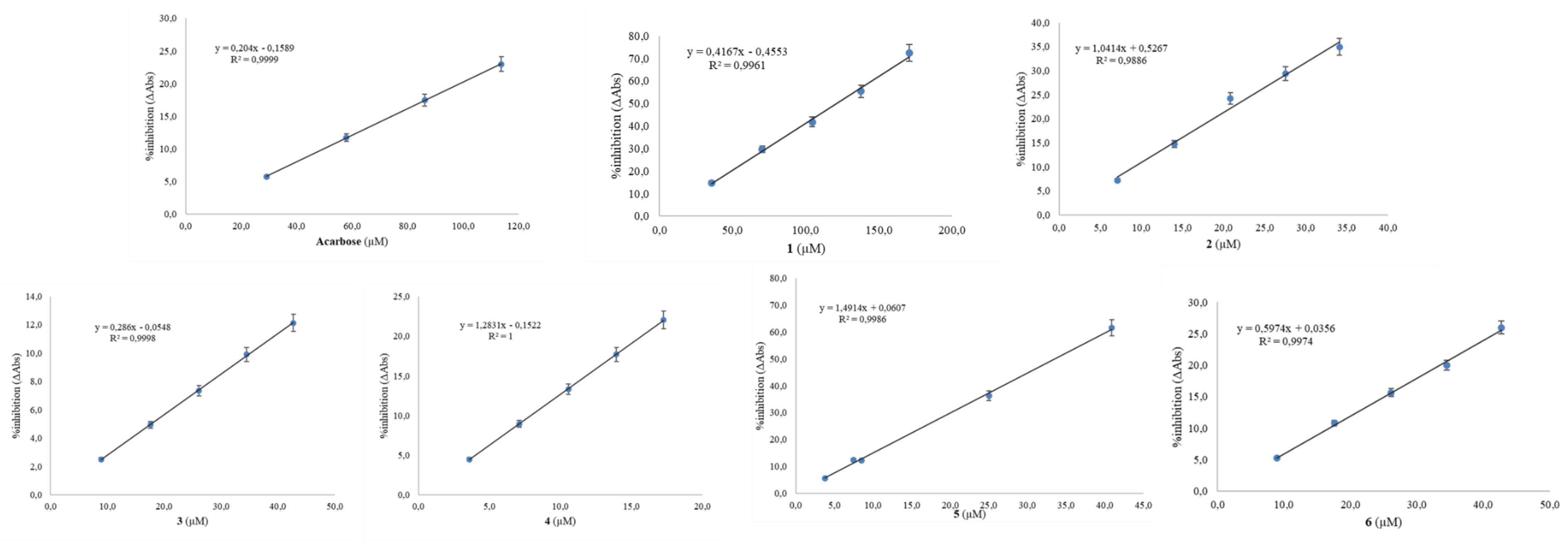

Figure S32. α-Glu inhibitory activity of acarbose, compounds **1**, **2** and synthesized dimers **3** - **6**. Data are presented as means ± SD of n = 3 independent experiments.

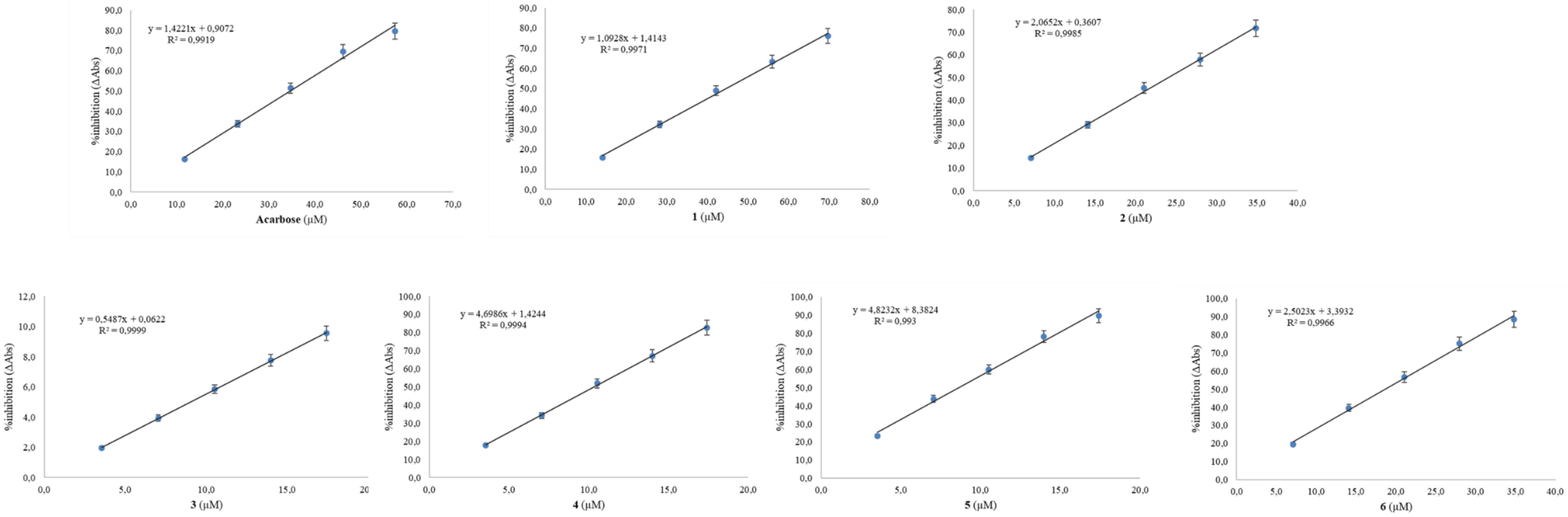

Figure S33. α-Amy inhibitory activity of acarbose, compounds **1**, **2** and synthesized dimers **3** - **6**. Data are presented as means ± SD of n = 3 independent experiments.

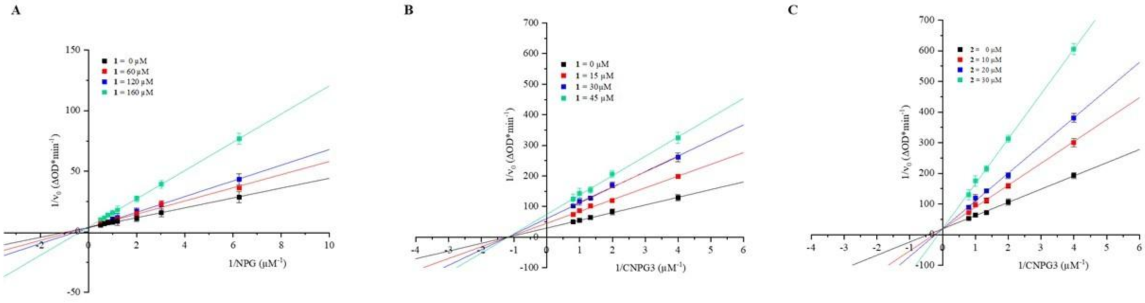

Figure S34. L-B plots showing enzyme inhibition: (A) α-Glu inhibition in presence of **1**, (B) α-Amy inhibition in presence of **1** and (C) α-Amy inhibition in presence of **2.** Data are presented as means ± SD of n = 3 independent experiments.

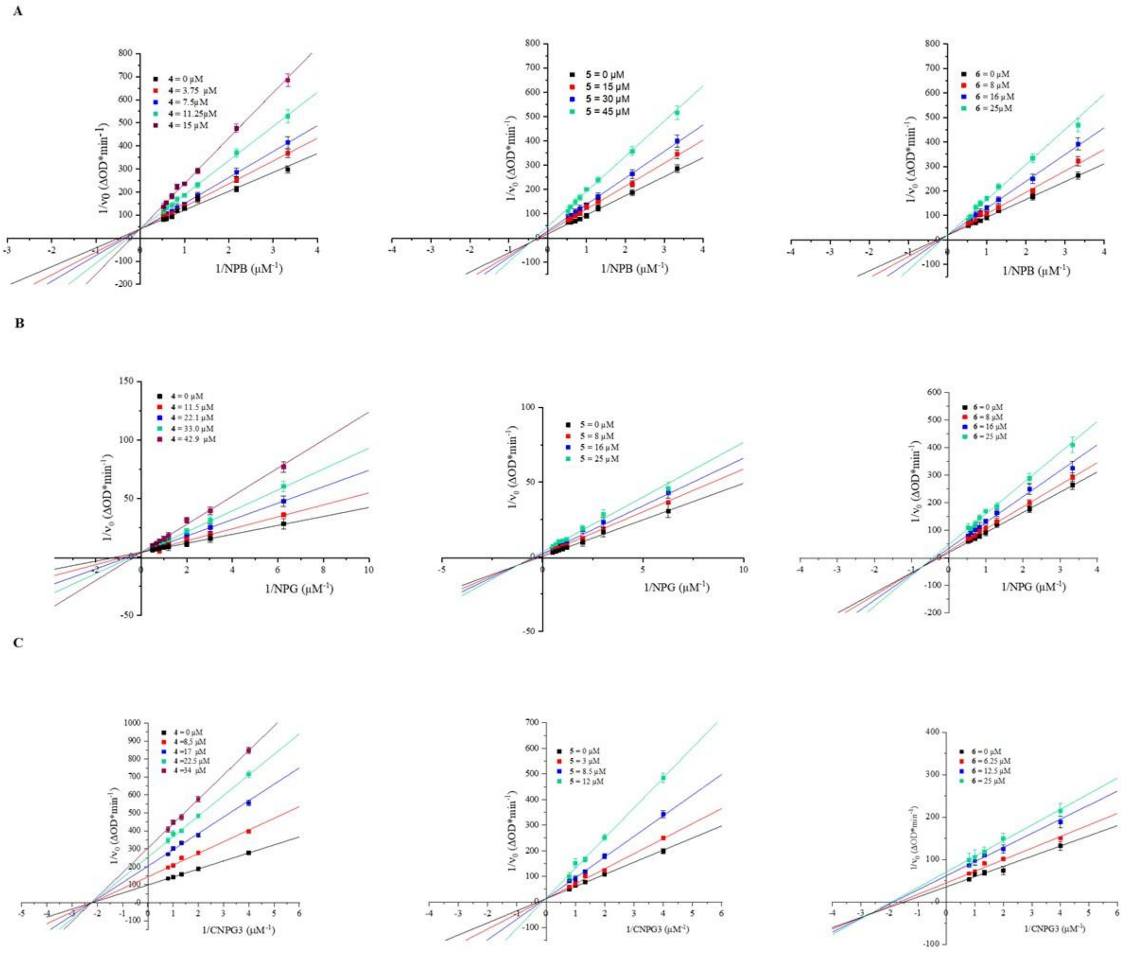

Figure S35. L-B plots showing enzyme inhibition: (A) PL inhibition in presence of **4, 5** and **6**, (B) α-Glu inhibition in presence of **4, 5** and **6** and (C) α-Amy inhibition in presence of **2.** Data are presented as means ± SD of n = 3 independent experiments.

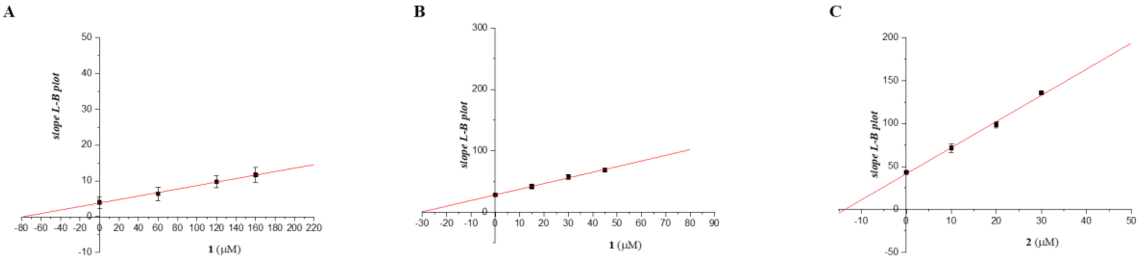

Figure S36. The secondary plots obtained from slope (for K_i determination) of Lineweaver-Burk plots vs inhibitors concentration: **1** and **2**. Data obtained from (A) α-Glu and (B) α-Amy inhibition of HN and (B) α-Amy inhibition of MG. Data are presented as means ± SD of *n* = 3 independent experiments

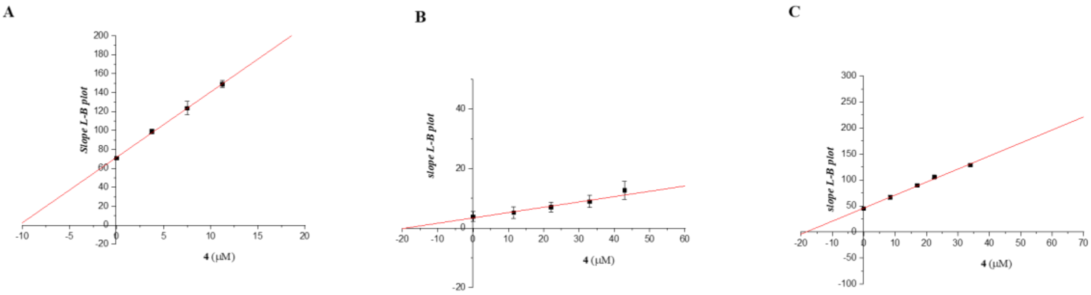

Figure S37.The secondary plots obtained from slope (for Ki determination) of L-B plots vs inhibitor concentration: **4**. Data obtained from (A) PL, (B) α-Glu, (C) α-Amy inhibition. Data are presented as means ± SD of *n* = 3 independent experiments

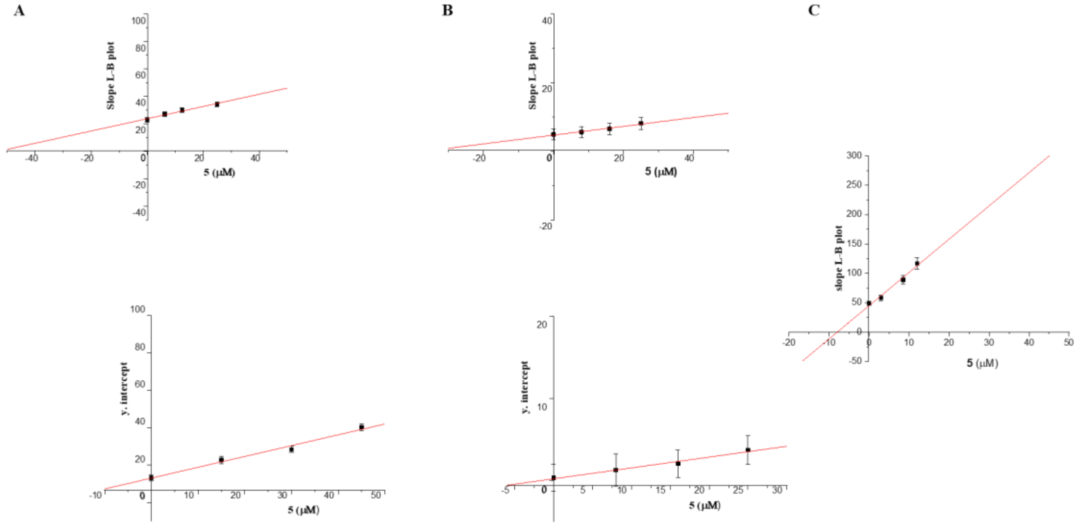

Figure S38. The secondary plots obtained from slope (for Ki determination) of Lineweaver-Burk plots vs inhibitor concentration: **5**. (A) PL, (B) α-Glu, (C) α-Amy inhibition. Data are presented as means ± SD of *n* = 3 independent experiments.

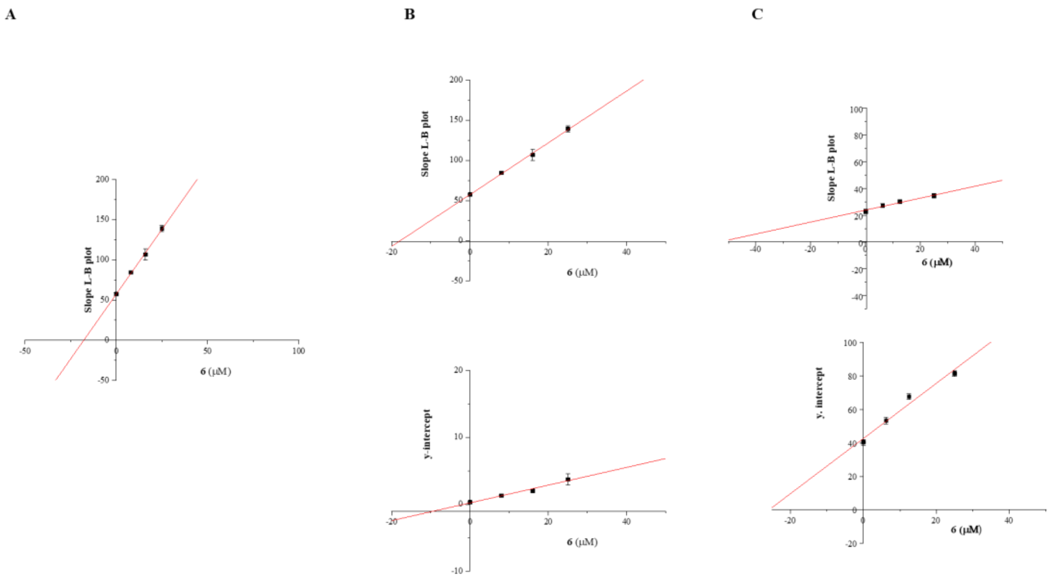

Figure S39. The secondary plots obtained from slope (for Ki determination) of Lineweaver-Burk plots vs inhibitor concentration: **6**. Data obtained from (A) PL, (B) α-Glu, (C) α-Amy inhibition. Data are presented as means ± SD of n = 3 independent experiments

Molecular docking analysis

Molecular docking analysis of the selected compounds on PL, α-Glu and α-Amy was evaluated *in silico*, following the methodology outlined in our previous studies [17, 41]. Docking studied were performed using the Glide Ligand Docking interfaced with the Maestro (Version 13.7). The .sdf files of orlistat, acarbose, MG and HN were downloaded from Pubchem (https://pubchem.ncbi.nlm.nih.gov/, ID cod respectively: 3034010, 41774, 73300 and 72303). The ligands **3** – **6** were drawn by chemdraw and saved in .sdf files. Optimized potential liquid simulation (OPLS3) [45] were employed to geometrically minimized the 3D models. The LigPrep tab was set up considering the protonation state at pH of 7.0 ± 1 and processed by the interfaced with Maestro (Version 13.7) of Schröedinger suite. The 3D structure of PL and porcine pancreatic α-Amy (PDB ID: 1LPB and 1OSE) were downloaded from Protein Data Bank (https://www.rcsb.org/). Whereas the 3D protein model of α-Glu from *Saccharomyces cerevisiae* was downloaded from AlphaFold Protein structure Database (https://alphafold.ebi.ac.uk/, PDB code: AF-P53341-F1-model_v4) [46]. Each of these 3D protein models was first prepared and minimized by Protein preparation wizard. The putative binding site of α-Glu was identified through SiteMap software, that allowed to generate a ranking of five possible druggable binding pockets based on the site score output and the best-one was then used for the molecular docking calculation. By the site 1, that one with the best site score, the following coordinates were obtained by Grid generation experiment: 14.61 (x), 2.13 (y), 1.91 (z), 10 x 10 x 10 as innerbox, and 36.76 x 36.76 x 36.76 as outerbox. The grid box α-amylase and lipase were constructed by selecting in the Receptor Grid Generation tab, the co-crystallized inhibitor (acarbose for α-Amy and orlistat for PL respectively) to specify the protein region suitable as binding site. In this way was calculated the grid box of proteins, with grid centre set to 35.61 (x), 37.36 (y) and -1.35 (z), 20 x 20 x 20 as innerbox, and 40.80 x 40.80 x 40.80 as outerbox for α-amylase; whereas for lipase the grid centre was set to 6.52, 22.27 and 43.85 10 x 10 x 10 as innerbox, and 30 x 30 x 30 as outerbox. The molecular docking studies were performed using Glide Ligand Docking interfaced with Maestro. In Docking calculation, ligands were treated as flexible, and protein were treated as rigid. Glide were compiled and run in OSX Ventura (13.2) environment. The analysis of docking outcomes was carried out by Maestro (Version 13.7). The images were generated using Maestro visualizer, with rendering quality set to custom at 600 DPI and an output of 5976 x 3119 pixels. All images were exported in PNG format, and the relevant visual details including interacting residues, and interaction types are described in the corresponding figure captions.

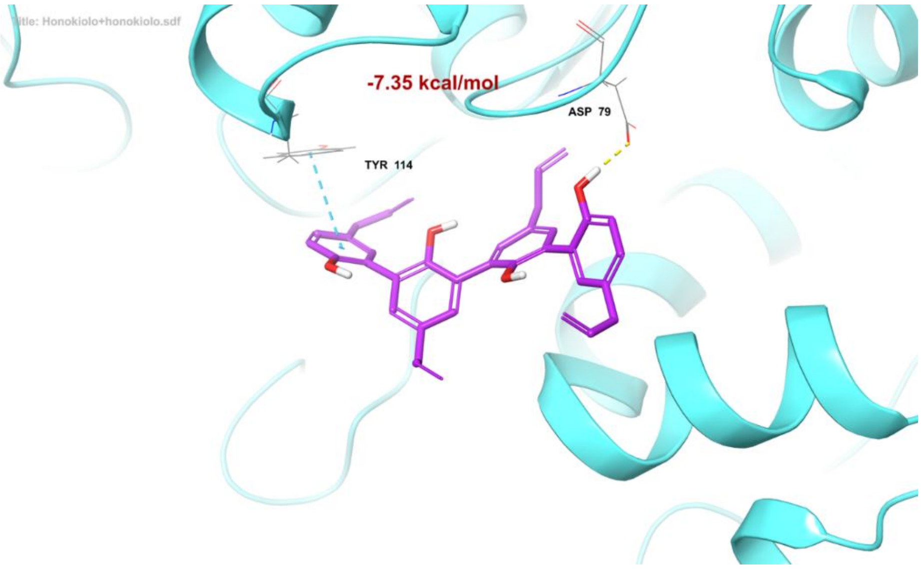

Figure S40. Compound **5** (plum) into PL (cyan binding site); the interactions are depicted in yellow (hydrogen bonds), cyan (aromatic H-bond) faded azure (pi-pi stacking) and green (pi-cation); only interactive residues are labelled. The color coding used for atoms is red (O), blue (N) and grey (C).

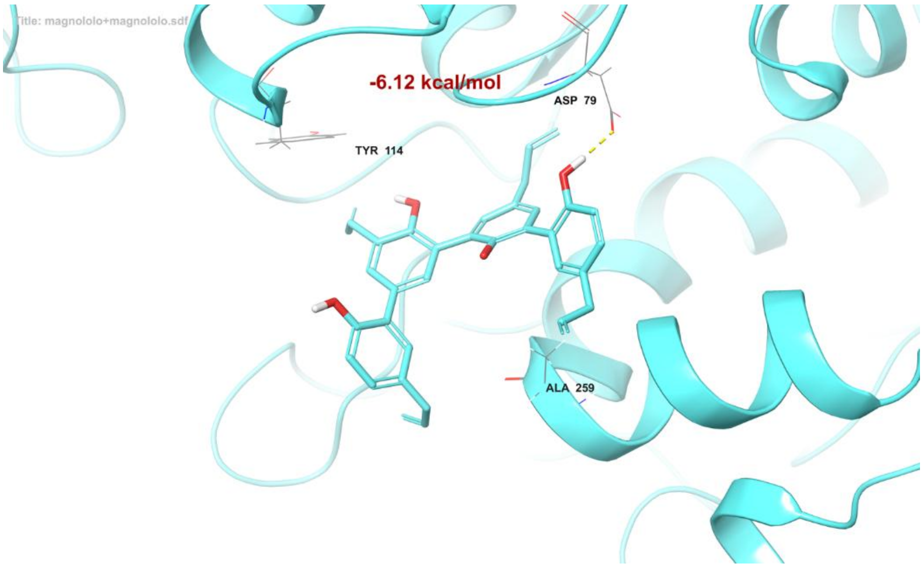

Figure S41. Compound **6** (cyan) into PL (cyan binding site); the interactions are depicted in yellow (hydrogen bonds), cyan (aromatic H-bond) faded azure (pi-pi stacking) and green (pi-cation); only interactive residues are labelled. The color coding used for atoms is red (O), blue (N) and grey (C).

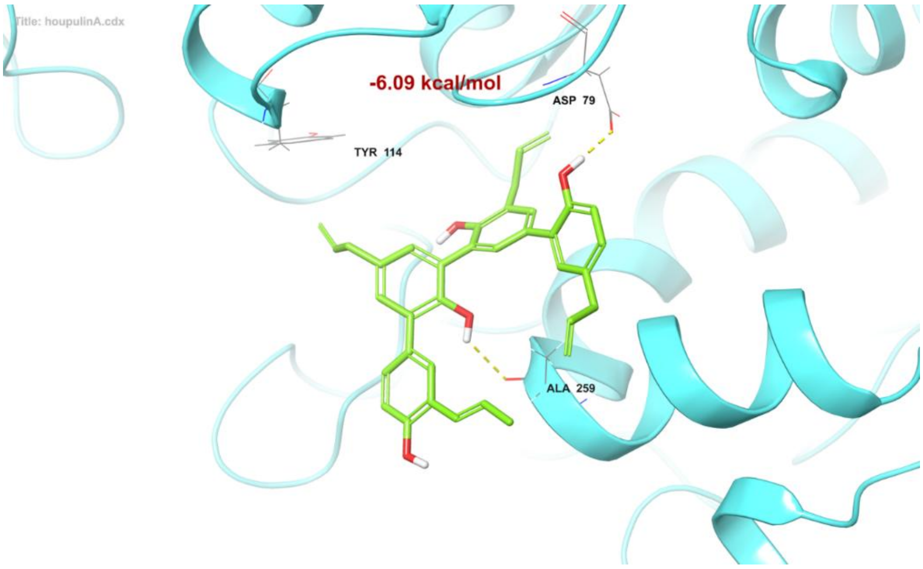

Figure S42. Compound **3** (yellow-green) into PL (cyan binding site); the interactions are depicted in yellow (hydrogen bonds), cyan (aromatic H-bond) faded azure (pi-pi stacking) and green (pi-cation); only interactive residues are labelled. The color coding used for atoms is red (O), blue (N) and grey (C).

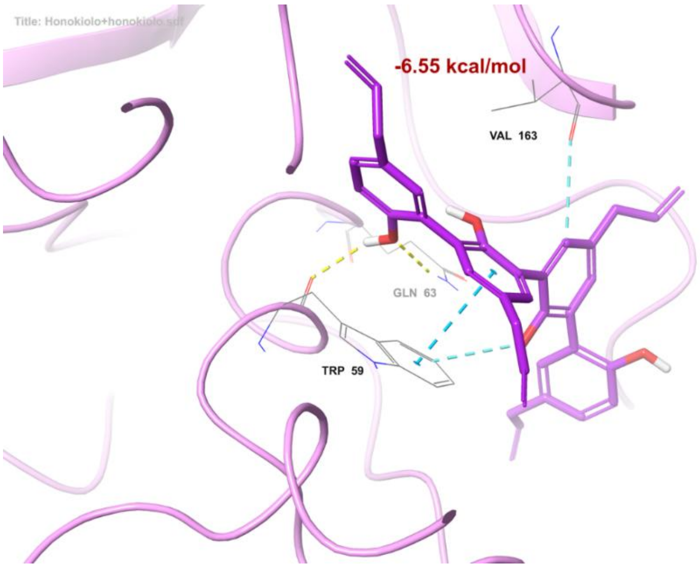

Figure S43. Compound **5** (plum) into α-Amy (magenta binding site); the interactions are depicted in yellow (hydrogen bonds), cyan (aromatic H-bond) faded azure (pi-pi stacking) and green (pi-cation); only interactive residues are labelled. The color coding used for atoms is red (O), blue (N) and grey (C).

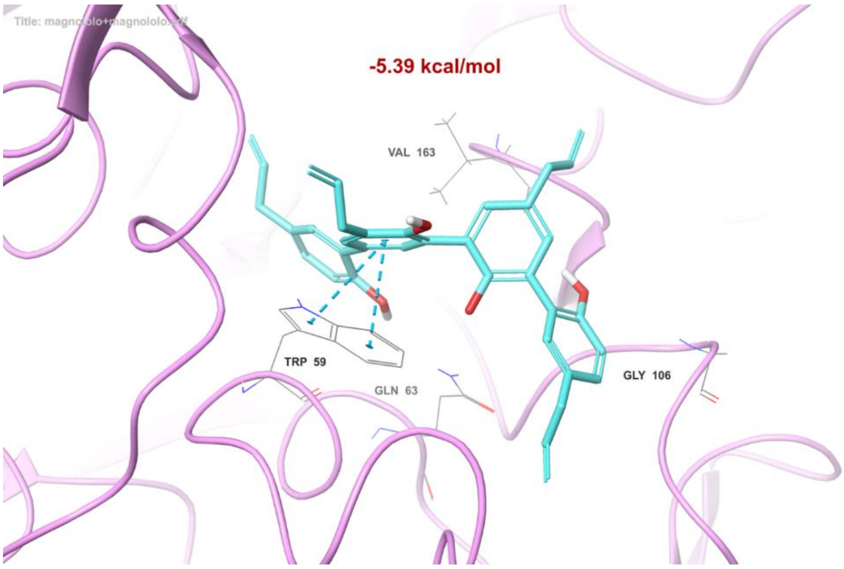

Figure S44. Compound **6** (cyan) into α-Amy (magenta binding site); the interactions are depicted in yellow (hydrogen bonds), cyan (aromatic H-bond) faded azure (pi-pi stacking) and green (pi-cation); only interactive residues are labelled. The color coding used for atoms is red (O), blue (N) and grey (C).

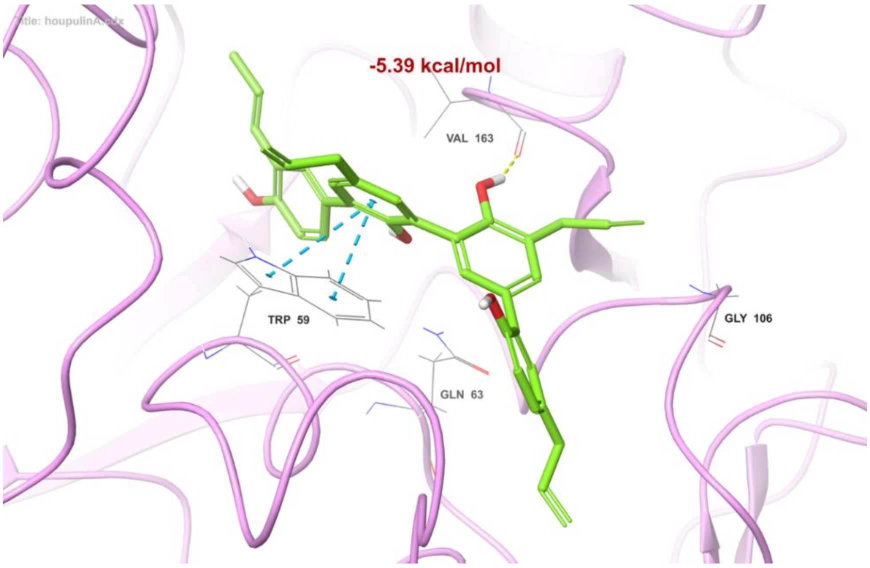

Figure S45. Compound **3** (yellow-green) into α-Amy (magenta binding site); the interactions are depicted in yellow (hydrogen bonds), cyan (aromatic H-bond) faded azure (pi-pi stacking) and green (pi-cation); only interactive residues are labelled. The color coding used for atoms is red (O), blue (N) and grey (C).

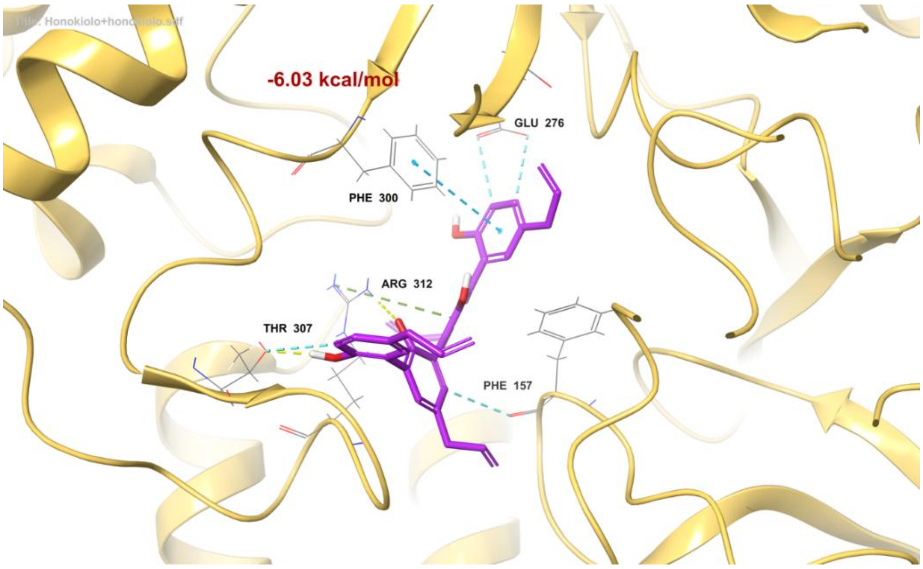

Figure S46. Compound **5** (faded blue) into α-Glu (faded-orange binding site); the interactions are depicted in yellow (hydrogen bonds), cyan (aromatic H-bond), faded azure (pi-pi stacking) and green (pi-cation); only interactive residues are labelled. The color coding used for atoms is red (O), blue (N) and grey (C).

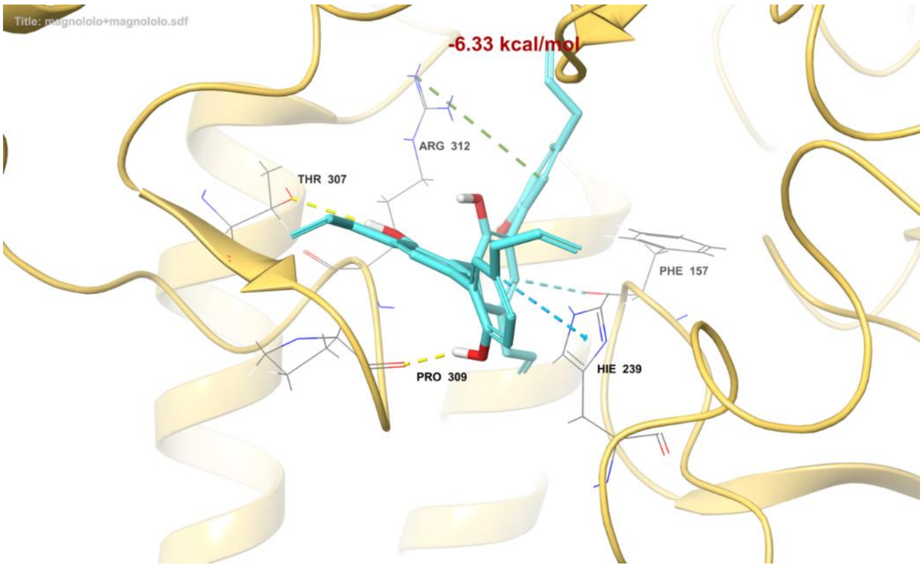

Figure S47. Compound **6** (cyan) into α-Glu (faded-orange binding site); the interactions are depicted in yellow (hydrogen bonds), cyan (aromatic H-bond) faded azure (pi-pi stacking) and green (pi-cation); only interactive residues are labelled. The color coding used for atoms is red (O), blue (N) and grey (C).

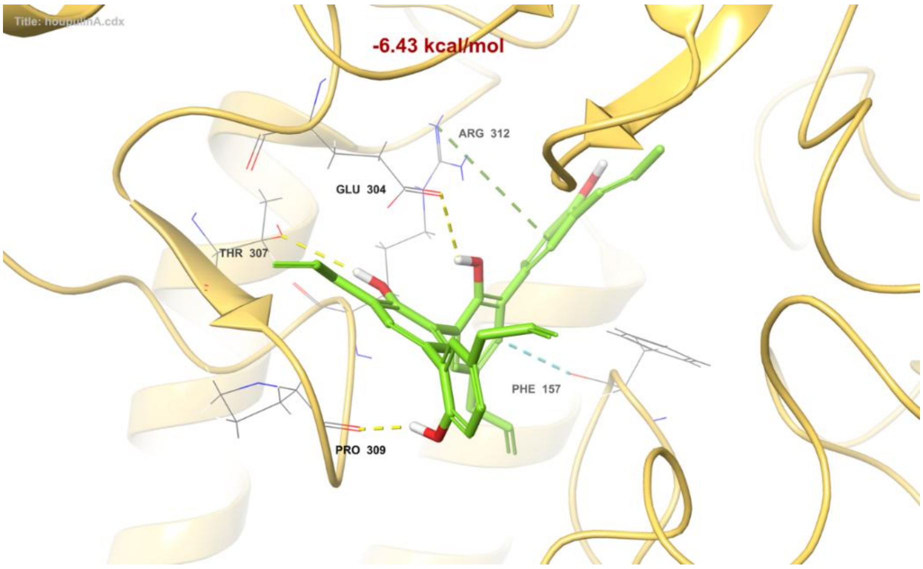

Figure S48. Compound **3** (yellow-green) into α-Glu (faded-orange binding site); the interactions are depicted in yellow (hydrogen bonds), cyan (aromatic H-bond) faded azure (pi-pi stacking) and green (pi-cation); only interactive residues are labelled. The color coding used for atoms is red (O), blue (N) and grey (C).

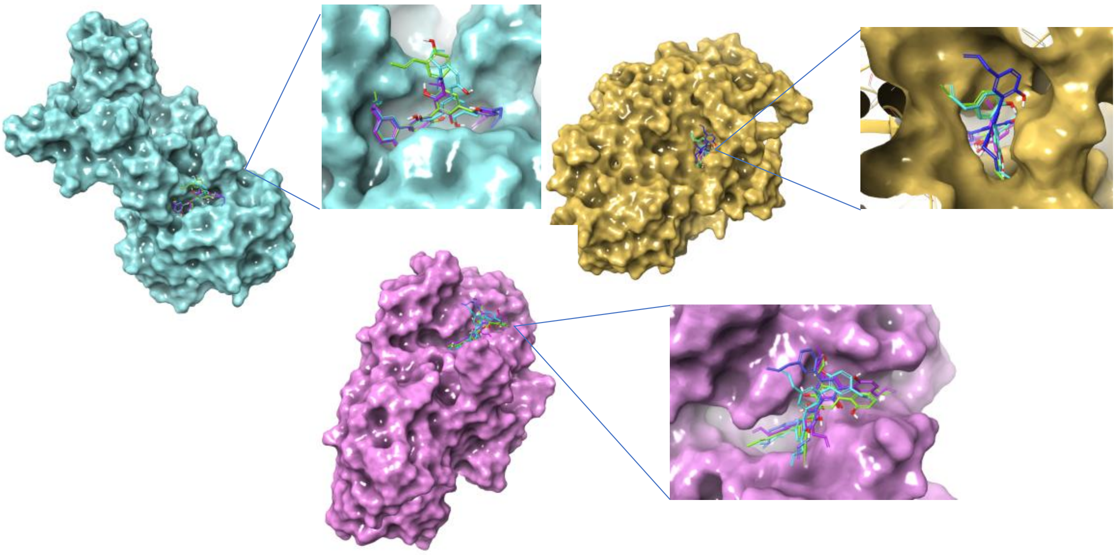

Figure S49. Molecular surface of PL (cyan), α-Amy (faded orange), α-Glu (magenta) with **3** - **6** into the BS.

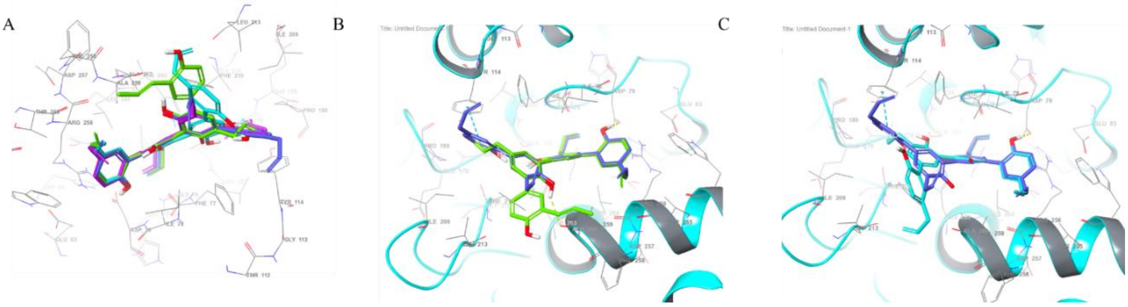

Figure S50. PL binding site and overlap of: (A) **3** – **6**; (B) **3** and **4**; (C) **4** and **6**.

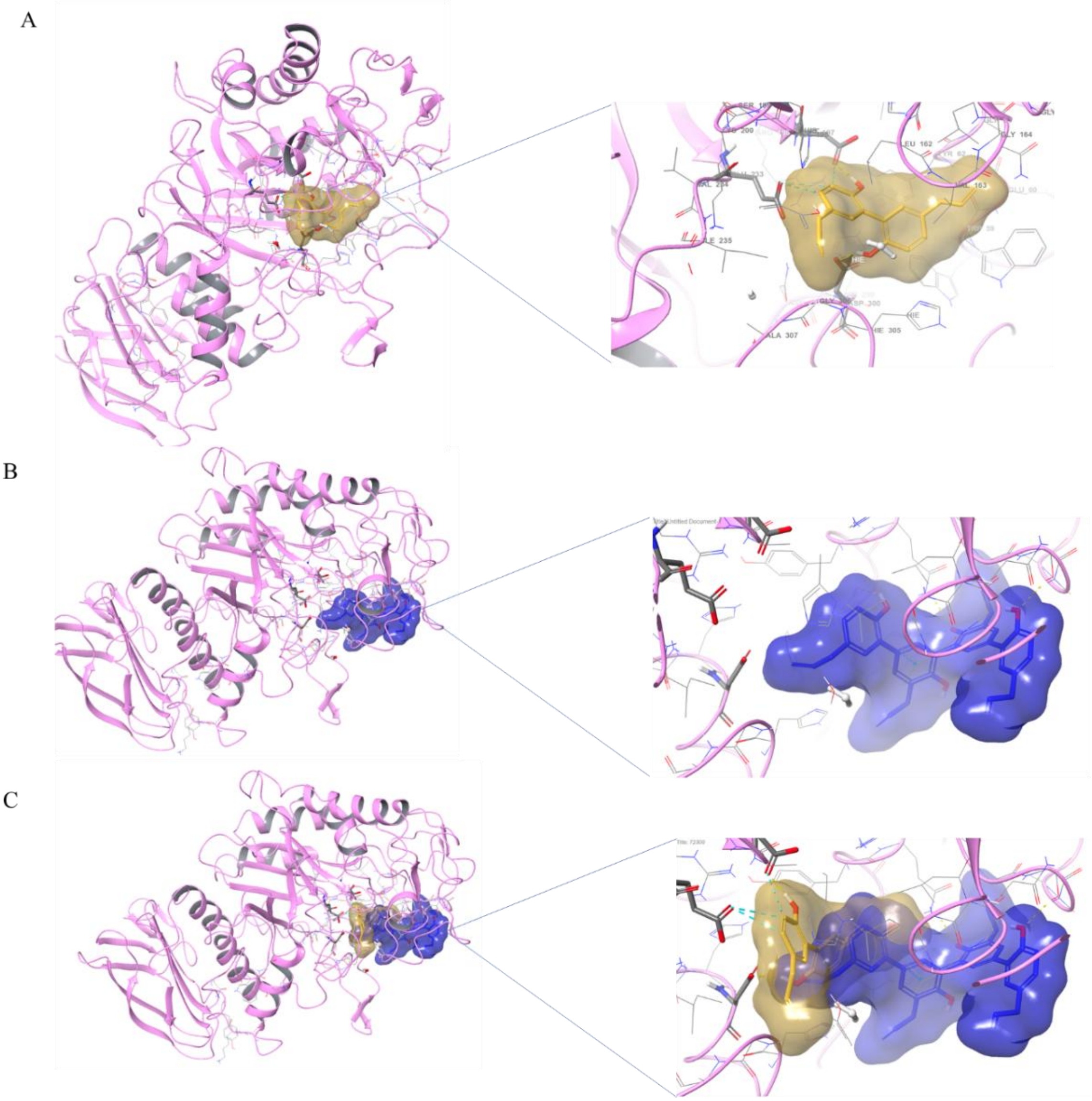

Figure S51. 1OSE detail on the surface of (A) **4**; (B) **1**; (C) **1** and **4**.

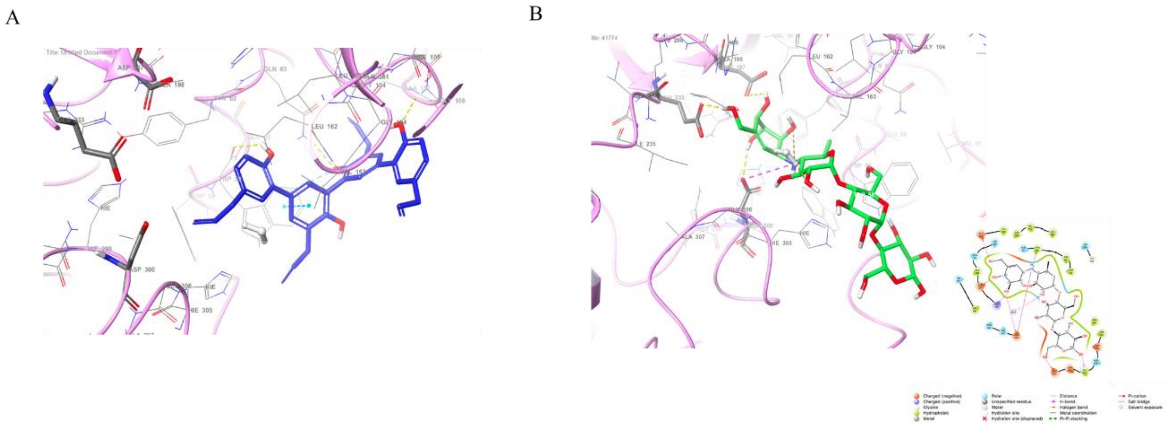

Figure S52. 1OSE detail of α-amylase catalytic triad and (A) **4** and (B) hypoglycaemic drug acarbose.

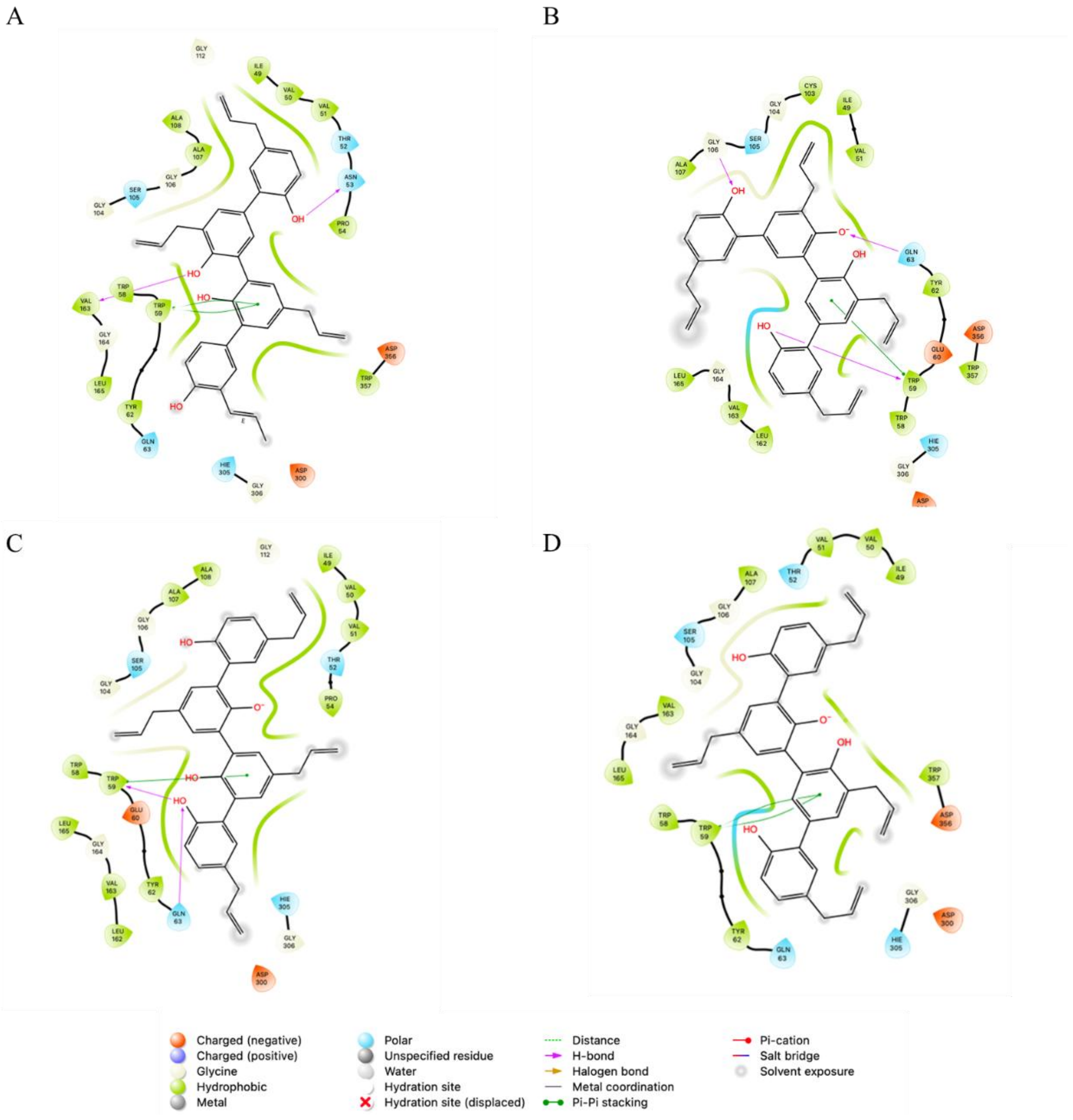

Figure S53. Interaction details of dimer (A) **3**, (B) **4**, (C) **5** and (D) **6** and α-Amy catalytic site.

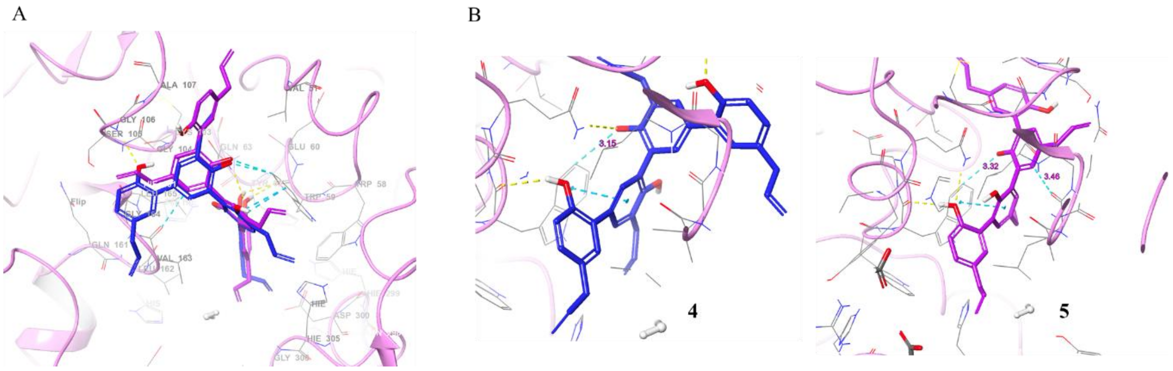

Figure S54. (A) α-Amy - overlap 1OSE dimer **4** and **5**; B) 1OSE detail interaction.

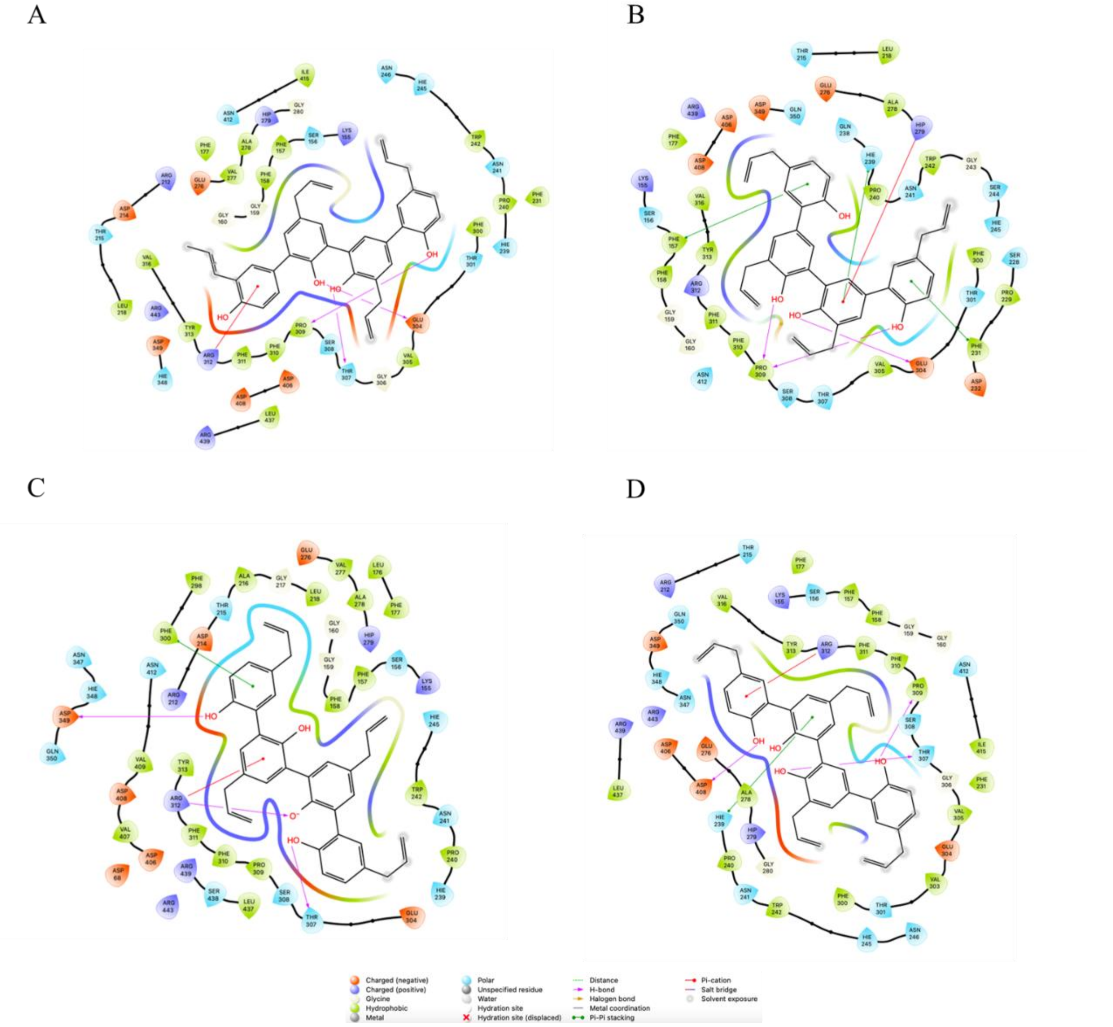

Figure S55. Interaction details of dimer A) **3**, B) **4**, C) **5** and D) **6** and α-Glu catalytic site.

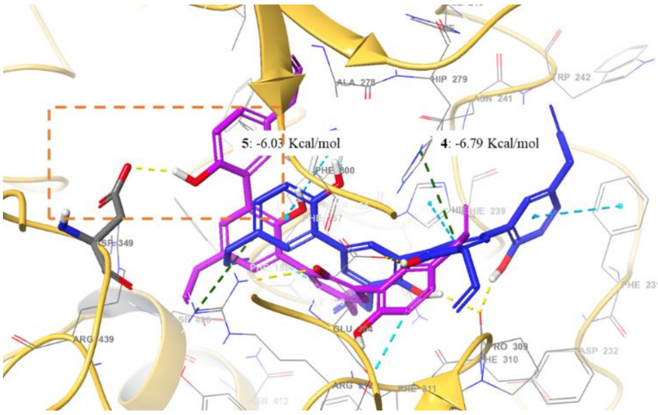

Figure S56. Detail of Asp 349 interaction in the deepest portion of the hydrophobic pocket and comparison with dimer **4**.

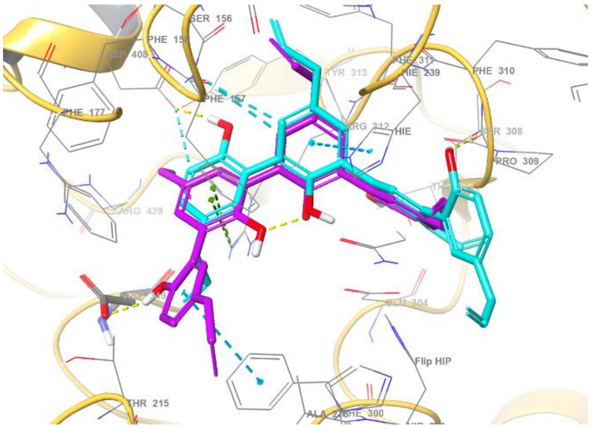

Figure S57. α-Glu -overlap of **5** and **6**.

Table S8. Binding energies (ΔG_{bind}) and interacting residues of ligands (**1 - 6**) with catalytic site.[a]

**Ligands**	**α-Glu calcd ΔG_{bind}**	**α-Amy calcd ΔG_{bind}**	**PL calcd ΔG_{bind}**
**orlistat**	-	-	-3.45[b]
**acarbose**	-7.09[b]	-7.02	-
**1**	-5.48[b]	-5.95	-6.62[b]
**2**	-5.02[b]	-4.81	-5.55[b]
**3**	-5.43	-6.66	-6.09
**4**	-6.79	-6.87	-6.02
**5**	-6.03	-6.55	-7.35

**6**	-6.33	-5.39	-6.12

[a]The ΔG_{bind} values were calculated with Glide and are expressed as Kcal/mol. [b] Data previously reported in the literature by us [17].

Table S9. List of molecular interactions of **1** – **6** with PL Catalytic Site.

**Ligands**	**Interacting residues**	**Interaction**	**Distance** (A°)
**3**			
OH (A-ring)	Asp79	H-bond	1.78
OH (C-ring)	Ala259	H-bond	2.66
**4**			
D ring	Tyr114	Pi-Pi stacking	4.31
OH (A ring)	Asp79	H-bond	1.77
**5**			
D ring	Tyr114	Pi-Pi stacking	4.15
OH (A ring)	Asp79	H-bond	1.78
**6**			
OH (A ring)	Asp79	H-bond	1.79

Table S10. List of molecular interactions of **1** – **6** with α-Amy Catalytic Site.

**Ligands**	**Interacting residues**	**Interaction**	**Distance** (A°)
**1**			
OH (ring A)	Hie305	H-bond	1.80
OH (B-ring)	Asp197	H-bond	1.59
B-ring	Tyr62	Pi-Pi stacking	4.30

**2**			
OH (A-ring)	Asp197	H-bond	1.72
OH (B-ring)	Asp300	H-bond	1.94
**3**			
OH (A ring)	Trp59	H-bond	2.28
OH (A ring)	Gln63	H-bond	2.20
B ring	Trp59	Pi-Pi stacking	3.87
OH (C ring)	Trp59	aromatic H-bond	3.32
C ring	Val163	aromatic H-bond	3.46
**4**			
OH (A ring)	Trp59	H-bond	2.61
B ring	Trp59	Pi-Pi stacking	3.87
OH (C ring)	Gln63	H-bond	2.15
OH (C-ring)	Trp59	aromatic H-bond	3.15
OH (D ring)	Gly106	H-bond	2.15
**5**			
B ring	Trp59	Pi-Pi stacking	4.11
B ring	Trp59	Pi-Pi stacking	4.13
**6**			

C ring	Trp59	Pi-Pi stacking	3.71
C ring	Trp59	Pi-Pi stacking	4.19
OH (B-ring)	Val163	H-bond	1.76
OH (A-ring)	Asn63	H-bond	2.72

Table S11. List of molecular interactions of **1** – **6** with α-Glu Catalytic Site.

**Ligands**	**Interacting residues**	**Interaction**	**Distance** (A°)
**1**			
OH (A-ring)	Asp349	H-bond	1.96
A-ring	Phe157	Pi-Pi stacking	5.46
A-ring	Phe300	Pi-Pi stacking	5.40
OH (B-ring)	Asp408	H-bond	1.76
B-ring	Arg312	Pi-cation	5.82
**2**			
A-ring	Phe157	Pi-Pi stacking	5.27
A-ring	Phe300	Pi-Pi stacking	4.94
(OH) B-ring	Glu304	H-bond	1.87
B-ring	Arg312	Pi-cation	6.11
**3**			
OH (D-ring)	Pro309	H-bond	1.77
OH (B-ring)	Thr307	H-bond	2.75

OH (C-ring)	Glu304	H-bond	2.68
C-ring	Phe157	aromatic H-bond	3.43
D-ring	Arg312	Pi-cation	5.59
**4**			
A-ring	Phe157	Pi-Pi stacking	5.22
OH (A-ring)	Phe157	aromatic H-bond	3.37
OH (B-ring)	Pro309	H-bond	2.00
OH (C-ring)	Glu304	H-bond	1.68
C-ring	Hip279	Pi-cation	5.07
C-ring	Hie239	Pi-Pi stacking	4.96
OH (D-ring)	Pro309	H-bond	1.83
D-ring	Phe231	Pi-Pi stacking	5.07
**5**			
OH (A-ring)	Asp349	H-bond	2.02
A-ring	Phe300	Pi-Pi stacking	5.12
B-ring	Arg312	Pi-cation	5.37
OH (C-ring)	Arg312	H-bond	2.56
C-ring	Phe157	aromatic H-bond	3.75
OH (D-ring)	Thr307	H-bond	1.97

D-ring	Thr307	aromatic H-bond	3.43
**6**			
OH (A-ring)	Asp408	H-bond	1.84
A-ring	Asp408	aromatic H-bond	3.25
A-ring	Arg312	Pi-cation	5.68
B-ring	Hie239	Pi-Pi stacking	5.44
B-ring	Phe157	aromatic H-bond	3.42
OH (C-ring)	Thr307	H-bond	2.72
OH (D-ring)	Pro309	H-bond	1.76
